# C−X Bond Activation by Palladium: Steric Shielding versus Steric Attraction

**DOI:** 10.1002/chem.202201093

**Published:** 2022-06-16

**Authors:** Thomas Hansen, Xiaobo Sun, Marco Dalla Tiezza, Willem‐Jan van Zeist, Joost N. P. van Stralen, Daan P. Geerke, Lando P. Wolters, Jordi Poater, Trevor A. Hamlin, F. Matthias Bickelhaupt

**Affiliations:** ^1^ Department of Theoretical Chemistry Amsterdam Institute of Molecular and Life Sciences (AIMMS) Amsterdam Center for Multiscale Modeling (ACMM) Vrije Universiteit Amsterdam De Boelelaan 1083 1081 HV Amsterdam The Netherlands; ^2^ Leiden Institute of Chemistry Leiden University Einsteinweg 55 2333 CC Leiden The Netherlands; ^3^ Departament de Química Inorgànicai i Orgànica & IQTCUB Universitat de Barcelona Martí i Franquès 1-11 08028 Barcelona Spain; ^4^ ICREA Passeig Lluís Companys 23 08010 Barcelona Spain; ^5^ Institute for Molecules and Materials (IMM) Radboud University Heyendaalseweg 135 6525 AJ Nijmegen The Netherlands

**Keywords:** activation strain model, bond activation, density functional calculations, homogeneous catalysis, oxidative addition, reactivity

## Abstract

The C−X bond activation (X = H, C) of a series of substituted C(n°)−H and C(n°)−C(m°) bonds with C(n°) and C(m°) = H_3_C− (methyl, 0°), CH_3_H_2_C− (primary, 1°), (CH_3_)_2_HC− (secondary, 2°), (CH_3_)_3_C− (tertiary, 3°) by palladium were investigated using relativistic dispersion‐corrected density functional theory at ZORA‐BLYP‐D3(BJ)/TZ2P. The effect of the stepwise introduction of substituents was pinpointed at the C−X bond on the bond activation process. The C(n°)−X bonds become substantially weaker going from C(0°)−X, to C(1°)−X, to C(2°)−X, to C(3°)−X because of the increasing steric repulsion between the C(n°)‐ and X‐group. Interestingly, this often does not lead to a lower barrier for the C(n°)−X bond activation. The C−H activation barrier, for example, decreases from C(0°)−X, to C(1°)−X, to C(2°)−X and then increases again for the very crowded C(3°)−X bond. For the more congested C−C bond, in contrast, the activation barrier always increases as the degree of substitution is increased. Our activation strain and matching energy decomposition analyses reveal that these differences in C−H and C−C bond activation can be traced back to the opposing interplay between steric repulsion across the C−X bond versus that between the catalyst and substrate.

## Graphical abstract

**Bulk up**! Our computational study shows how increasing steric bulk at the C−X bond systematically decreases the bond strength along the series C(0°)−X to C(1°)−X to C(2°)−X to C(3°)−X. These weaker C−H and C−C bonds are not always more reactive in oxidative addition reactions with palladium catalysts. Instead, we illustrate the interplay between steric repulsion across the C−X bond versus that of the catalyst and substrate.

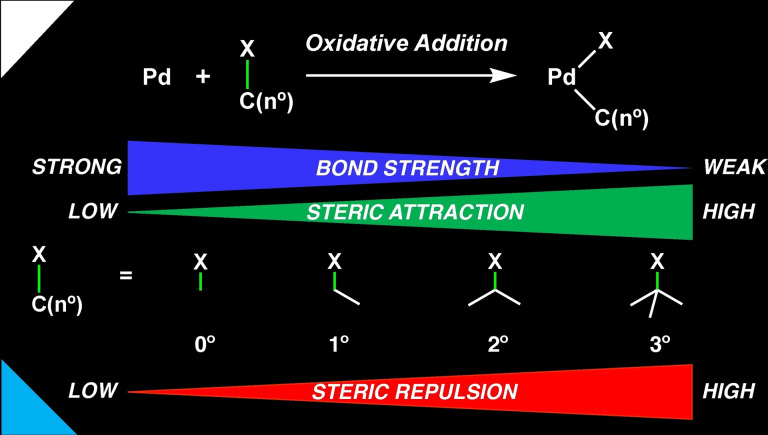

## Introduction

**Scheme 1:**
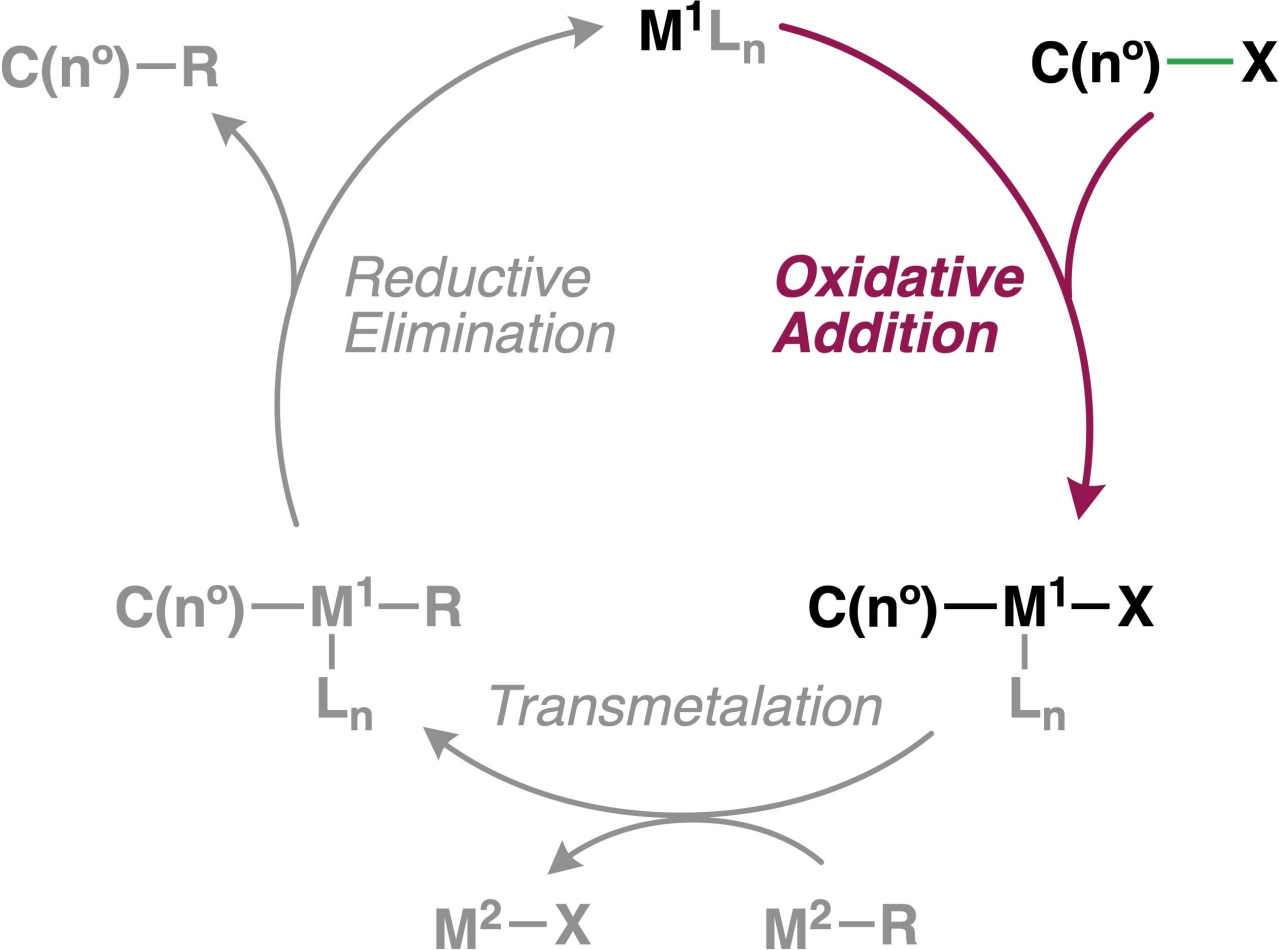
Schematic catalytic cycle of transition‐metal‐catalysed cross‐coupling reactions.

Transition‐metal‐catalysed cross‐coupling reactions are a convenient strategy to forge C−C bonds (Scheme 1).^[1]^ The first and generally rate‐determining step in the catalytic cycle of a typical cross‐coupling reaction by palladium is the oxidative addition, which can be described as the C−X bond activation.^[2]^ This step is key for the reactivity and selectivity of the overall catalytic cycle, and therefore has been extensively studied using both experimental and computational methods.^[3]^ The nature of the substrate to be activated has a profound effect on both the reactivity and selectivity of the process.^[4]^ However, quantitative insight on the effects of the degree of substitution at the activating bond of the substrate for the bond activation process is largely lacking.^[5]^

**Scheme 2:**
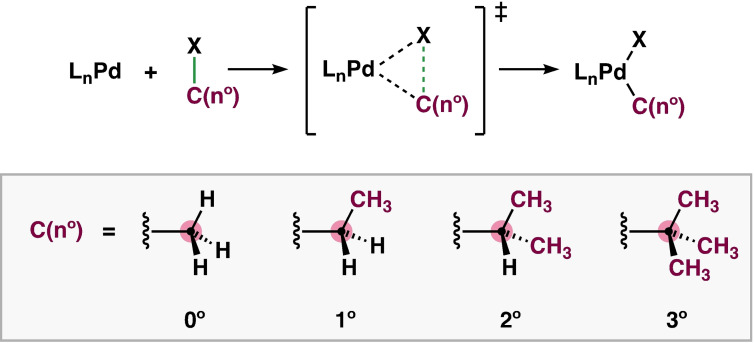
Studied oxidative addition reactions of C(n°)−X with X = H, C(m°) in model substrates H_3_C−X (methyl, 0°), CH_3_H_2_C−X (primary, 1°), (CH_3_)_2_HC−X (secondary, 2°), and (CH_3_)_3_C−X (tertiary, 3°), using PdL_n_ with L_n_ = no ligand, Cl^−^, (PH_3_)_2_.

In general, a higher degree of substitution around a chemical bond leads to a substantially weaker and longer C−X bond.^[6]^ Intuitively, the introduction of steric bulk at the C−X bond can have two distinct effects on the catalyst–substrate interactions: (i) one mechanism is the classical steric repulsion deriving from the overlap between closed‐shell orbitals of bulky substituents; (ii) the second mechanism embodies steric attraction, which occurs as a result of dispersion interactions between substituents and the catalyst that are not yet in direct contact. To get quantitative insight into the effects of the degree of substitution at C(n°)−X (X = H, C) on the reactivity of the oxidative addition, we have computationally studied the reaction profiles of the bond activation of C(n°)−H and C(n°)−C(m°) bonds with C(n°) and C(m°) = H_3_C− (methyl, 0°), CH_3_H_2_C− (primary, 1°), (CH_3_)_2_HC− (secondary, 2°), (CH_3_)_3_C− (tertiary, 3°), using relativistic dispersion‐corrected density functional theory at ZORA‐BLYP‐D3(BJ)/TZ2P (Scheme 2; see Supporting Information for computational details). We used PdL_n_ with L_n_ = no ligand, Cl^−^, and (PH_3_)_2_ as model catalysts for the bond activation.

The activation strain model (ASM)^[7]^ and Kohn‐Sham molecular orbital (KS‐MO)^[8a]^ theory in combination with our canonical energy decomposition analysis (EDA)^[8b]^ were employed to provide a quantitative understanding into the effects of the degree of substitution at C(n°)−X on the oxidative addition. This methodological approach enables the investigation of the activation barrier by decomposing the total energy of the system into chemically and physically meaningful and intuitive terms. This methodological approach has proven to be invaluable for understanding a wide range of chemical reactions.[^5^, ^9^]

## Results and Discussion

### Structure and reactivity trends

First, the bond strength (i. e., Δ*H*_BDE_) of the activated bonds in the substrates was analysed, which is important for overall C(n°)−X bond activation (see below). Table 1 summarizes the computed dissociation enthalpies of the studied C−X bonds at ZORA‐BLYP‐D3(BJ)/TZ2P and in parentheses without dispersion correction at ZORA‐BLYP/TZ2P. In line with experimental work,^[10]^ for both the C−H and C−C bond, we find that the bond strength for the activated C(n°)−X bond becomes systematically weaker when the C(n°)‐ and X‐group become substituted with sterically more demanding groups following H_3_C− (methyl, 0°) to CH_3_H_2_C− (primary, 1°) to (CH_3_)_2_HC− (secondary, 2°) to (CH_3_)_3_C− (tertiary, 3°), see Table 1. At the same time, the C(n°)−X bond of the substrate also becomes longer along this series, going from 1.094 Å to 1.101 Å and from 1.539 Å to 1.595 Å for the C−H and C−C bond, respectively, which is corroborated by experimental findings.^[11]^ These trends are found for both, computations including dispersion corrections and computations in which dispersion corrections have been switched off.

**Table 1:** Bond lengths (in Å) and homolytic bond dissociation enthalpies (in kcal mol^−1^) of C(n°)−X bonds with X = H, C(m°) in our bond‐activation reactions.^[a]^

C(n°)−X	r C−X	Δ*H*_BDE_
H_3_C−H	1.094 (1.094)	102.6 (102.2)
CH_3_H_2_C−H	1.097 (1.097)	97.7 (97.1)
(CH_3_)_2_HC−H	1.099 (1.100)	93.9 (93.0)
(CH_3_)_3_C−H	1.101 (1.102)	91.2 (90.0)
H_3_C−CH_3_	1.539 (1.539)	85.2 (83.1)
CH_3_H_2_C−CH_3_	1.538 (1.540)	82.7 (79.7)
(CH_3_)_2_HC−CH_3_	1.541 (1.544)	80.3 (76.3)
(CH_3_)_3_C−CH_3_	1.546 (1.550)	78.1 (72.9)
CH_3_H_2_C−CH_2_CH_3_	1.540 (1.542)	80.1 (76.1)
(CH_3_)_2_HC−CH(CH_3_)_2_	1.558 (1.562)	73.0 (65.5)
(CH_3_)_3_C−C(CH_3_)_3_	1.595 (1.607)	64.0 (51.8)

[a] Computed at ZORA‐BLYP‐D3(BJ)/TZ2P and ZORA‐BLYP/TZ2P in parentheses, at 298.15 K and 1 atm.

In line with the work of Gronert, an increase of the degree of substitution at C−X induces a more destabilizing steric (Pauli) repulsion between the C‐ and X‐group, which causes the bond to elongate and weaken.[^6^, ^12^] Around the same time, several other research groups emphasized that also the steric attraction between the substituents plays a key role in these bonds, stemming from the stabilizing dispersion forces.[^11^, ^13^] We agree that both of these opposing effects are, indeed, fundamental features in C−X bonds, however, for our studied systems, the steric (Pauli) repulsion dominates the trend in bond strength and is only slightly attenuated by the steric attraction. Note, that this interplay between steric attraction and repulsion is also important for the reactivity of the studied bond activations and will be discussed later.

In all cases, the C−H bond is substantially stronger than the C−C bond with the same degree of substitution. Importantly, the C−H bond becomes relatively more weakened along C(0°)−H, C(1°)−H, C(2°)−H, C(3°)−H going from Δ*H*_BDE_=102.6, to 97.7, to 93.9, to 91.2 kcal mol^−1^, while the C−C bond only goes from Δ*H*_BDE_=85.2, to 82.7, to 80.3, to 78.1 kcal mol^−1^ along C(0°)−CH_3_, C(1°)−CH_3_, C(2°)−CH_3_, C(3°)−CH_3_. This exact same behaviour is also found experimentally.^[10]^ The larger variation in C−H than C−C bonds strength upon increasing the degree of substitution can be attributed to the intrinsically shorter C−H bond, which experiences more steric (Pauli) repulsion between the C‐ and H‐group.

Note that the bonds are weaker and longer if dispersion corrections are excluded in the computations. The differences for the C−H bonds are minimal (ΔΔ*H*_BDE_=0.4 to 1.2 kcal mol^−1^ weaker and Δr R−H of 0.0 to 0.001 Å longer with respect to the computations with dispersion corrections) along C(0°)−H, C(1°)−H, C(2°)−H, and C(3°)−H. In contrast, for the C−C bonds, this effect is more apparent (ΔΔ*H*_BDE_=2.1 to 5.2 kcal mol^−1^ weaker and Δr R−X=0.0 to 0.004 Å longer) along C(0°)−CH_3_, C(1°)−CH_3_, C(2°)−CH_3_, and C(3°)−CH_3_. Later on, we will discuss how the steric attraction between the substituents of C−H and C−C bonds (i. e., dispersion‐induced strengthening), in principle, is an effect that raises the bond‐activation barrier, but that is dominated by another dispersion‐induced effect that lowers the same barrier, namely, the stabilizing contribution of dispersion to the catalyst–substrate interaction.

**Figure 1:**
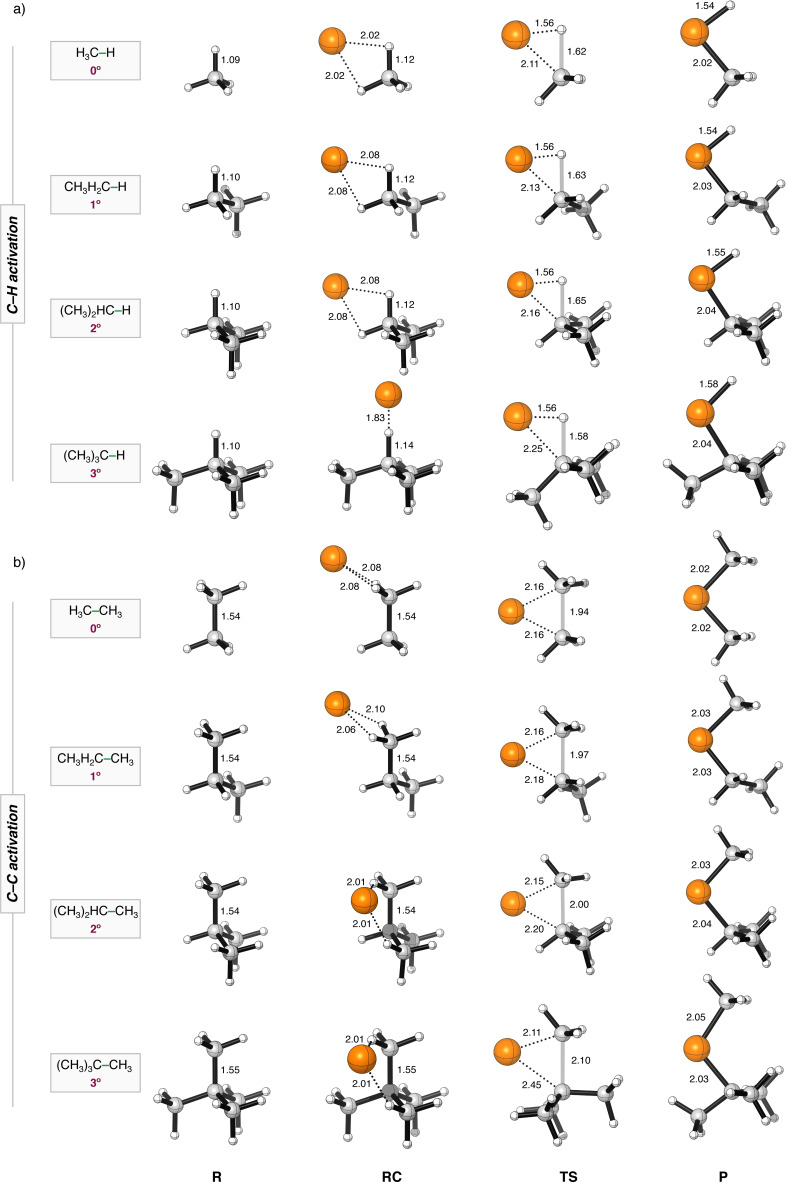
Stationary‐point structures (R = reactant, RC = reactant complex, TS = transition state, and P = product) in (a) C−H and (b) C−C activation between Pd+C(n°)−X (X = H, CH_3_) with C(n°)=H_3_C− (0°), CH_3_H_2_C− (1°), (CH_3_)_2_HC− (2°), and (CH_3_)_3_C− (3°) and X = H, CH_3_ with the key distances (in Å) computed at ZORA‐BLYP‐D3(BJ)/TZ2P. Atom colours: carbon (grey), hydrogen (white), palladium (orange).

The computed reaction profiles and structural data of the studied C(n°)−X activation reactions at ZORA‐BLYP‐D3(BJ)/TZ2P and ZORA‐BLYP/TZ2P are provided in Table 2 and Figure 1. Generally, the reactions proceed via a reactant complex (RC), and a transition state (TS), towards the product (P). Note, that the overall reaction barrier, Δ*E* ^≠^, that is, the energy difference between the TS and the infinitely separated reactants, can be negative if a substantially stabilized reactant complex is formed. This typically happens in apolar, weakly solvating solvents and, especially, in the gas phase (see for example Ref. [14] for a more detailed discussion).

**Table 2:** Energies relative to the separate reactants (in kcal mol^−1^) of the stationary points of the C(n°)−X bond activation along the PES.^[a]^

Activation Bond	Pd‐catalyst	Substrate	RC	TS	P
C−H	Pd	H_3_C−H	−10.4 (−6.6)	1.0 (4.1)	−6.1 (−3.0)
		CH_3_H_2_C−H	−11.6 (−6.7)	0.3 (4.6)	−8.0 (−3.7)
		(CH_3_)_2_HC−H	−12.6 (−6.5)	0.1 (5.5)	−9.6 (−4.2)
		(CH_3_)_3_C−H	−12.0 (−6.4)	2.6 (9.5)	−11.8 (−5.1)
	PdCl^−^	H_3_C−H	−15.1 (−12.1)	−8.2 (−5.0)	−11.4 (−8.2)
		CH_3_H_2_C−H	−16.1 (−12.3)	−8.5 (−4.2)	−12.1 (−7.6)
		(CH_3_)_2_HC−H	−17.4 (−12.6)	−9.1 (−3.6)	−13.5 (−7.4)
		(CH_3_)_3_C−H	−12.6 (−7.7)	−7.2 (−0.2)	−15.0 (−7.5)
	Pd(PH_3_)_2_	H_3_C−H	−2.1 (0.0^[b]^)	26.8 (32.7)	21.1 (27.7)
		CH_3_H_2_C−H	−3.3 (0.0^[b]^)	26.6 (34.3)	21.6 (29.5)
		(CH_3_)_2_HC−H	−4.2 (0.0^[b]^)	27.0 (35.7)	20.9 (30.4)
		(CH_3_)_3_C−H	−5.0 (0.0^[b]^)	29.0 (40.2)	21.7 (33.8)
C−C	Pd	H_3_C−CH_3_	−11.6 (−6.7)	13.8 (18.7)	−12.9 (−8.7)
		CH_3_H_2_C−CH_3_	−12.2 (−6.8)	14.3 (20.1)	−13.1 (−8.2)
		(CH_3_)_2_HC−CH_3_	−14.1 (−5.4)	14.7 (21.4)	−13.8 (−8.2)
		(CH_3_)_3_C−CH_3_	−15.1 (−6.4)	23.1 (30.6)	−15.0 (−9.3)
		CH_3_H_2_C−CH_2_CH_3_	−14.9 (−7.1)	14.8 (21.5)	−13.4 (−7.6)
		(CH_3_)_2_HC−CH(CH_3_)_2_	−17.8 (−6.8)	19.2 (27.7)	−16.7 (−10.1)
		(CH_3_)_3_C−C(CH_3_)_3_	−22.3 (−7.0)	34.5 (43.1)	−22.3 (−17.1)
	PdCl^−^	H_3_C−CH_3_	−16.1 (−12.3)	10.9 (15.9)	−13.8 (−9.1)
		CH_3_H_2_C−CH_3_	−16.7 (−12.6)	11.0 (17.1)	−14.4 (−8.6)
		(CH_3_)_2_HC−CH_3_	−17.1 (−12.4)	11.1 (18.0)	−12.9 (−6.4)
		(CH_3_)_3_C−CH_3_	−18.2 (−12.6)	19.9 (28.1)	−14.3 (−6.9)
		CH_3_H_2_C−CH_2_CH_3_	−18.2 (−12.2)	11.3 (18.4)	−12.4 (−8.0)
		(CH_3_)_2_HC−CH(CH_3_)_2_	−17.0 (−12.1)	15.9 (24.9)	−12.7 (−6.1)
		(CH_3_)_3_C−C(CH_3_)_3_	−20.3 (−12.8)	36.6 (46.3)	−19.0 (−11.2)
	Pd(PH_3_)_2_	H_3_C−CH_3_	−3.1 (0.0^[b]^)	43.2 (51.8)	17.6 (27.1)
		CH_3_H_2_C−CH_3_	−4.7 (0.0^[b]^)	43.4 (53.2)	18.7 (29.5)
		(CH_3_)_2_HC−CH_3_	−5.0 (0.0^[b]^)	44.3 (55.3)	19.5 (31.9)
		(CH_3_)_3_C−CH_3_	−5.5 (0.0^[b]^)	50.2 (63.2)	23.0 (37.1)
		CH_3_H_2_C−CH_2_CH_3_	−5.6 (0.0^[b]^)	42.9 (54.5)	19.9 (32.1)
		(CH_3_)_2_HC−CH(CH_3_)_2_	−6.5 (0.0^[b]^)	47.2 (61.1)	24.0 (38.6)
		(CH_3_)_3_C−C(CH_3_)_3_	−7.7 (0.0^[b]^)	59.3 (73.7)	27.1 (42.7)

[a] Electronic energies computed at ZORA‐BLYP‐D3(BJ)/TZ2P and ZORA‐BLYP/TZ2P in parentheses. [b] RC is unbound.

Several distinct trends emerge from the computed reaction profiles. Firstly, in all cases, the bond activation of the C−C bonds follows a significantly higher reaction barrier than that of the corresponding C−H bond (ΔΔ*E* ^≠^=+12.8 to +20.5 kcal mol^−1^ for C−C relative to C−H bond activation with the same degree of substitution), which is in sharp contrast to their intrinsic bond strength (see below). For the C−H bond, which is inherently less crowded from one side than the C−C bond, the reaction barrier decreases, at first, upon increasing the degree of substitution along the series from C(0°)−H, to C(1°)−H, to C(2°)−H. However, introducing sufficient steric bulk does ultimately raise the reaction barrier for the very bulky C(3°)−H. For example, for Pd+C(n°)−H, the reaction barrier goes slightly down from +1.0, to +0.3, to +0.1, and then up to +2.6 kcal mol^−1^ along C(n°)=H_3_C− (0°), CH_3_H_2_C− (1°), (CH_3_)_2_HC− (2°), (CH_3_)_3_C− (3°), see Table 2.

For the C−C bond, which is inherently sterically more congested, the reaction barrier for all computed bond activations always increases upon increasing the degree of substitution. For Pd+C(n°)−CH_3_, the reaction barrier increases monotonically and more steeply going from +13.8, to +14.3, to +14.7, to +23.1 kcal mol^−1^ along C(0°)−CH_3_, C(1°)−CH_3_, C(2°)−CH_3_, C(3°)−CH_3_. If the substitution of both carbon atoms, C(n°) and C(m°), increases simultaneously going from C(0°)−C(0°), to C(1°)−C(1°), to C(2°)−C(2°), to C(3°)−C(3°), the C−C activation barrier increases even more steeply, going from +13.8, to +14.8, to +19.2, to +34.5 kcal mol^−1^ (see Table 2). These reactivity trends are found regardless of whether we use bare palladium or palladium with ligands. Moreover, in all cases, the reaction barrier for the bond activation decreases as the model catalyst PdL_n_ goes from Pd(PH_3_)_2_, to Pd, to PdCl^−^. As previously found, the systematically higher reaction barriers for Pd(PH_3_)_2_, compared to bare Pd, stems from the increase in steric repulsion between the substrate and ligands of the catalyst, which require to bend away to allow the substrate to approach.^[5k]^ While, the lower reaction barrier for PdCl^−^ can be ascribed to the raise in the Pd‐4*d* derived orbitals in PdCl^−^, which translates into more stabilizing donor‐acceptor orbital interactions between the metal and the substrate.^[5e]^

Note that if dispersion corrections are excluded, all reaction barriers are increased, which is most apparent for the higher substituted bonds. For example, without dispersion correction at ZORA‐BLYP/TZ2P, the barrier for C−C bond activation by Pd increases significantly, from +18.7, to +20.1, to +21.4, to +30.6 kcal mol^−1^, going from C(0°)−CH_3_, to C(1°)−CH_3_, to C(2°)−CH_3_, to C(3°)−CH_3_, while this barrier rises significantly less steeply if dispersion corrections are included at ZORA‐BLYP‐D3(BJ)/TZ2P, namely, from +13.8, to +14.3, to +14.7, to +23.1 kcal mol^−1^ along the same series.

### Activation strain analyses

To gain insight into the factors controlling the reactivity of these oxidative addition reactions of C(n°)−X bonds, we turn to the activation strain model (ASM).^[7]^ The ASM is a fragment‐based approach in which the potential energy surface (PES) can be described with respect to, and understood in terms of the characteristics of, the reactants, i. e., the catalyst and substrate. One can decompose the total electronic energy (Δ*E*) into two separate terms: the strain energy (Δ*E*_strain_) and the interaction energy (Δ*E*_int_) by applying the ASM of reactivity (see Supporting Information for more details).

In the first place, we recall that C−C bond activation goes with a higher barrier than C−H bond activation. As found in our previous work,^[5b]^ we identify that this reactivity trend finds its origin in a delay in the build‐up of stabilizing interaction energy, along the reaction coordinate, between the catalyst and substrate for the C−C activation compared to the C−H (see Figure S1). This is the direct result of the inherently more congested nature of the C−C bond, which requires this bond to elongate more, to avoid destabilizing steric repulsion, before the metal can come closer and form stabilizing bonding overlap with the σ* of the substrate (see below).

**Figure 2:**
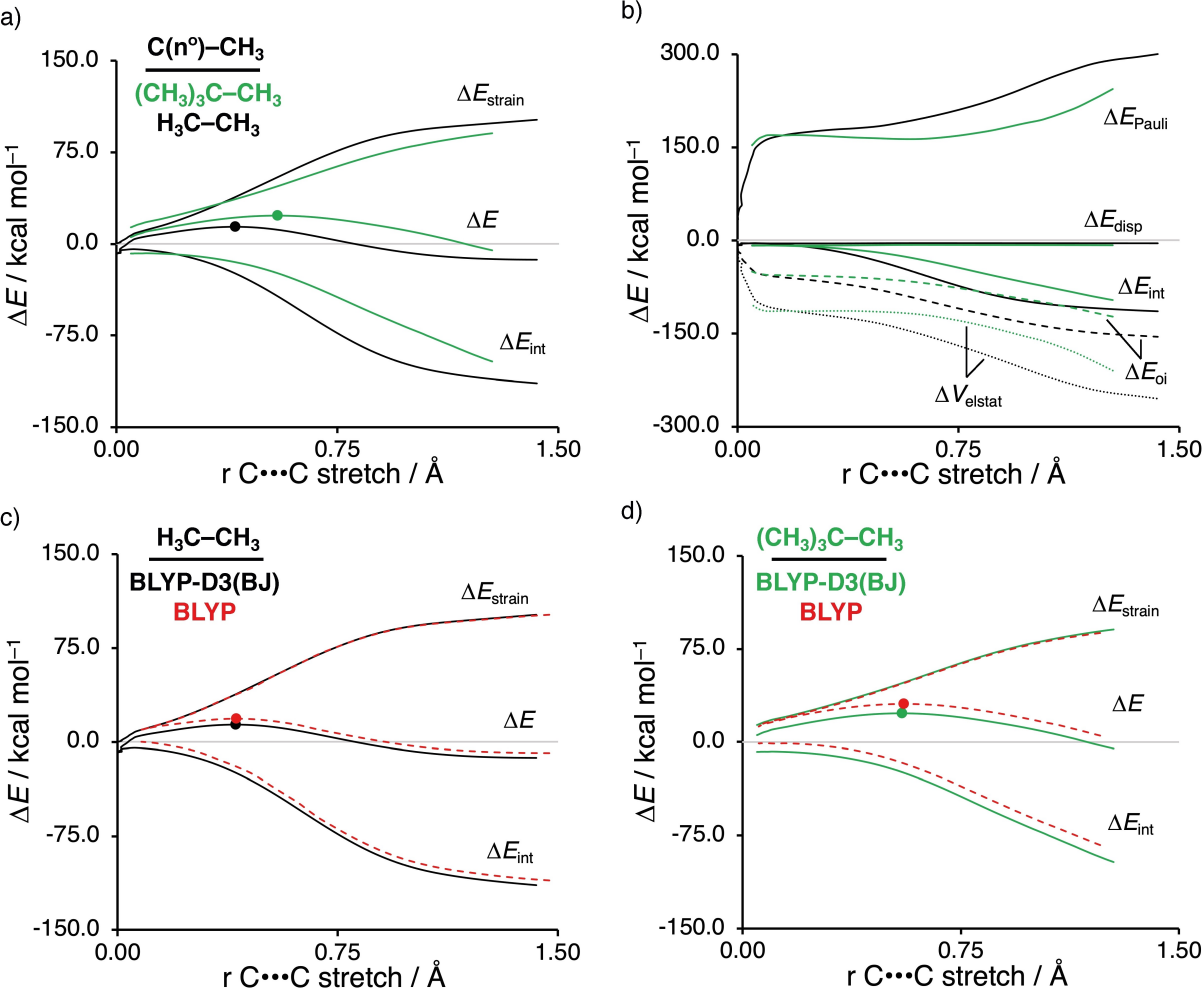
Activation strain diagrams (ASD: a, c, d) and energy decomposition analysis (EDA: b) of Pd‐induced C−C bond activation reactions along the IRC projected onto the C⋅⋅⋅C bond stretch. (a) ASD and (b) EDA for C−C bond activation of Pd+H_3_C−CH_3_ (black: methyl, 0°) and Pd+(CH_3_)_3_C−CH_3_ (green: tertiary, 3°) computed with dispersion effects at ZORA‐BLYP‐D3(BJ)/TZ2P. ASDs of (c) Pd+H_3_C−CH_3_ and (d) Pd+(CH_3_)_3_C−CH_3_ computed with and without dispersion effects at ZORA‐BLYP‐D3(BJ)/TZ2P and ZORA‐BLYP/TZ2P, respectively.

To understand the effect of the stepwise introduction of substituents at C−X on the bond activation process, we focus on the activation of H_3_C−CH_3_ and (CH_3_)_3_C−CH_3_ by Pd, however, all other systems share similar characteristics and can be found in Figure S2 and S3. We find that the delay effect in interaction energy continues to grow if steric shielding is increased for C−C bond activation (see Figure S4), and even is found for C−H bond activation but much less so. The activation strain diagram in Figure 2a shows that the barrier in the reaction profiles rises going from Pd‐mediated C−C bond activation of H_3_C−CH_3_ to that of (CH_3_)_3_C−CH_3_, which directly originates from a significant increase in the delay in building up stabilizing interaction Δ*E*_int_ between catalyst and substrate. In other words, the green interaction curve of the more hindered (CH_3_)_3_C−CH_3_ is above the black counterpart and hence effectively running behind, i. e., delayed, compared to H_3_C−CH_3_. This delay in catalyst–substrate interaction becomes even more pronounced as the degree of substitution further increases, for example, C(3°)−C(3°), as can be seen in Figure S3.

In contrast, the strain energy is less destabilizing for the more substituted (CH_3_)_3_C−CH_3_, because the bond becomes weaker as the degree of substitution increases, from H_3_C−CH_3_ (Δ*H*_BDE_=85.2 kcal mol^−1^) to (CH_3_)_3_C−CH_3_ (Δ*H*_BDE_=78.1 kcal mol^−1^; see Table 1). Note that at an early stage of the reaction coordinate, the strain energy is slightly more destabilizing for the more substituted (CH_3_)_3_C−CH_3_, which is the result of the required deformation, i. e., tilting of the methyl groups around the C−C bond, in order to facilitate the approach of the catalyst. Note that steric repulsion between the reactants can manifest in both: (i) the strain energy, because steric repulsion deforms the fragments, and (ii) the steric (Pauli) repulsion found in the catalyst–substrate interaction. As mentioned above, as the reaction proceeds and the C⋅⋅⋅C bond becomes longer, the strain curves eventually recover the expected bond dissociation energy trends reflecting the bond strength of the activated C−X bond. This trend can be found for all our studied systems. Altogether, increasing the degree of substitution of the C−C bond raises the barrier for palladium‐induced bond activation, because this substitution weakens the stabilizing catalyst–substrate interaction more than it alleviates the destabilizing strain.

To understand the role of dispersion effects in these bond activations, we have also explored and analysed the reaction pathways with dispersion corrections switched off, i. e., at ZORA‐BLYP/TZ2P. In principle, dispersion has two counteracting effects in these bond activation reactions: (i) raising of the activation barrier by strengthening the C−X bond; and (ii) lowering of the activation barrier by strengthening the catalyst–substrate interaction. Our activation strain analyses show that the barrier‐lowering effect, i. e., (ii), dominates, that is: dispersion reduces the barrier for bond activation in our set of model reactions. Thus, Figure 2c and Figure 2d show the activation strain diagrams (ASDs) of representative systems computed with dispersion correction (black and green) and without dispersion correction (red dashed lines). The ASDs show that switching on dispersion corrections stabilizes the catalyst–substrate interaction significantly more than it destabilizes the strain curve. The latter is in line with the fact that the C−X bond strength is only marginally enhanced by dispersion (see Table 1). The more stabilizing effect of dispersion forces in the case of the catalyst–substrate interaction can be ascribed, among others, to the higher polarizability of the palladium atom compared to carbon and hydrogen.[^15^, ^16^]

Next, we address the delay in the build‐up of stabilizing interaction between catalyst and substrate. This is exemplified by the less stabilizing interaction Δ*E*_int_ for the (CH_3_)_3_C−CH_3_ bond activation compared to H_3_C−CH_3_. To understand this interaction difference, we employed our canonical energy decomposition analysis (EDA).^[8b]^ The EDA plot in Figure 2b shows that (CH_3_)_3_C−CH_3_ bond engages in significantly weaker electrostatic and orbital interactions with the catalyst. Counterintuitively, the sterically more shielded (CH_3_)_3_C−CH_3_ experiences less steric (Pauli) repulsion with the catalyst, not more. This is a consequence of the geometrical relaxation that is caused by an intrinsically, indeed, higher steric (Pauli) repulsion that leads to a substantially larger distance between the reactants (i. e., Pd⋅⋅⋅C^α^_substrate_ and Pd⋅⋅⋅C^β^_substrate_ distance; highlighted in blue in Table 3) for (CH_3_)_3_C−CH_3_ compared to H_3_C−CH_3_. For example, at a consistent geometry close to the transition state, at a C⋅⋅⋅C bond stretch of 0.45 Å [actual TS C⋅⋅⋅C bond stretch of C(0°)−CH_3_=0.40 Å; C(1°)−CH_3_=0.43 Å; C(2°)−CH_3_=0.46 Å; C(3°)−CH_3_=0.55 Å], taken directly from a point on the IRC, the distances for Pd+(CH_3_)_3_C^α^−C^β^H_3_ (Pd⋅⋅⋅C^α^_substrate_=2.45 and Pd⋅⋅⋅C^β^_substrate_=2.14 Å) are considerably longer than for Pd+H_3_C^α^−C^β^H_3_ (Pd⋅⋅⋅C^α^_substrate_=2.16 and Pd⋅⋅⋅C^β^_substrate_=2.16 Å). Thus, only when the C−C bond is sufficiently elongated, can the Pd atom approach the C−C bond more closely without severe steric (Pauli) repulsion and engage in stabilizing bonding HOMO–LUMO overlap between its 3d_π_ orbital and the σ*_C−C_ of the substrate (see also Figure S4). This is the underlying physical mechanism associated with the delayed interaction in bond‐activation reactions involving sterically shielded bonds.

**Table 3:** Activation strain and energy decomposition analyses (in kcal mol^−1^) at TS‐like geometries for the C(n°)−CH_3_ bond activation of Pd+H_3_C−CH_3_ (0°), CH_3_H_2_C−CH_3_ (1°), (CH_3_)_2_HC−CH_3_ (2°), and (CH_3_)_3_C−CH_3_ (3°).^[a]^

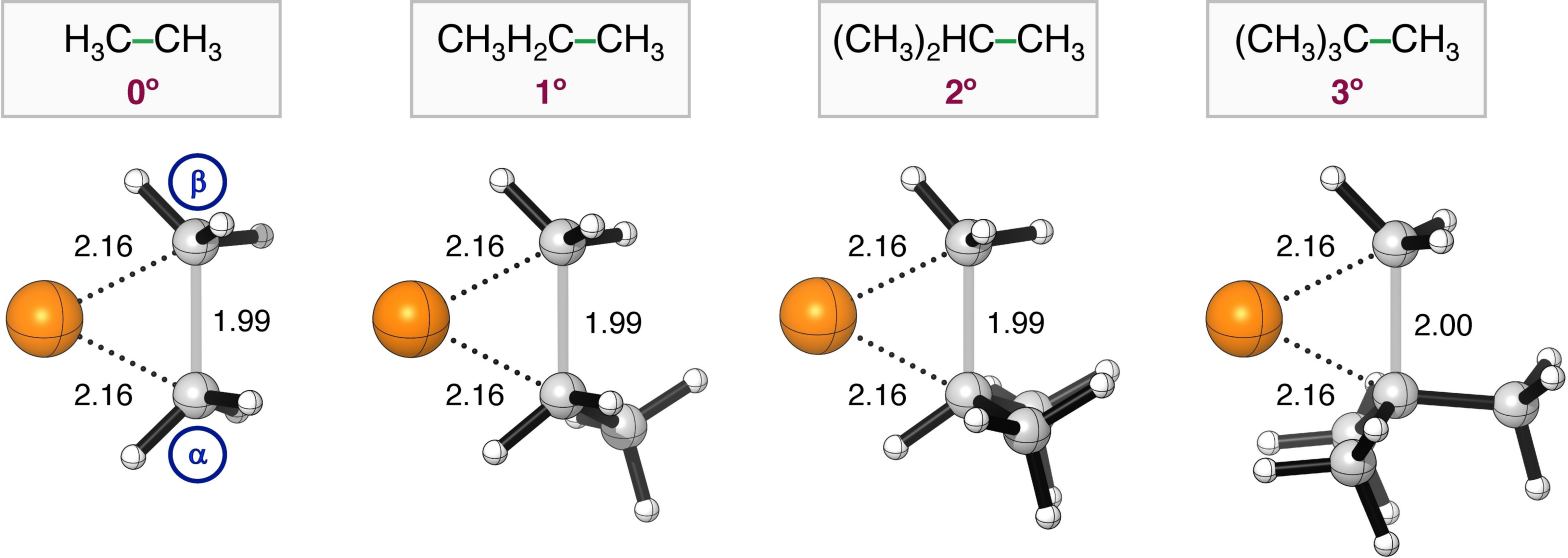							
**Substrate**	ΔE*	Δ*E*_strain_	Δ*E*_int_	Δ*V*_elstat_	Δ*E*_Pauli_	Δ*E*_oi_	Δ*E*_disp_
H_3_C−CH_3_	13.6	43.3	−29.7	−130.7	182.5	−76.7	−4.8
CH_3_H_2_C−CH_3_	14.4	42.0	−27.6	−135.4	189.4	−75.8	−5.8
(CH_3_)_2_HC−CH_3_	14.9	39.7	−24.8	−138.1	195.2	−75.1	−6.8
(CH_3_)_3_C−CH_3_	29.4	39.4	−10.0	−145.6	222.0	−77.7	−8.7

[a] Numerical experiment at double consistent TS‐like geometries (Δ*E* ^*^) obtained from the IRC at a C⋅⋅⋅C bond stretch of 0.45 Å. The Pd⋅⋅⋅C^α^_substrate_ and Pd⋅⋅⋅C^β^_substrate_ bond of CH_3_H_2_C−CH_3_, (CH_3_)_2_HC−CH_3_, and (CH_3_)_3_C−CH_3_ were both set to 2.16 Å, respectively (Pd⋅⋅⋅C^α^_substrate_ and Pd⋅⋅⋅C^β^_substrate_ distances in the consistent TS‐like geometry for H_3_C−CH_3_). Computed at ZORA‐BLYP‐D3(BJ)/TZ2P.

To further support this delay in interaction energy mechanism, and thus, accounting for the different catalyst–substrate distances for both systems, we perform a series of numerical experiments whereby a consistent geometry close to the transition state for H_3_C−CH_3_, CH_3_H_2_C−CH_3_, (CH_3_)_2_HC−CH_3_, (CH_3_)_3_C−CH_3_ at a C⋅⋅⋅C bond stretch of 0.45 Å is taken from the IRC. The Pd⋅⋅⋅C^α^_substrate_ and Pd⋅⋅⋅C^β^_substrate_ of CH_3_H_2_C−CH_3_, (CH_3_)_2_HC−CH_3_, (CH_3_)_3_C−CH_3_ is shortened to that of H_3_C−CH_3_ (2.16 Å), while maintaining a C⋅⋅⋅C bond stretch of 0.45 Å (see Table 3 for structures). Note that these geometries are not optimized, instead, they are taken from the IRC, and key bond distances are constrained to match a selected reference structure, which in this case is that of H_3_C−CH_3_.

Table 3 shows the results of the above‐described numerical experiment (see Table S1 for data of all the other studied systems), which share the same characteristics and, indeed, now the less stabilizing interaction energy along the series C(0°)−CH_3_, to C(1°)−CH_3_, to C(2°)−CH_3_, to C(3°)−CH_3_, i. e., Δ*E*_int_=−29.7, −27.6, −24.8, −10.0 kcal mol^−1^, can be exclusively traced back to the more destabilizing steric (Pauli) repulsion along this series. The steric (Pauli) repulsion becomes increasingly destabilizing along this series, going from 182.5, to 189.4, to 195.2, to 222.0 kcal mol^−1^. To pinpoint the origin of the more destabilizing steric (Pauli) repulsion for the more substituted C(n°)−CH_3_ bonds, we have performed a Kohn‐Sham molecular orbital analysis.^[8a]^ All occupied‐occupied orbital overlaps of Pd with H_3_C−CH_3_ (0°), CH_3_H_2_C−CH_3_ (1°), (CH_3_)_2_HC−CH_3_ (2°), and (CH_3_)_3_C−CH_3_ (3°) were quantified at the same geometries as our numerical experiments shown in Table 3 with a C⋅⋅⋅C bond stretch of 0.45 Å (see Supporting Information Figure S5 for data). Intuitively, each additional methyl group substituent added at the carbon of the C−X bond leads to more filled σ‐orbitals at the substrate. We find that the larger destabilizing steric (Pauli) repulsion for the more substituted bonds is caused by a cumulative effect of the increased number of filled σ‐orbital delocalized over the substrate having destabilizing overlap with the 3d orbitals of Pd. In other words, the more destabilizing steric (Pauli) repulsion stems directly from the increased number of substituents at the carbon atom in the C(n°)−X bond along this series, causing more destabilizing steric repulsion between closed shells of the catalyst and closed shells of the substrate.

This confirms that for more substituted C−X bonds the catalyst waits, to avoid substantial steric repulsion with the substituent(s), until the C−X bond is sufficiently elongated before the catalyst can come closer and form bonding overlap with the σ*_C−X_ of the substrate. This results in a significant delay in building up stabilizing interaction energy between catalyst and substrate. On the contrary, as discussed earlier, the destabilizing strain energy Δ*E*_strain_ decreases when introducing more substituents at the C−X bond, effectively weakening the C−X bond. Counterintuitively, the strain energy decreases less steeply for the more substituted bonds going from 43.3, to 42.0, to 39.7, to 39.4 kcal mol^−1^ along C(0°)−CH_3_, C(1°)−CH_3_, C(2°)−CH_3_, C(3°)−CH_3_ (see Table 3). This subtle effect is more pronounced at the C−H activation, a feature that we will explain in greater detail later on.

Interestingly, the C−H activation also goes with an increasing delay of the building up of stabilizing interaction as the degree of substitution at the C−H bond increases (see Figure S2), to make room for the inserting palladium catalyst, similar to what we find for C−C activation. However, the delay effect is significantly less pronounced in the case of C−H than for C−C activation, because the C−H bond is less sterically shielded from the side of the H atom. To further consolidate the causalities behind the effects of increasing the degree of substitution at C(n°) on the C(n°)−H activation barrier, we performed the analogous numerical experiment with double‐consistent TS‐like geometries for the C−H activation as described in the previous section for C−C activation. Hence, we take the same consistent point along the IRC for all C−H activation reactions, namely, at a C⋅⋅⋅H bond stretch of 0.54 Å, which is at the same time also close to the transition states for each of these model reactions: Pd+H_3_C−H, CH_3_H_2_C−H, (CH_3_)_2_HC−H, and (CH_3_)_3_C−H [actual TS C⋅⋅⋅H bond of C(0°)−H=0.53, C(1°)−H=0.53, C(2°)−H=0.55 and C(3°)−H=0.49 Å]. Importantly, the Pd⋅⋅⋅C^α^_substrate_ and Pd⋅⋅⋅H^β^_substrate_ distances of CH_3_H_2_C−H, (CH_3_)_2_HC−H, and (CH_3_)_3_C−H are shortened to that of the H_3_C−H (Pd⋅⋅⋅C^α^_substrate_=2.11 Å and Pd⋅⋅⋅H^β^_substrate_=1.56 Å), while maintaining the aforementioned C⋅⋅⋅H bond stretch of 0.54 Å (see Table 4 for structures).^[17]^

**Table 4:** Activation strain and energy decomposition analyses (in kcal mol^−1^) at TS‐like geometries for the C(n°)−H bond activation of Pd+H_3_C−H (0°), CH_3_H_2_C−H (1°), (CH_3_)_2_HC−H (2°), and (CH_3_)_3_C−H (3°).^[a]^

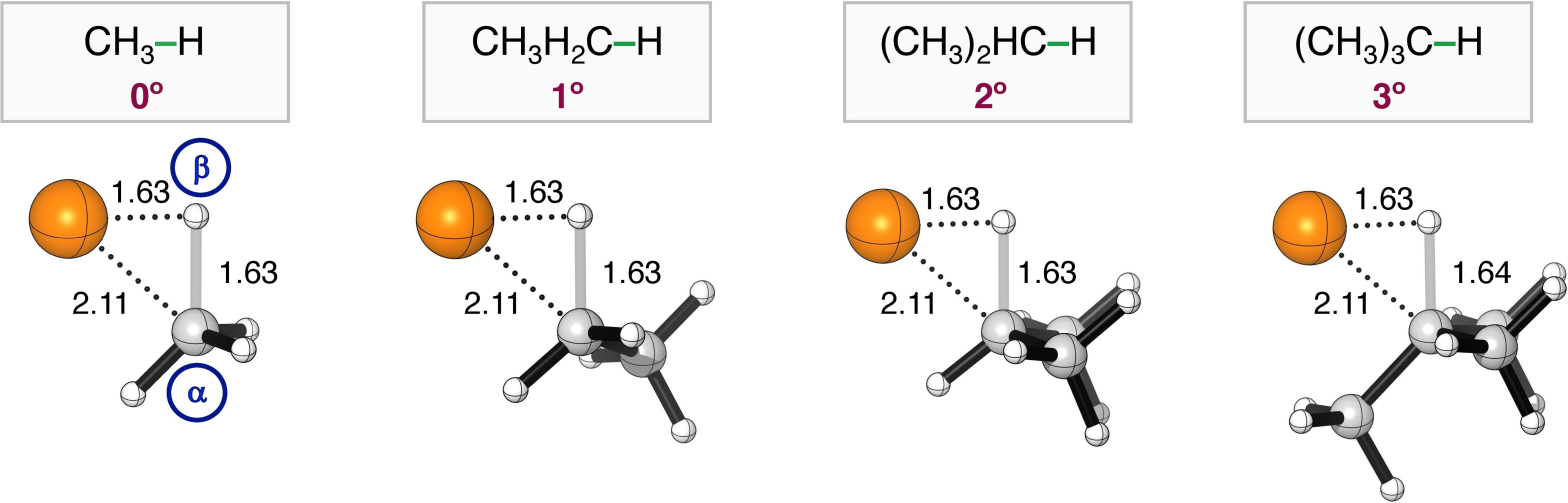							
**Substrate**	ΔE*	Δ*E*_strain_	Δ*E*_int_	Δ*V*_elstat_	Δ*E*_Pauli_	Δ*E*_oi_	Δ*E*_disp_
H_3_C−H	1.0	53.3	−52.3	−161.7	205.5	−93.3	−2.8
CH_3_H_2_C−H	0.4	51.5	−51.1	−168.7	213.3	−92.0	−3.7
(CH_3_)_2_HC−H	0.2	49.4	−49.2	−171.9	218.4	−91.1	−4.6
(CH_3_)_3_C−H	3.1	51.6	−48.5	−181.6	232.2	−93.0	−6.1

[a] Numerical experiment at double consistent TS‐like geometries (Δ*E* ^*^) obtained from the IRC at a C⋅⋅⋅H bond stretch of 0.54 Å. The Pd⋅⋅⋅C^α^_substrate_ and Pd⋅⋅⋅H^β^_substrate_ bond of CH_3_H_2_C−H, (CH_3_)_2_HC−H, (CH_3_)_3_C−H were set to 2.11 and 1.63 Å, respectively (Pd⋅⋅⋅C^α^_substrate_ and Pd⋅⋅⋅H^β^_substrate_ distances in the consistent TS‐like geometry for H_3_C−H). Computed at ZORA‐BLYP‐D3(BJ)/TZ2P.

These numerical experiments (ΔE* in Table 4) lead to the same reactivity trends as set by the actual TSs (Δ*E* ^≠^ in Table 2): ΔE* decreases from C(0°)−H to C(1°)−H to C(2°)−H and then increases for the C(3°)−H bond. In line with the C−C activation, we can conclude that the less stabilizing interaction energy along the series C(0°)−H, to C(1°)−H, to C(2°)−H_3_, to C(3°)−H, i. e., Δ*E*_int_=−52.3, −51.1, −49.2, −48.5 kcal mol^−1^, can be again solely ascribed to the more destabilizing steric (Pauli) repulsion along this series. By a Kohn‐Sham molecular orbital analysis (see Figure S6),^[8a]^ we could, like for the C−C bond activation, trace back the more destabilizing steric (Pauli) repulsion to a cumulative effect of the increased number of filled σ‐orbital delocalized over the substrate engaging in destabilizing overlap with the 3d orbitals of Pd. Similar to the situation for C−C activation, the strain energy becomes less destabilizing in agreement with the weakening of the C−H bond by substituting the C−H (see Table 1) with the exception of the bulkiest (CH_3_)_3_C−H system. We recall that steric repulsion can manifest in both, the strain energy and steric (Pauli) repulsion. At the computed geometry, the (CH_3_)_3_C−H is still in the process of the tilting away of the methyl substituents, which results in a more destabilizing strain than the less substituted systems that go with significantly less tilting (see Figure S2).

**Figure 3:**
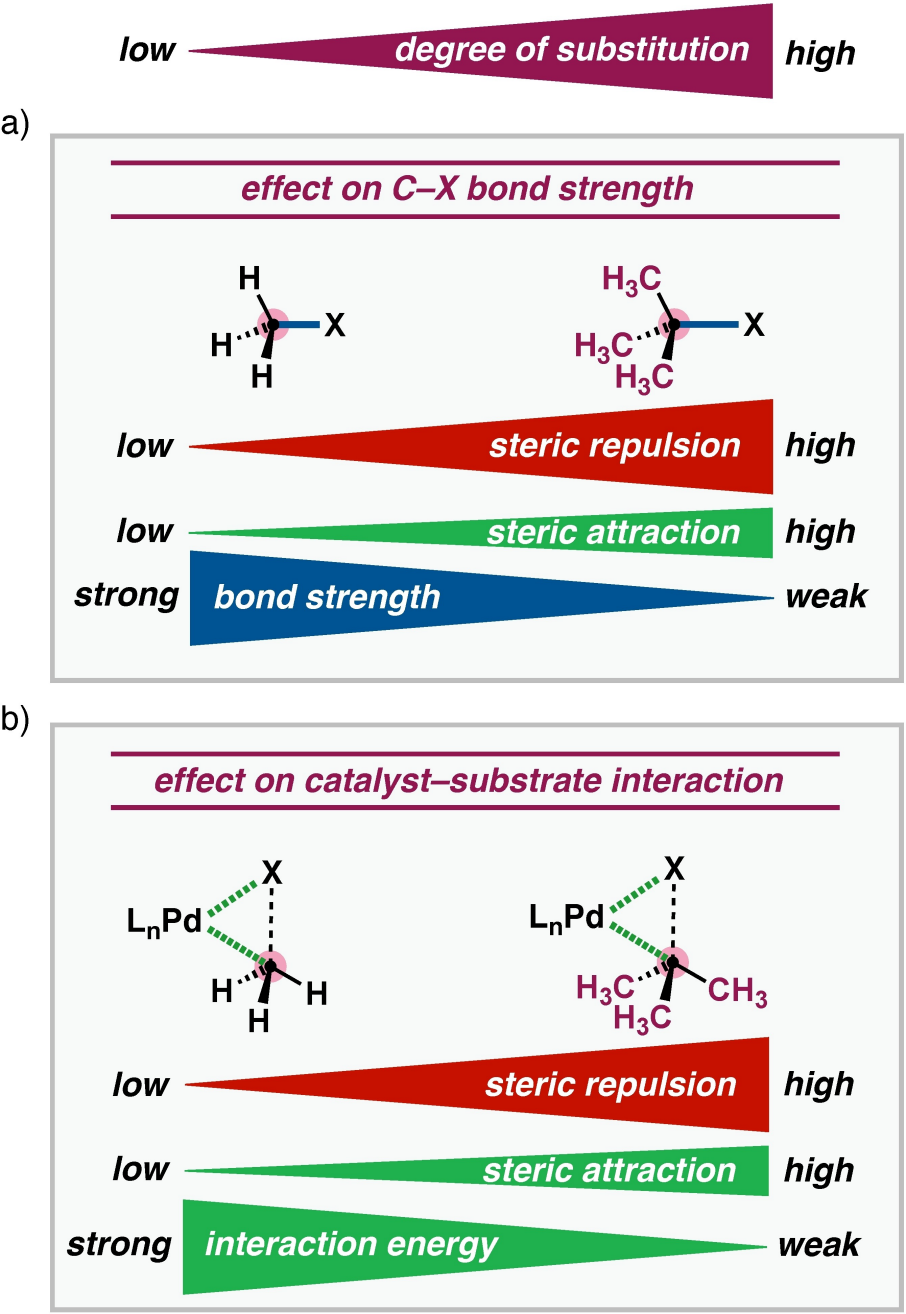
Influence of the stepwise introduction of substituents at C−X on the bond activation process, showing the opposing interplay between the effects across the activated bond (a; bond strength) versus that between the catalyst and substrate (b; interaction energy). For the C−C activation, the interaction is substantially weakened by introducing substituents at the C−X bond, which overrules the effect on the intrinsic bond strength, hence resulting in a higher reaction barrier. In contrast, for the C−H activation, generally, the interaction is not sufficiently weakened to overcome the effect on the trend in bond strength, and thus, follows a lower reaction barrier.

But then why does the C−C bond activation reaction barrier always increase while the C−H bond activation barrier initially decreases upon introducing more substituents in the substrate? The answer consists of two elements (Figure 3): both, C−C and C−H bonds, become weaker as the degree of substitution increases, a factor that works towards lowering the barrier through less activation strain Δ*E*_strain_. In the case of C−C bond activation, the catalyst–substrate interaction Δ*E*_int_ overrules this trend in strain reduction through a significant weakening in the interaction energy caused by a steep increase in steric (Pauli) repulsion as the steric shielding of the C−C bond increases. In the case of C−H bond activation, the corresponding weakening in Δ*E*_int_ is more subtle, because the catalyst can approach the C−H bond on the sterically unshielded H side, and therefore can no longer overrule the trend in the barrier lowering reduction of the strain Δ*E*_strain_. Interestingly, from C(2°)−H to C(3°)−H, the barrier does slightly increase, not because of a more pronounced weakening in catalyst–substrate interaction Δ*E*_int_ but because the substrate strain Δ*E*_strain_ begins to grow instead of further decreasing. This is a direct effect of the increased steric demand of the C(3°)−H bond, however, at this stage, the system is still mainly absorbing this hindrance into the Δ*E*_strain_ term as a result of the required tilting of the methyl substituents, and not in the steric (Pauli) term. The underlying physical mechanism at play is described in more detail below.

Thus, in the case of C−C bond activation, the catalyst–substrate interaction Δ*E*_int_ weakens more pronouncedly than in the case of C−H activation. The direct comparison of Tables 3 and 4 shows that, for the C−C bond activation, the interaction energy weakens more strongly, going from 0° to 1°, to 2°, to 3° substrates (ΔΔ*E*_int_=+2.1, +4.9, +19.7 kcal mol^−1^ with respect to H_3_C−CH_3_) than for the C−H bond activation (ΔΔ*E*_int_=+1.2, +3.1, +3.8 kcal mol^−1^ with respect to H_3_C−H).^[17]^ This is the direct result of the C−H bond being inherently less shielded at the side of its hydrogen atom, than the C−C bond which is surrounded by three substituents on both ends. This circumstance makes it possible for the catalyst to approach the C−H bond always from this substantially less hindered side. The difference in the extent to which the barrier in C−H and C−C bond activation is affected by steric bulk is slightly reinforced by the respective trends in strain energy. The strain energy, for the C−H bond activation, initially decreasing faster going from 0° to 1°, to 2°, to 3° substrate (ΔΔ*E*_strain_=−1.8, −3.9, −1.7 kcal mol^−1^ with respect to H_3_C−H) than for the C−C bond activation (ΔΔ*E*_strain_=−1.3, −3.6, −3.9 kcal mol^−1^ with respect to H_3_C−CH_3_). This is a direct effect of the trend in C(n°)−X bond strength, in which the C−H bond becomes relatively more weakened along C(0°)−H, C(1°)−H, C(2°)−H, and C(3°)−H than for the C−C bond (see Table 1).

## Conclusions

Our computational study reveals that for both, C(n°)−H and C(n°)−C(m°) bonds, the stepwise introduction of substituents at carbon systematically decreases the bond strength along n, m = 0–3 as a result of the repulsion across the C−X bond. In line with the overall weakening of the C(n°)−X bond, the reaction barrier generally decreases for C−H activation as the degree of substitution increases, and only rises when extremely bulky groups are introduced. In sharp contrast, for the more congested C−C bond, the reaction barrier always increases for higher substituted bonds. These trends emerge from our relativistic, dispersion‐corrected DFT computations.

The C−C bond activation always goes with a higher barrier than C−H bond activation, which finds its origin in the delay in the build‐up of stabilizing catalyst–substrate interaction between the catalyst and substrate for the C−C activation. This stems from the more congested nature of the C−C bond, which requires this bond to elongate more, to avoid destabilizing steric repulsion, before the metal can come closer and form stabilizing bonding overlap of its occupied *d* AO with the σ* acceptor orbital of the substrate.^[5b]^ Here, we find that the delay effect in catalyst–substrate interaction continues if steric shielding is increased for the C−C bond activation, which causes the systematic increase in reaction barrier for the bond activation process by the introduction of substituents at the C(n°)−X bond. The C−H bond is inherently less crowded from one side, which makes it possible for the catalyst to approach the substrate from the least hindered side. This makes the delay effect in interaction minimal by the introduction of substituents at the C(n°)−X bond, and thus, the reaction barrier initially decreases as a result of the weaker bonds and only rises for very sterically demanding C−H bond.

The steric attraction between the steric bulk of the substituents and the catalyst somewhat stabilizes all reaction barriers of the C−X bond activations, and they do so more in the case of the higher substituted bonds. The barrier‐lowering effect of steric attraction is, however, in all cases dominated by the barrier‐raising effect of steric (Pauli) repulsion. Steric attraction has in principle two counteracting effects on the height of the barrier: (i) raising because of stronger C−X bonds, caused by the dispersive forces across the C−X bond; (ii) lowering because of stronger catalyst–substrate interaction, as a result of the dispersion interactions between the catalyst and the substrate. We find that in our series of archetypal reactions, the lowering of the bond‐activation barriers dominates, and thus, the dispersive forces between the catalyst and substrate. The more stabilizing effect of dispersion forces in the case of the catalyst–substrate interaction can be ascribed, among others, to the higher polarizability of the palladium atom compared to carbon and hydrogen.

## Supporting Information

Additional computational results; Cartesian coordinates, energies, and the number of imaginary frequencies of all stationary points.

## Conflict of interest

The authors declare no conflict of interest.

1

## Supporting information

As a service to our authors and readers, this journal provides supporting information supplied by the authors. Such materials are peer reviewed and may be re‐organized for online delivery, but are not copy‐edited or typeset. Technical support issues arising from supporting information (other than missing files) should be addressed to the authors.

Supporting Information

## Data Availability

The data that support the findings of this study are available in the supplementary material of this article.
