# Supplementary material for: C−X Bond Activation by Palladium: Steric Shielding versus Steric Attraction
Source: Chemistry. 2022 Jun 16;28(44):e202201093. doi: 10.1002/chem.202201093 (PMC9401605; doi:10.1002/chem.202201093)
Supplement: Supplementary file 1 — Supporting Information [file CHEM-28-0-s001.pdf]

Chemistry–A European Journal

Supporting Information

C–X Bond Activation by Palladium: Steric Shielding versus Steric Attraction

Thomas Hansen, Xiaobo Sun, Marco Dalla Tiezza, Willem-Jan van Zeist, Joost N. P. van Stralen, Daan P. Geerke, Lando P. Wolters, Jordi Poater, Trevor A. Hamlin,* and F. Matthias Bickelhaupt*

Contents

Computational Methods

Figure S1. Activation strain analysis of the C–H and C–C bond activation between Pd + H_3C–H (blue) and Pd + CH_3–CH_3 (black), along the IRC projected on the C•••X bond stretch. Computed at ZORA-BLYP-D3(BJ)/TZ2P.

Figure S2. Activation strain analysis and energy decomposition analysis for the C–H activation of Pd (a,b), $PdCl^-$ (c,d) and $Pd(PH_3)_2$ (e,f) + C(n°)–H, along the IRC projected on the C•••H bond stretch. Computed at ZORA-BLYP-D3(BJ)/TZ2P.

Figure S3. Activation strain analysis and energy decomposition analysis for the C–C activation of Pd (a,b), $PdCl^-$ (c,d) and $Pd(PH_3)_2$ (e,f) + C(n°)–C(m°), along the IRC projected on the C•••C bond stretch. Computed at ZORA-BLYP-D3(BJ)/TZ2P.

Figure S4. Pd•••$C^{\alpha}_{substrate}$ and Pd•••$C^{\beta}_{substrate}$ distance along the IRC projected onto the C•••C bond stretch for the Pd-induced C–C bond activation reactions of Pd + H_3C–CH_3 (black: methyl, 0°) and Pd + $(CH_3)_3C$–CH_3 (green: tertiary, 3°). Computed at ZORA-BLYP-D3(BJ)/TZ2P.

Figure S5. Heatmap of all occupied–occupied orbital overlaps from the HOMO to HOMO–15 at double consistent TS-like geometries (ΔE*; see Table 3) for C–C bond activation by Pd obtained from the IRC at a C•••C bond stretch of 0.45 Å. The Pd•••$C^{\alpha}_{substrate}$ and Pd•••$C^{\beta}_{substrate}$ bond of CH_3H_2C–CH_3, $(CH_3)_2HC$–CH_3, and $(CH_3)_3C$–CH_3 were both set to 2.16 Å, respectively (Pd•••$C^{\alpha}_{substrate}$ and Pd•••$C^{\beta}_{substrate}$ distances in the consistent TS-like geometry for H_3C–CH_3). Computed at ZORA-BLYP-D3(BJ)/TZ2P.

Figure S6. Heatmap of all occupied–occupied orbital overlaps from the HOMO to HOMO–15 at double consistent TS-like geometries (ΔE*; see Table 4) for C–H bond activation by Pd obtained from the IRC at a C•••H bond stretch of 0.54 Å. The Pd•••$C^{\alpha}_{substrate}$ and Pd•••$H^{\beta}_{substrate}$ bond of CH_3H_2C–H, $(CH_3)_2HC$–H, and $(CH_3)_3C$–H were both set to 2.11 and 1.63 Å, respectively (Pd•••$C^{\alpha}_{substrate}$ and Pd•••$H^{\beta}_{substrate}$ distances in the consistent TS-like geometry for H_3C–H). Computed at ZORA-BLYP-D3(BJ)/TZ2P.

Table S1. Activation strain and energy decomposition analyses (in kcal mol^{-1}) at double consistent TS-like geometries for the for the C–C bond activation of Pd + C(n°)–C(m°) with C(n°) and C(m°) = H_3C– (0°), CH_3H_2C– (1°), $(CH_3)_2HC$– (2°), $(CH_3)_3C$– (3°).[a]

Table S2. Cartesian coordinates (in Å), energies (*E*, *H* and *G*, in kcal mol^{-1}), and number of imaginary vibrational frequencies (N_{imag}) of all stationary points of the C–C bond activation reactions in the gas phase, computed at ZORA-BLYP-D3(BJ)/TZ2P.

Table S3. Cartesian coordinates (in Å), energies (*E*, *H* and *G*, in kcal mol^{-1}), and number of imaginary vibrational frequencies (N_{imag}) of all stationary points of the C–H activation reactions in the gas phase, computed at ZORA-BLYP-D3(BJ)/TZ2P.

Table S4. Cartesian coordinates (in Å), energies (*E*, *H* and *G*, in kcal mol^{-1}), and number of imaginary vibrational frequencies (N_{imag}) all stationary points of the C–C activation reactions in the gas phase, computed at ZORA-BLYP/TZ2P.

Table S5. Cartesian coordinates (in Å), energies (E, H and G, in kcal mol^{-1}), and number of imaginary vibrational frequencies (N_{imag}) of all stationary points of the C–H activation reactions in the gas phase, computed at ZORA-BLYP/TZ2P.

References

Computational Methods

All density functional theory (DFT) calculations were performed using the Amsterdam Density Functional (ADF2019.302) software package.[1] The generalized gradient approximation (GGA) exchange-correlation functional BLYP was used for all computations, which consists of the Becke exchange, and the Lee–Yang–Parr (LYP) correlation functional.[2] In addition, dispersion effects have been included using the D3(BJ) approximation by Grimme *et al.*[3] This approach was extensively tested against *ab initio* reference benchmarks from hierarchical series up till CCSD(T).[4] Scalar relativistic effects are accounted for using the zeroth-order regular approximation (ZORA).[5] The basis set used, denoted TZ2P, is of triple-ζ quality for all atoms and has been improved by four sets of polarization functions.[6] The polarization functions are 2p and 3d on H, 3d and 4f on C, P, Cl, and 5p and 4f on Pd. No frozen core approximation has been employed. For all calculations, the accuracies of the fit scheme (Zlm fit) and the integration grid (Becke grid) were set to VERYGOOD.[7] No symmetry constraints were used for all computations. All calculated stationary points have been verified by performing a vibrational analysis calculation,[8] to be energy minima (no imaginary frequencies) or transition states (only one imaginary frequency). The character of the normal mode associated with the imaginary frequency of the transition state has been inspected to ensure that it is associated with the reaction of interest. The potential energy surfaces of the studied C(n°)–X bond activations were obtained by performing intrinsic reaction coordinate (IRC) calculations,[9] which, in turn, were analyzed using the PyFrag 2019 program.[10] The optimized structures were illustrated using CYLview.[11]

Activation Strain and Energy Decomposition Analysis

The activation strain model (ASM) of chemical reactivity,[12] also known as the distortion/interaction model,[13] is a fragment-based approach in which the potential energy surface (PES) can be described with respect to, and understood in terms of the characteristics of, the reactants, *i.e.*, the catalyst and substrate. It considers the rigidity of the reactants and to which extent they need to deform during the reaction, plus their capability to interact with each other as the reaction proceeds. With the help of this model, we decompose the total energy, $\Delta E(\zeta)$, into the strain and interaction energy, $\Delta E_{\text{strain}}(\zeta)$ and $\Delta E_{\text{int}}(\zeta)$, respectively, as a function of the reaction coordinate ζ [Eq. (1)].

$$\Delta E(\zeta) = \Delta E_{\text{strain}}(\zeta) + \Delta E_{\text{int}}(\zeta) \quad (1)$$

In this equation, the strain energy, $\Delta E_{\text{strain}}(\zeta)$, is the penalty that needs to be paid to deform the reactants from their equilibrium to the geometry they adopt during the reaction at the point ζ of the

reaction coordinate. On the other hand, the interaction energy, $\Delta E_{\text{int}}(\zeta)$, accounts for all the chemical interactions that occur between these two deformed reactants along the reaction coordinate.

The interaction energy between the deformed reactants can be further analyzed in terms of quantitative Kohn-Sham molecular orbital (KS-MO) theory together with a canonical energy decomposition analysis (EDA).[14] The EDA decomposes the $\Delta E_{\text{int}}(\zeta)$ into the following four energy terms [Eq. (2)]:

$$\Delta E_{\text{int}}(\zeta) = \Delta V_{\text{elstat}}(\zeta) + \Delta E_{\text{Pauli}}(\zeta) + \Delta E_{\text{oi}}(\zeta) + \Delta E_{\text{disp}}(\zeta) \quad (2)$$

Herein, $\Delta V_{\text{elstat}}(\zeta)$ is the classical electrostatic interaction between the unperturbed charge distributions of the (deformed) reactants and is usually attractive. The Pauli repulsion, $\Delta E_{\text{Pauli}}(\zeta)$, arises from the destabilizing interaction between same-spin electrons on either fragment due to the Pauli principle and is the origin of steric repulsion. The orbital interaction energy, $\Delta E_{\text{oi}}(\zeta)$, accounts for, amongst others, charge transfer between the fragments, such as HOMO–LUMO interactions. Lastly, the ΔE_{disp} term accounts for attractive dispersion interactions.

In the herein presented activation strain and accompanied energy decomposition diagrams, the intrinsic reaction coordinate (IRC) is projected onto the bond stretch of the activated C•••X (X = H, C) bond. This critical reaction coordinate undergoes a well-defined change during the reaction from the reactant complex via the transition state to the product and is shown to be a valid reaction coordinate for studying bond activation reactions.[4] Consistent geometries were taken from the IRC. To ensure that our analyses were not skewed by the step size of the IRC, identical structures for all considered systems, in terms of the reaction coordinate, were obtained by linear interpolation of two adjacent IRC points.

Thermochemistry

Bond enthalpies, *i.e.*, bond dissociation energies (BDE), are calculated at 298.15 K and 1 atm (ΔH_{BDE}) from electronic bond energies (ΔE) and vibrational frequencies using standard thermochemistry relations for an ideal gas [Eq. (3)].[15]

$$\Delta H_{\text{BDE}} = \Delta E + \Delta E_{\text{trans,298}} + \Delta E_{\text{rot,298}} + \Delta E_{\text{vib,0}} + \Delta(\Delta E_{\text{vib,0}})_{298} \quad (3)$$

Here, $\Delta E_{\text{trans,298}}$, $\Delta E_{\text{rot,298}}$, and $\Delta E_{\text{vib,0}}$ are the differences between the C–X substrate and the $C^{\bullet}$ and $X^{\bullet}$ radical, that results from breaking C–X bond, in translational, rotational, and zero-point vibrational energy, respectively. The last term, $\Delta(\Delta E_{\text{vib,0}})_{298}$, is the change in the vibrational energy difference when going from 0 K to 298.15 K.

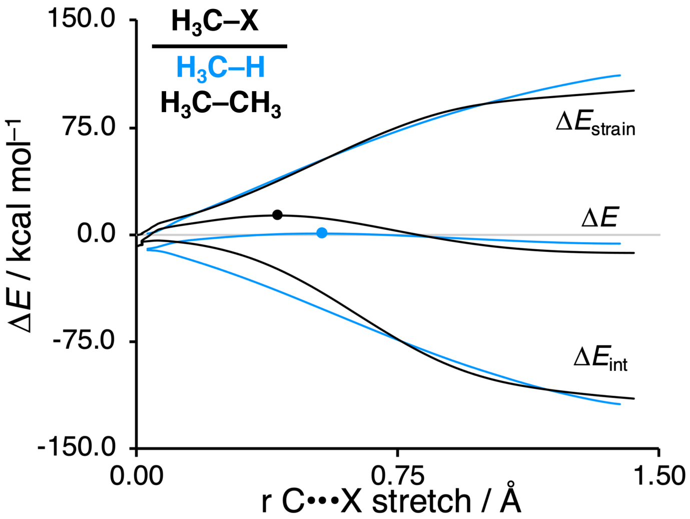

Figure S1. Activation strain analysis of the C–H and C–C bond activation between Pd + H_3C–H (blue) and Pd + CH_3–CH_3 (black), along the IRC projected on the C•••X bond stretch. Computed at ZORA-BLYP-D3(BJ)/TZ2P.

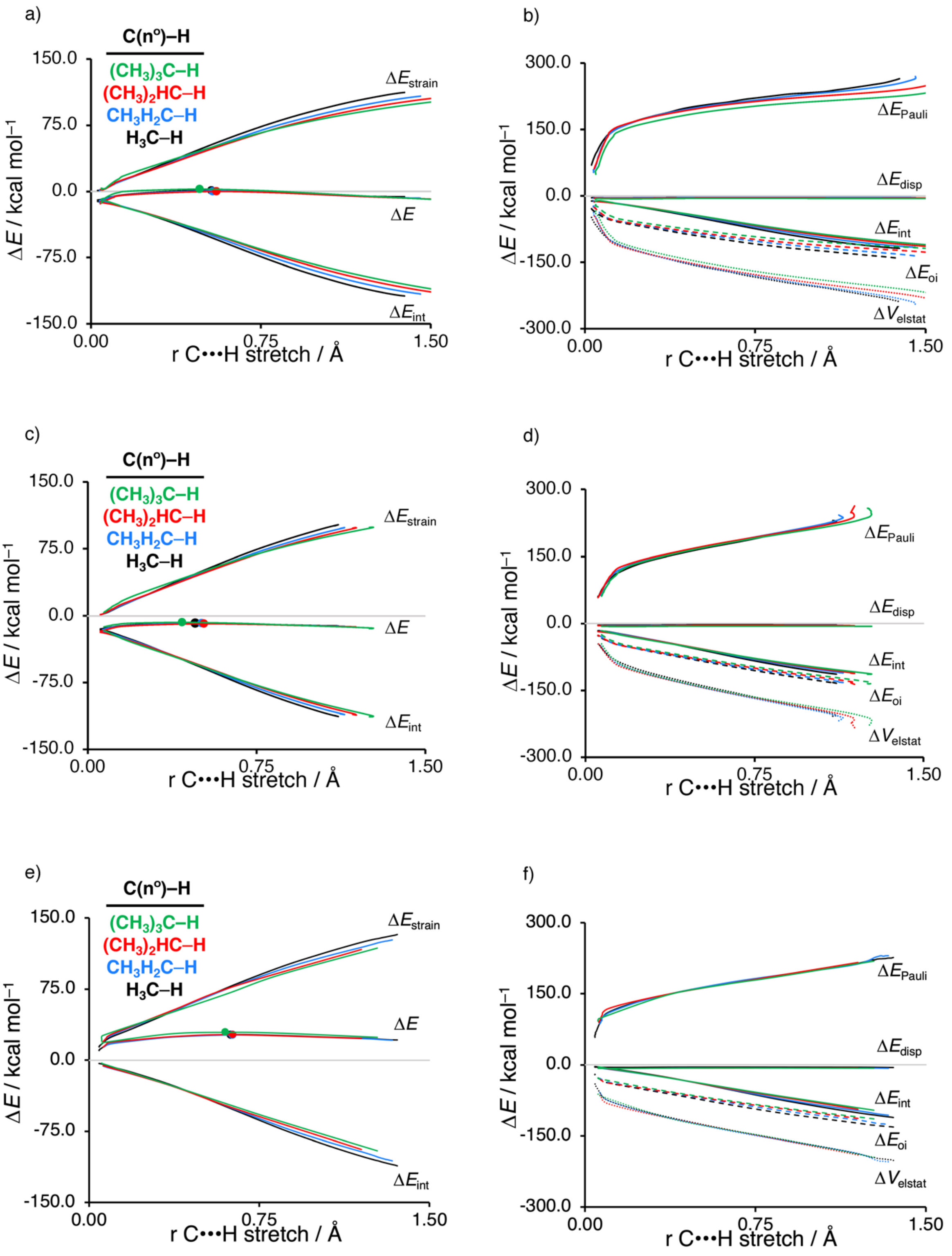

Figure S2. Activation strain analysis and energy decomposition analysis for the C–H activation of Pd (a,b), $PdCl^-$ (c,d) and $Pd(PH_3)_2$ (e,f) + C(nº)–H, along the IRC projected on the C•••H bond stretch. Computed at ZORA-BLYP-D3(BJ)/TZ2P.

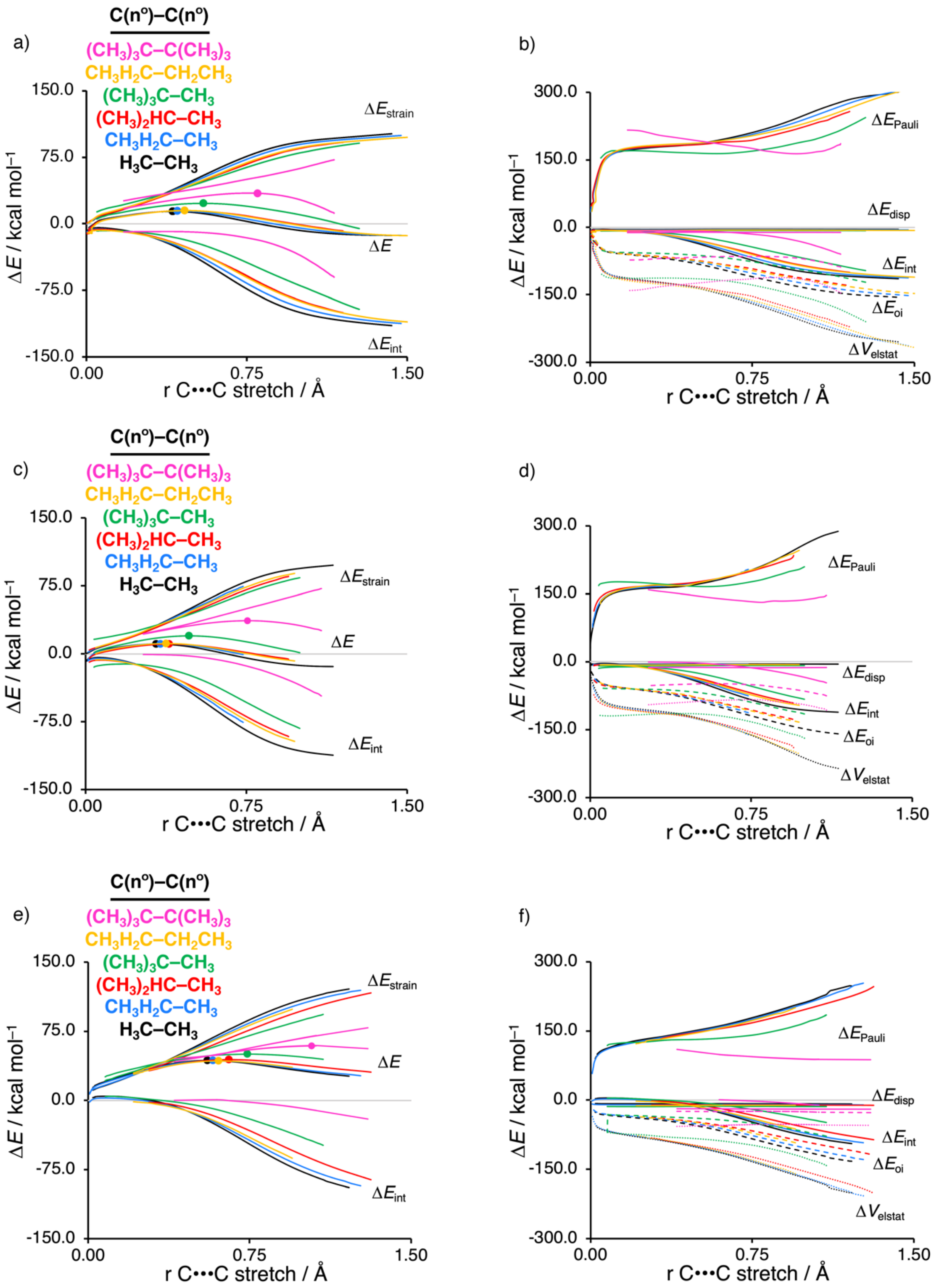

Figure S3. Activation strain analysis and energy decomposition analysis for the C–C activation of Pd (a,b), $PdCl^{-}$ (c,d) and $Pd(PH_3)_2$ (e,f) + C(nº)–C(mº), along the IRC projected on the C•••C bond stretch. Computed at ZORA-BLYP-D3(BJ)/TZ2P.

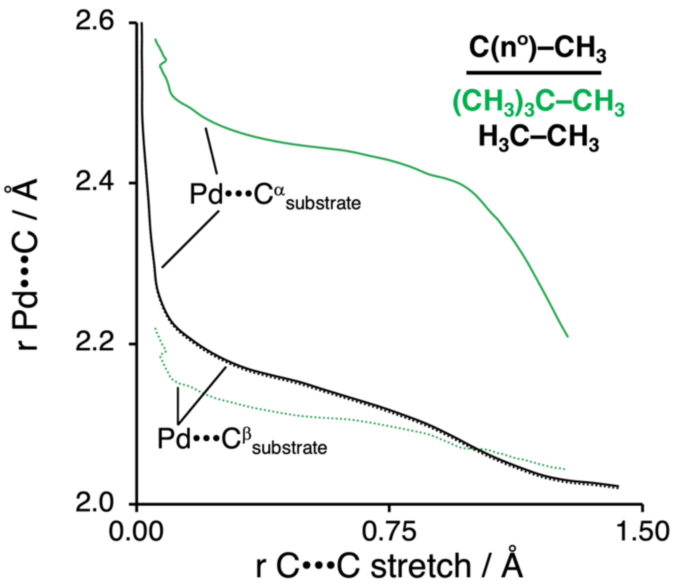

Figure S4. Pd•••$C^{\alpha}_{substrate}$ and Pd•••$C^{\beta}_{substrate}$ distance along the IRC projected onto the C•••C bond stretch for the Pd-induced C–C bond activation reactions of Pd + H_3C–CH_3 (black: methyl, 0°) and Pd + $(CH_3)_3C$–CH_3 (green: tertiary, 3°). Computed at ZORA-BLYP-D3(BJ)/TZ2P.

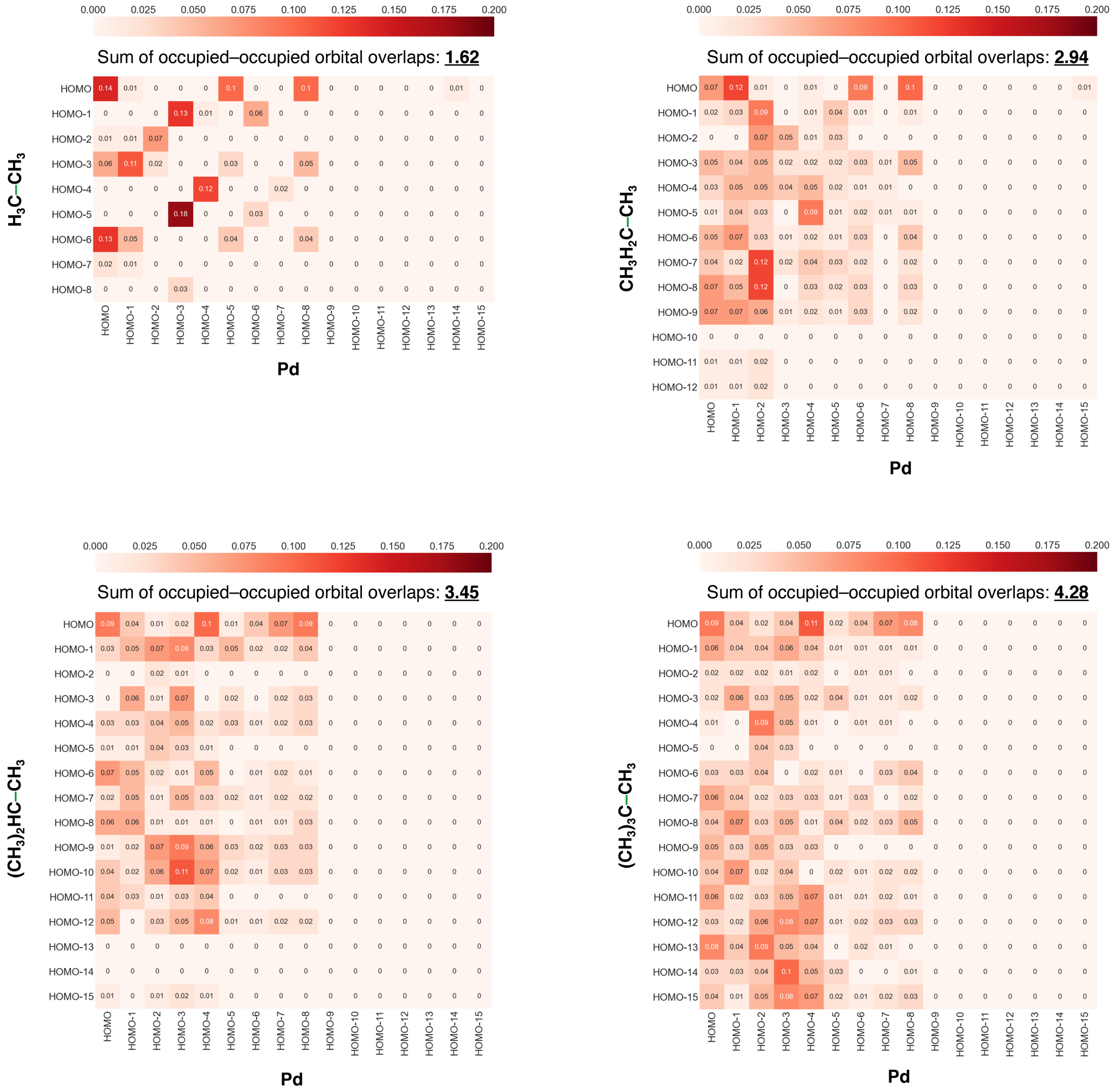

Figure S5. Heatmap of all occupied–occupied orbital overlaps from the HOMO to HOMO–15 at double consistent TS-like geometries (Δ*E**; see Table 3) for C–C bond activation by Pd obtained from the IRC at a C•••C bond stretch of 0.45 Å. The Pd•••$C^{\alpha}_{substrate}$ and Pd•••$C^{\beta}_{substrate}$ bond of $CH_3H_2C–CH_3$, $(CH_3)_2HC–CH_3$, and $(CH_3)_3C–CH_3$ were both set to 2.16 Å, respectively (Pd•••$C^{\alpha}_{substrate}$ and Pd•••$C^{\beta}_{substrate}$ distances in the consistent TS-like geometry for $H_3C–CH_3$). Computed at ZORA-BLYP-D3(BJ)/TZ2P.

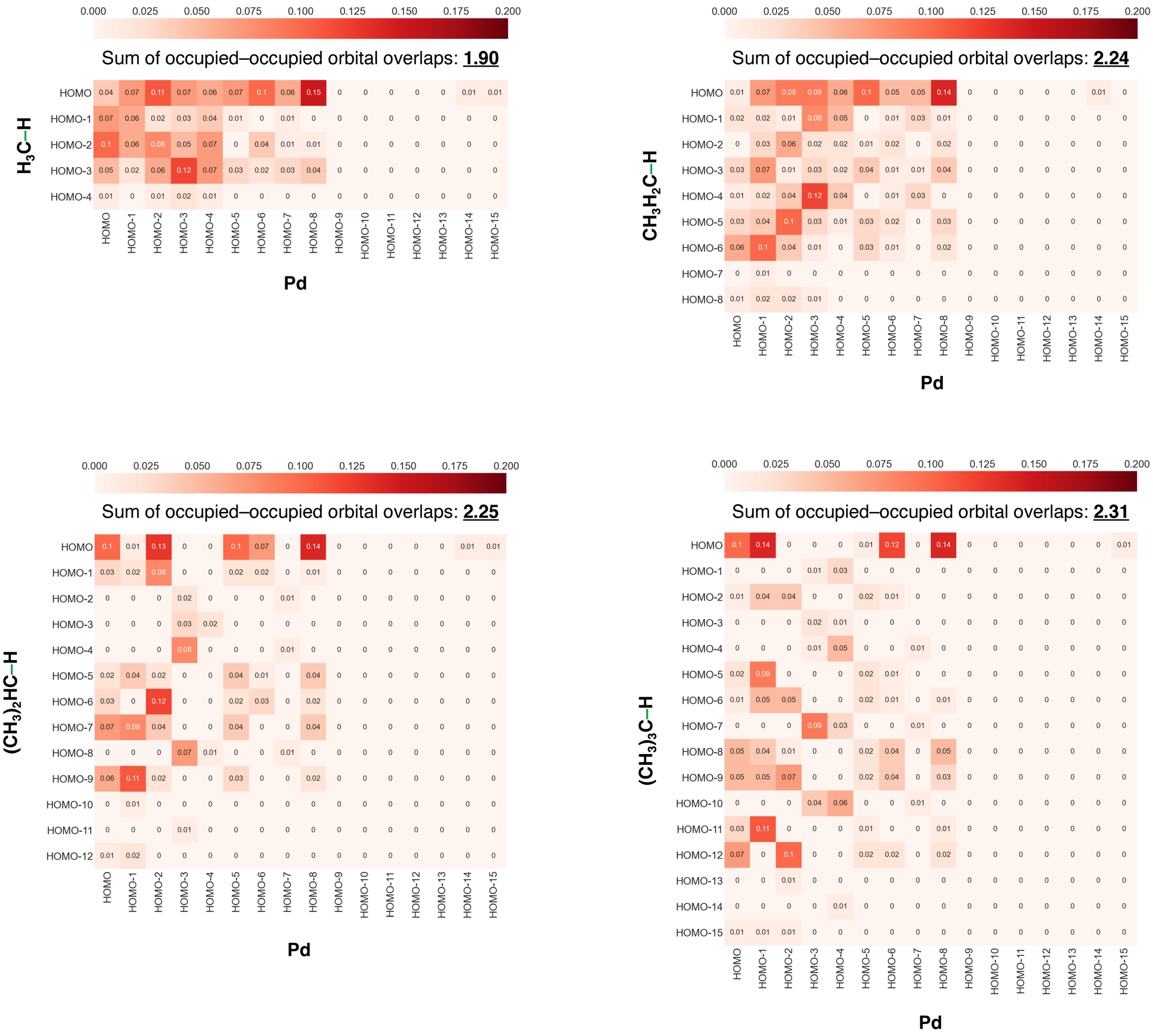

Figure S6. Heatmap of all occupied–occupied orbital overlaps from the HOMO to HOMO–15 at double consistent TS-like geometries (ΔE*; see Table 4) for C–H bond activation by Pd obtained from the IRC at a C•••H bond stretch of 0.54 Å. The Pd•••$C^{\alpha}_{substrate}$ and Pd•••$H^{\beta}_{substrate}$ bond of CH_3H_2C–H, $(CH_3)_2HC$–H, and $(CH_3)_3C$–H were both set to 2.11 and 1.63 Å, respectively (Pd•••$C^{\alpha}_{substrate}$ and Pd•••$H^{\beta}_{substrate}$ distances in the consistent TS-like geometry for H_3C–H). Computed at ZORA-BLYP-D3(BJ)/TZ2P.

Table S1. Activation strain and energy decomposition analyses (in kcal mol^{-1}) at double consistent TS-like geometries for the for the C–C bond activation of Pd + C(n°)–C(m°) with C(n°) and C(m°) = H_3C– (0°), CH_3H_2C– (1°), $(CH_3)_2HC$– (2°), $(CH_3)_3C$– (3°).[a]

**Substrate**	ΔE^*	ΔE_{strain}	ΔE_{int}	ΔV_{elstat}	ΔE_{Pauli}	ΔE_{oi}	ΔE_{disp}
$H_3C–CH_3$	13.5	32.3	–18.8	–123.4	178.9	–69.3	–5.0
$CH_3H_2C–CH_2CH_3$	13.9	31.3	–17.9	–134.4	194.0	–69.9	–7.1
$(CH_3)_2HC–CH(CH_3)_2$	18.1	28.7	–17.4	–144.5	215.4	–72.3	–9.2
$(CH_3)_3C–C(CH_3)_3$	65.2	49.5	15.7	–239.8	395.0	–127.0	–12.6

[a] Numerical experiment at double consistent TS-like geometries, ΔE^*, obtained from the IRC at a C•••C bond stretch of 0.45 Å. The Pd•••$C^{\alpha}_{substrate}$ and Pd•••$C^{\beta}_{substrate}$ bond of $CH_3H_2C–CH_2CH_3$, $(CH_3)_2HC–CH(CH_3)_2$, and $(CH_3)_3C–C(CH_3)_3$ were set to 2.16 and 2.16 Å, respectively (Pd•••$C^{\alpha}_{substrate}$ and Pd•••$C^{\beta}_{substrate}$ distances in the consistent TS-like geometry for $H_3C–CH_3$). Computed at ZORA-BLYP-D3(BJ)/TZ2P.

Table S2. Cartesian coordinates (in Å), energies (E, H and G, in kcal mol^{-1}), and number of imaginary vibrational frequencies (N_{imag}) of all stationary points of the C–C bond activation reactions in the gas phase, computed at ZORA-BLYP-D3(BJ)/TZ2P.

R: $H_3C–CH_3$
E = -900.45
H = -852.14
G = -869.47
N_{imag} = 0

C	0.00000000	0.00000000	0.76940200
H	0.00000000	1.02245100	1.16701300
H	0.88546800	-0.51122500	1.16701300
H	-0.88546800	-0.51122500	1.16701300
C	0.00000000	0.00000000	-0.76940200
H	-0.88546800	0.51122500	-1.16701300
H	0.88546800	0.51122500	-1.16701300
H	0.00000000	-1.02245100	-1.16000000

R: $CH_3H_2C–CH_3$
E = -1266.37
H = -1199.88
G = -1219.05
N_{imag} = 0

C	0.00000000	0.00000000	0.02092300
C	1.28119300	0.00000000	0.87211100
C	-1.28119300	0.00000000	0.87211100
H	0.00000000	0.87932900	-0.63860800
H	0.00000000	-0.87932900	-0.63860800
H	2.18105200	0.00000000	0.24506400
H	1.32251200	0.88587800	1.51943500
H	1.32251200	-0.88587800	1.51943500
H	-2.18105200	0.00000000	0.24506400
H	-1.32251200	-0.88587800	1.51943500
H	-1.32251200	0.88587800	1.51943500

R: $(CH_3)_2HC–CH_3$
E = -1633.44
H = -1549.08
G = -1570.66
N_{imag} = 0

C	0.00000000	0.00000000	0.36108300
C	1.46633300	0.00000000	-0.11124300
C	-0.73316700	-1.26988200	-0.11124300
C	-0.73316700	1.26988200	-0.11124300
H	0.00000000	0.00000000	1.46222100
H	2.00085200	0.88759000	0.24938700
H	2.00085200	-0.88759000	0.24938700
H	1.51825100	0.00000000	-1.20883300
H	-1.76910200	1.28899300	0.24938700
H	-0.23175000	2.17658400	0.24938700
H	-0.75912500	1.31484400	-1.20883300

H	-1.76910200	-1.28899300	0.24938700
H	-0.75912500	-1.31484400	-1.20883300
H	-0.23175000	-2.17658400	0.24938700

R: $(CH_3)_3C\text{–}CH_3$

E = -2000.88
H = -1898.79
G = -1920.66
N_{imag} = 0

C	0.00000000	0.00000000	0.00000000
C	-0.89231900	0.89231900	-0.89231900
C	-0.89231900	-0.89231900	0.89231900
C	0.89231900	-0.89231900	-0.89231900
C	0.89231900	0.89231900	0.89231900
H	-0.28233300	-1.53732300	1.53732300
H	-1.53732300	-0.28233300	1.53732300
H	-1.53732300	-1.53732300	0.28233300
H	-0.28233300	1.53732300	-1.53732300
H	-1.53732300	0.28233300	-1.53732300
H	-1.53732300	1.53732300	-0.28233300
H	1.53732300	0.28233300	1.53732300
H	1.53732300	1.53732300	0.28233300
H	0.28233300	1.53732300	1.53732300
H	1.53732300	-1.53732300	-0.28233300
H	0.28233300	-1.53732300	-1.53732300
H	1.53732300	-0.28233300	-1.53732300

R: $CH_3H_2C\text{–}CH_2CH_3$

E = -1632.28
H = -1547.61
G = -1569.46
N_{imag} = 0

C	0.00328000	0.01043000	0.00000000
C	1.54178200	-0.00585200	0.00000000
C	2.13245200	-1.42535800	0.00000000
C	-0.58737700	1.42994800	0.00000000
H	1.90687900	0.54461800	0.87977500
H	1.90687900	0.54461800	-0.87977500
H	3.22892700	-1.40408700	0.00000000
H	1.80780700	-1.98655400	0.88592500
H	1.80780700	-1.98655400	-0.88592500
H	-0.36182400	-0.54003700	-0.87977700
H	-0.36182400	-0.54003700	0.87977700
H	-1.68385800	1.40869900	0.00000000
H	-0.26271600	1.99113400	0.88592400
H	-0.26271600	1.99113400	-0.88592400

R: **$(CH_3)_2HC-CH(CH_3)_2$**

E = -2364.35
H = -2285.22
G = -2294.35
***N*$_{imag}$** = 0

C	0.77732500	0.04724400	0.05040100
H	-1.08186200	-0.21129100	-2.12745200
C	-0.77732500	-0.04724400	0.05040100
C	1.31801100	0.75602700	1.30963500
C	1.31799200	0.75600800	-1.20885100
H	2.40828500	0.85754900	1.25581100
C	-1.31799200	-0.75600800	-1.20885100
C	-1.31801100	-0.75602700	1.30963500
H	-0.89334600	-1.76580000	-1.29541200
H	0.89342500	1.76585300	1.39614700
H	1.08183500	0.21136300	2.22825600
H	0.89334600	1.76580000	-1.29541200
H	2.40825500	0.85759700	-1.15500900
H	1.08186200	0.21129100	-2.12745200
H	-2.40828500	-0.85754900	1.25581100
H	-0.89342500	-1.76585300	1.39614700
H	-1.08183500	-0.21136300	2.22825600
H	-2.40825500	-0.85759700	-1.15500900
H	1.16327700	-0.98589800	0.05040500
H	-1.16327700	0.98589800	0.05040500

R: **$(CH_3)_3C-C(CH_3)_3$**

E = -3094.98
H = -2938.42
G = -2966.90
***N*$_{imag}$** = 0

C	1.55633500	0.02908600	0.00005600
C	2.14620200	-1.39817700	-0.12528100
C	-0.03859000	-0.00249500	0.00010900
C	2.10574800	0.65095700	1.30850600
C	2.10134800	0.86772700	-1.18332200
C	-0.62851000	1.42475700	-0.12513300
C	-0.58365200	-0.84112600	-1.18325900
C	-0.58789900	-0.62444200	1.30856500
H	3.23699100	-1.35652300	-0.01771900
H	1.76558800	-2.07134700	0.65087900
H	1.92952800	-1.84576100	-1.10067600
H	-1.71929100	1.38305100	-0.01752400
H	-0.24788700	2.09791000	0.65104100
H	-0.41190300	1.87240300	-1.10051600
H	-1.67745200	-0.72900500	1.23745800
H	-0.17435200	-1.62141900	1.49659800
H	-0.37478900	0.00282300	2.18020900
H	-1.67689100	-0.75950000	-1.21960600
H	-0.19656100	-0.49377100	-2.14757800
H	-0.33721000	-1.90281900	-1.07961900

H	1.69191100	1.64778100	1.49685900
H	1.89303800	0.02342200	2.18008700
H	3.19527000	0.75588400	1.23715900
H	1.85456500	1.92934100	-1.07989700
H	3.19460600	0.78643900	-1.21945200
H	1.71455700	0.52010700	-2.14764500

R: PdCl-

E = -121.87
H = -119.13
G = -136.80
N_{imag} = 0

Pd	0.00000000	0.00000000	-0.01116000
Cl	0.00000000	0.00000000	2.32854700

R: $Pd(PH_3)_2$

E = -767.39
H = -730.11
G = -754.65
N_{imag} = 0

Pd	0.00000000	0.00000000	0.00000000
P	0.00000000	0.00000000	2.27833700
H	0.60992000	1.05641300	3.00983000
H	0.60992000	-1.05641300	3.00983000
H	-1.21984000	0.00000000	3.00983000
P	0.00000000	0.00000000	-2.27833700
H	-1.21984000	0.00000000	-3.00983000
H	0.60992000	-1.05641300	-3.00983000
H	0.60992000	1.05641300	-3.00983000

RC: Pd + $H_3C–CH_3$

E = -912.03
H = -862.48
G = -884.90
N_{imag} = 0

C	0.16296100	-2.75681400	0.00000000
C	0.91787600	-1.41867300	0.00000000
H	0.41628000	-3.34983700	-0.88710600
H	-0.91988700	-2.59524700	0.00000000
H	0.41628000	-3.34983700	0.88710600
H	2.00566500	-1.54237200	0.00000000
H	0.67830600	-0.84179700	-0.93125200
H	0.67830600	-0.84179700	0.93125200
Pd	-0.18138900	0.80477300	0.00000000

TS: Pd + $H_3C–CH_3$

E = -886.63
H = -839.64
G = -860.97
N_{imag} = 1, -493.99 cm^{-1}

Pd	0.00000000	0.00000000	-1.93398300

C -0.97151800 0.00110000 -0.00147700
C 0.97151800 -0.00110000 -0.00147700
H -1.74154500 0.24122800 -0.77986200
H 1.74154500 -0.24122800 -0.77986200
H -1.04005800 0.78285300 0.75262400
H -1.19478200 -0.97710900 0.42045600
H 1.04005800 -0.78285300 0.75262400
H 1.19478200 0.97710900 0.42045600

P: Pd + $H_3C–CH_3$
E = -913.32
H = -865.82
G = -888.77
N_{imag} = 0
Pd 0.00000000 0.00000000 -1.83304100
C -1.48544700 0.09008800 -0.46307200
C 1.48544700 -0.09008800 -0.46307200
H -2.39360900 0.17816300 -1.07682500
H 2.39360900 -0.17816300 -1.07682500
H -1.35312700 0.97119200 0.17062500
H -1.49673600 -0.83070500 0.12639200
H 1.35312700 -0.97119200 0.17062500
H 1.49673600 0.83070500 0.12639200

RC: Pd + $CH_3H_2C–CH_3$
E = -1278.44
H = -1210.64
G = -1235.25
N_{imag} = 0
C 0.00917800 -0.10111800 0.00836300
H 0.36857700 0.92920000 0.12312500
C -1.52757000 -0.09545000 -0.01690200
C 0.61451300 -0.72528000 -1.25897600
H 0.35159000 -0.65303800 0.89513900
H -1.95409500 -1.10288700 -0.07404600
H -1.93219300 0.36092200 0.92133100
H -1.88387800 0.43430000 -0.94226300
H 0.28221900 -1.76425800 -1.38550300
H 0.30907700 -0.16572200 -2.15310500
H 1.71010400 -0.72598200 -1.21981300
Pd -2.91852200 1.95651200 -0.02765000

TS: Pd + $CH_3H_2C–CH_3$
E = -1252.03
H = -1186.88
G = -1210.98
N_{imag} = 1, -477.168 cm^{-1}
C 0.03818800 -0.06538100 0.01885900
C 2.00355700 -0.01749800 0.10669000
H -0.03024800 -0.69635300 0.90434200
C -0.32336200 -0.80734700 -1.26665000

H	-0.70387400	0.76230600	0.18933100
H	2.78442300	0.76340600	-0.08945500
H	2.16962000	-0.42709500	1.10210000
H	2.12666500	-0.77761300	-0.66267400
H	-1.37385000	-1.13094800	-1.23522600
H	-0.19913000	-0.16896900	-2.14744300
H	0.29440600	-1.70290200	-1.40731400
Pd	1.00540700	1.89039300	-0.02235900

P: Pd + $CH_3H_2C–CH_3$

E = -1279.44
H = -1213.62
G = -1238.74
$\boldsymbol{N_{imag}}$ = 0

C	0.00262700	-0.03076100	-0.03336200
C	3.04411800	-0.12215300	0.07596900
H	0.04449300	0.42314500	-1.02998600
C	-0.00679200	1.00886100	1.08103300
H	-0.86494600	-0.71247900	0.02402100
H	3.92890600	-0.73304100	-0.15149600
H	2.91365800	0.66323700	-0.67337400
H	3.09656500	0.29330700	1.08706000
H	-0.90144100	1.64749700	0.99185200
H	-0.02622300	0.54796900	2.07443000
H	0.86864000	1.66531600	1.02626700
Pd	1.48249600	-1.41679600	0.01588600

RC: Pd + $(CH_3)_2HC–CH_3$

E = -1647.49
H = -1562.17
G = -1588.64
$\boldsymbol{N_{imag}}$ = 0

C	0.49010000	0.20777600	0.71300400
C	1.83391100	0.51440000	1.39009200
C	-0.21033700	1.50389700	0.26015000
H	0.69526200	-0.39991300	-0.17974600
C	-0.41644600	-0.61402400	1.64084300
H	2.33361400	-0.43574700	1.72937300
H	2.53480800	1.01375100	0.71197200
H	1.69873900	1.14511000	2.27698800
H	-1.15601400	1.28635500	-0.24999800
H	-0.43334300	2.14545200	1.12258900
H	0.42147000	2.07620800	-0.42929400
H	-1.33861000	-0.93288700	1.14244800
H	0.10767800	-1.55145300	1.98067300
H	-0.68480700	-0.04776600	2.54109600
Pd	1.89996200	-2.19893800	2.59294300

TS: Pd + $(CH_3)_2HC-CH_3$

E = -1618.69
H = -1535.56
G = -1561.26
***N*$_{imag}$** = 1, -455.552 cm^{-1}

C	0.01490400	-0.05053800	0.12466000
C	2.01354900	-0.14284100	0.09248600
C	-0.14748400	1.45481400	0.32987300
H	-0.68452400	-0.32971500	-0.71278500
C	-0.38309100	-0.87917200	1.34582600
H	2.76512700	-0.82348800	-0.37928100
H	2.29057300	0.89038600	-0.11146400
H	2.04208800	-0.35448200	1.16042700
H	-1.19587700	1.68560000	0.57230300
H	0.47200000	1.81953700	1.16000400
H	0.12075000	2.01502400	-0.57138000
H	-1.44875300	-0.72404900	1.57199100
H	-0.22696800	-1.94912700	1.17810900
H	0.18854700	-0.58454000	2.23607900
Pd	0.99746000	-1.08941000	-1.55284800

P: Pd + $(CH_3)_2HC-CH_3$

E = -1647.21
H = -1563.45
G = -1590.69
***N*$_{imag}$** = 0

C	-0.00281000	-0.00992800	0.00000000
C	3.10316100	-0.00414700	0.00000000
C	-0.08106000	0.82389700	-1.27443400
H	-0.78434500	-0.79670300	0.00000000
C	-0.08106000	0.82389700	1.27443400
H	4.01353500	-0.61986400	0.00000000
H	3.04995100	0.60972700	-0.90372200
H	3.04995100	0.60972700	0.90372200
H	-1.03338700	1.38163500	-1.29759000
H	0.72881000	1.56145600	-1.31358400
H	-0.02962800	0.20788900	-2.17729600
H	-1.03338700	1.38163500	1.29759000
H	-0.02962800	0.20788900	2.17729600
H	0.72881000	1.56145600	1.31358400
Pd	1.55448700	-1.32086600	0.00000000

RC: Pd + $(CH_3)_3C-CH_3$

E = -2015.99
H = -1913.25
G = -1941.51
***N*$_{imag}$** = 0

C	-0.04915100	-0.65609300	-0.12227200
C	1.48548400	-0.65156700	-0.25374500
C	-0.67938800	-0.18580200	-1.45008900
C	-0.53252000	-2.08726700	0.21238700

C	-0.46403400	0.30236000	1.00985600
H	-1.77441400	-0.17240900	-1.38419900
H	-0.33849000	0.83080800	-1.69264400
H	-0.39582800	-0.84793900	-2.27761800
H	1.97360600	-0.96952800	0.67409200
H	1.82735000	-1.29827200	-1.07113400
H	1.85289200	0.38589200	-0.49481800
H	-1.55400000	0.35631600	1.12063700
H	-0.02683800	0.00935900	1.97069800
H	-0.11607900	1.34940200	0.78142100
H	-1.62431600	-2.11758200	0.31321000
H	-0.24736300	-2.79115400	-0.57902100
H	-0.09449700	-2.43962600	1.15443300
Pd	1.49938200	2.28810200	0.04610800

TS: Pd + $(CH_3)_3C-CH_3$

E = -1977.76
H = -1876.95
G = -1904.67
N_{imag} = 1, -410.415 cm^{-1}

C	-0.22727600	-0.03767000	-0.07814100
C	1.86519200	-0.00860100	0.02714600
C	-0.93228600	1.30994500	-0.29779900
C	-0.20259900	-0.79685000	-1.41013600
C	-0.90829600	-0.91626600	0.98096400
H	-1.91713600	1.13342500	-0.76861400
H	-1.10512600	1.85944800	0.63616300
H	-0.35850700	1.95544400	-0.97050200
H	1.94608800	-1.09365100	-0.01465400
H	1.99004500	0.44532200	-0.95451100
H	2.71016000	0.36428300	0.68245400
H	-1.89224300	-1.24927100	0.60284600
H	-0.31756000	-1.81319800	1.19363000
H	-1.07577100	-0.38803800	1.92745500
H	-1.24533300	-0.97942500	-1.71692500
H	0.28043200	-0.22513500	-2.20923100
H	0.29312000	-1.76973900	-1.33111800
Pd	1.08659900	1.03687800	1.68367300

P: Pd + $(CH_3)_3C-CH_3$

E = -2015.91
H = -1914.62
G = -1944.30
N_{imag} = 0

C	0.88886600	1.24021200	0.00000000
C	4.17163300	1.04283300	0.00000000
C	0.83657900	2.08419200	-1.26751100
C	-0.04161500	0.01865300	0.00000000
C	0.83657900	2.08419200	1.26751100
H	5.00313800	0.32098000	0.00000000
H	4.22322200	1.66475700	-0.89969800

H	4.22322200	1.66475700	0.89969800
H	-0.11640700	2.64124100	-1.30550000
H	1.64789500	2.81810900	-1.28396500
H	0.90569000	1.47352800	-2.17270200
H	-0.66738300	-0.03228100	-0.89754100
H	-0.66738300	-0.03228100	0.89754100
H	0.53505100	-0.94801400	0.00000000
H	-0.11640700	2.64124100	1.30550000
H	0.90569000	1.47352800	2.17270200
H	1.64789500	2.81810900	1.28396500
Pd	2.44693500	-0.06116000	0.00000000

RC: Pd + $CH_3H_2C{-}CH_2CH_3$

E = -1647.18

H = -1561.89

G = -1588.46

N_{imag} = 0

C	0.40441500	0.79731700	0.97094300
C	1.83730500	0.27686900	0.80155700
C	-0.26628900	1.13057400	-0.36582600
C	2.50286000	-0.18247700	2.10403600
H	-0.19036600	0.04188800	1.50281300
H	0.41956800	1.69339800	1.60961600
H	2.45470500	1.03684200	0.30482100
H	1.80698900	-0.60804400	0.09117700
H	-1.32202600	1.40072300	-0.24993500
H	0.24746500	1.94618200	-0.88726600
H	-0.23729300	0.23139400	-1.04549100
H	3.51425900	-0.56365200	1.92205000
H	2.58038900	0.64797700	2.81864100
H	1.92113000	-0.98159800	2.58039200
Pd	1.06738800	-1.21089400	-1.62752800

TS: Pd + $CH_3H_2C{-}CH_2CH_3$

E = -1617.51

H = -1534.12

G = -1560.14

N_{imag} = 1, -456.45 cm^{-1}

C	-1.00040500	0.01238200	0.11627700
C	1.00040500	-0.01238200	0.11627700
C	-1.37140200	1.28280700	0.87783400
C	1.37140200	-1.28280700	0.87783400
H	-1.73989900	-0.14030100	-0.71778000
H	-1.07209900	-0.88574900	0.72887100
H	1.07209900	0.88574900	0.72887100
H	1.73989900	0.14030100	-0.71778000
H	-2.43240900	1.26106700	1.16560300
H	-0.78197100	1.39336100	1.79666700
H	-1.21339700	2.17695300	0.26551400
H	2.43240900	-1.26106700	1.16560300
H	0.78197100	-1.39336100	1.79666700

```
H   1.21339700  -2.17695300   0.26551400
Pd  0.00000000   0.00000000  -1.81310300
```

P: Pd + $CH_3H_2C–CH_2CH_3$

E = -1645.67
H = -1561.47
G = -1588.50
N_{imag} = 0

```
C  -0.05296500  -0.14710600  -0.11856900
C   3.05387700   0.00646900  -0.11977300
C  -0.11615700   1.27915400   0.41339100
C   3.07612300  -0.55706900   1.29585600
H  -0.88450000  -0.36725900  -0.81006000
H  -0.03668600  -0.89410500   0.68351700
H   3.01866600   1.10221100  -0.13494900
H   3.91366200  -0.34303500  -0.71702900
H  -1.03743100   1.42591300   1.00133800
H   0.72853700   1.50047300   1.07561000
H  -0.11685700   2.01835500  -0.39420700
H   3.97246700  -0.20383400   1.83254100
H   2.20388900  -0.23060900   1.87376300
H   3.09615200  -1.65195100   1.30091900
Pd  1.52612200  -0.60180700  -1.31555000
```

RC: Pd + $(CH_3)_2HC–CH(CH_3)_2$

E = -2382.15
H = -2261.24
G = -2291.94
N_{imag} = 0

```
C  0.61623700    2.74744100   0.99453700
H  0.83544800    4.05359500   2.72824500
C -0.93023500    2.62221400   1.02714300
C  1.22424900    3.09167700   2.36488200
C  1.12273300    3.72715500  -0.07736800
H  0.71467700    4.73324500   0.09518500
C -1.49809700    2.24096700  -0.35151200
C -1.39221600    1.58081900   2.06064400
H  1.00572000    2.32913700   3.11735700
H  2.31372900    3.17969100   2.28867500
H -1.33563500    3.60662800   1.31596400
Pd 0.46261400   -0.14840900  0.34210100
H  2.21562300    3.80044100  -0.04234200
H  0.84322600    3.41511700  -1.08737400
H  1.00415300    1.71565300   0.70960500
H -2.47686100    1.43401200   2.02138600
H -0.91561100    0.59394700   1.81805900
H -1.11315900    1.83623900   3.08646000
H -2.57899100    2.07048900  -0.30012500
H -1.30561100    2.99523700  -1.11963300
H -1.02340000    1.28751900  -0.68229400
```

TS: Pd + $(CH_3)_2HC–CH(CH_3)_2$

E = -2345.15

H = -2225.83

G = -2255.06

N

C	2.16625500	-0.00646800	-0.04008500
H	2.77829100	-0.93413400	-0.25078600
C	0.04576500	-0.01761100	0.03955200
C	2.50753700	0.36585700	1.39778000
C	2.60989800	1.05606400	-1.04725800
C	-0.26749500	1.41660200	-0.36978400
H	-0.59263200	-0.65773600	-0.64066800
C	-0.38507600	-0.33058700	1.47388700
H	3.70873200	1.12353100	-1.03859400
H	2.30253300	0.81259600	-2.06863300
H	2.22522500	2.05041000	-0.79915800
Pd	1.07778100	-1.68657100	-0.95738600
H	-1.34562300	1.59857700	-0.23987600
H	0.25994200	2.15091200	0.25182300
H	-0.02585000	1.61286800	-1.41783300
H	-1.48204400	-0.26727500	1.54200800
H	-0.09319800	-1.33844900	1.78369900
H	0.02313400	0.38393700	2.19574200
H	2.00620900	1.28745100	1.71898600
H	2.25653100	-0.42938200	2.10477100
H	3.59134900	0.54493700	1.47322500

P: Pd + $(CH_3)_2HC–CH(CH_3)_2$

E = -2381.04

H = -2261.01

G = -2291.71

N_{imag} = 0

C	-0.83601800	-0.12065800	0.13356500
C	2.44202900	-0.16639100	-0.22804000
H	-1.54640500	0.72649100	0.04188400
C	-0.98381500	-1.07126000	-1.04609900
C	-0.99033700	-0.80703400	1.48680000
C	2.53309000	-1.30813800	0.77386200
C	2.57966700	-0.62104600	-1.67702600
H	3.18947400	0.61820700	0.00856600
H	2.56258000	0.21804200	-2.37899100
H	1.77859800	-1.31566200	-1.95418100
Pd	0.83080700	1.06091200	0.0451290
H	-1.96982400	-1.31439700	1.54243500
H	-0.22296700	-1.57629400	1.63308600
H	-0.93030700	-0.10075700	2.32090600
H	-1.97223500	-1.56279400	-1.01907600
H	-0.88733300	-0.55790100	-2.00815800
H	-0.23063600	-1.86864200	-1.00460700
H	3.49818900	-1.83376500	0.66784300
H	2.44934700	-0.96118000	1.80826600

H	1.74491700	-2.05096700	0.59562700
H	3.53497900	-1.15838900	-1.8146760

RC: Pd + $(CH_3)_3C–C(CH_3)_3$

E = -3117.32
H = -2961.54
G = -2996.03
N_{imag} = 0

C	-0.90289900	-0.10348000	0.07463800
C	2.46323300	-0.28870300	-0.16881500
C	-1.87399000	1.07529200	-0.11354900
C	-0.99954900	-1.10992200	-1.06591800
C	-1.09063600	-0.77540900	1.43557000
C	2.42678000	-1.48266500	0.77790300
C	2.57835900	-0.73077100	-1.62813100
C	3.57031000	0.71170300	0.20752700
H	-2.91231200	0.72769300	0.03411600
H	-1.71157700	1.87298400	0.62611100
H	-1.81028700	1.50784400	-1.11794200
H	-2.08277000	-1.26225600	1.47593000
H	-0.33924500	-1.55117900	1.61126200
H	-1.03970100	-0.05631300	2.25993900
H	-2.00464100	-1.56957300	-1.07645700
H	-0.83433800	-0.64198100	-2.04180400
H	-0.27544900	-1.92285000	-0.94582900
H	3.36759100	-2.05677200	0.69432300
H	2.31297500	-1.17550700	1.82255500
H	1.60902300	-2.16725300	0.52846400
H	3.50415100	-1.31970000	-1.76596000
H	1.73965300	-1.36709500	-1.92612300
H	2.62193400	0.12193300	-2.31384600
H	4.55923500	0.26948100	-0.00941200
H	3.51441500	1.63825900	-0.38212400
H	3.55090600	0.96969300	1.27190500
Pd	0.85293400	0.98574600	0.06046600

TS: Pd + $(CH_3)_3C–C(CH_3)_3$

E = -3060.53
H = -2906.73
G = -2940.78
N_{imag} = 1, -244.37 cm^{-1}

C	2.25149900	-0.00731600	0.03949100
C	3.09902900	-1.30130400	0.06041800
C	-0.10988700	-0.00444700	-0.02546400
C	2.42320600	0.66377300	1.39272900
C	2.64687000	0.89911700	-1.11661100
C	-0.30436800	1.49740700	-0.15964000
C	-0.94883600	-0.67291100	-1.14227200
C	-0.50079000	-0.52625400	1.34923300
H	4.17177600	-1.02493400	0.03313700
H	2.94111400	-1.89060100	0.96833700

H	3.01857500	-1.98695400	-0.84410500
Pd	1.08630300	-1.88155300	-1.07060300
H	-1.38070600	1.71847900	-0.03972400
H	0.22542600	2.07844300	0.59820500
H	-0.01501000	1.86366000	-1.14931900
H	-1.58560500	-0.38108400	1.50148400
H	-0.29542500	-1.59714600	1.45400400
H	0.00909700	0.00536800	2.15622100
H	-2.02125800	-0.58824200	-0.87827900
H	-0.81581700	-0.18700000	-2.11314400
H	-0.84022800	-1.79945700	-1.27473800
H	1.87858700	1.60553300	1.48879500
H	2.13724800	0.00633300	2.21935700
H	3.49517700	0.89768300	1.52779900
H	2.12595400	1.85934300	-1.08824000
H	3.72885200	1.11687500	-1.05843800
H	2.45841700	0.42999000	-2.08842900

P: Pd + $(CH_3)_3C–C(CH_3)_3$

E = -3117.32

H = -2961.54

G = -2996.03

N_{imag} = 0

C	-0.90289900	-0.10348000	0.07463800
C	2.46323300	-0.28870300	-0.16881500
C	-1.87399000	1.07529200	-0.11354900
C	-0.99954900	-1.10992200	-1.06591800
C	-1.09063600	-0.77540900	1.43557000
C	2.42678000	-1.48266500	0.77790300
C	2.57835900	-0.73077100	-1.62813100
C	3.57031000	0.71170300	0.20752700
H	-2.91231200	0.72769300	0.03411600
H	-1.71157700	1.87298400	0.62611100
H	-1.81028700	1.50784400	-1.11794200
H	-2.08277000	-1.26225600	1.47593000
H	-0.33924500	-1.55117900	1.61126200
H	-1.03970100	-0.05631300	2.25993900
H	-2.00464100	-1.56957300	-1.07645700
H	-0.83433800	-0.64198100	-2.04180400
H	-0.27544900	-1.92285000	-0.94582900
H	3.36759100	-2.05677200	0.69432300
H	2.31297500	-1.17550700	1.82255500
H	1.60902300	-2.16725300	0.52846400
H	3.50415100	-1.31970000	-1.76596000
H	1.73965300	-1.36709500	-1.92612300
H	2.62193400	0.12193300	-2.31384600
H	4.55923500	0.26948100	-0.00941200
H	3.51441500	1.63825900	-0.38212400
H	3.55090600	0.96969300	1.27190500
Pd	0.85293400	0.98574600	0.06046600

RC: Pd(PH$_3$)$_2$ + H$_3$C–CH$_3$

E = -1671.02
H = -1584.84
G = -1618.29
***N*$_{imag}$** = 0

Pd	0.00000000	0.00000000	-0.50776300
C	0.09722500	0.76206000	3.10446500
C	-0.09722500	-0.76206000	3.10446500
H	0.62589600	1.08397700	2.19832600
H	-0.86766100	1.28393200	3.12121400
H	0.67230500	1.09573500	3.97713500
H	-0.62589600	-1.08397700	2.19832600
H	0.86766100	-1.28393200	3.12121400
H	-0.67230500	-1.09573500	3.97713500
P	-1.90150700	1.25680000	-0.51536000
P	1.90150700	-1.25680000	-0.51536000
H	2.17214600	-2.15982200	0.54945000
H	-2.18334600	2.17678100	-1.56250400
H	2.18334600	-2.17678100	-1.56250400
H	3.18479900	-0.64331600	-0.51384400
H	-3.18479900	0.64331600	-0.51384400
H	-2.17214600	2.15982200	0.54945000

TS: Pd(PH$_3$)$_2$ + H$_3$C–CH$_3$

E = -1624.40
H = -1540.07
G = -1572.77
***N*$_{imag}$** = 1, -449.315 cm^{-1}

Pd	0.00000000	0.00000000	-1.98411600
C	-0.01893100	1.04687600	-0.01010100
C	0.01893100	-1.04687600	-0.01010100
H	0.38404000	1.83224800	-0.67197400
H	-1.03258500	1.28429900	0.31059200
H	0.64944200	0.94983000	0.84347700
H	-0.38404000	-1.83224800	-0.67197400
H	1.03258500	-1.28429900	0.31059200
H	-0.64944200	-0.94983000	0.84347700
P	-1.61578400	1.10531300	-3.28784600
P	1.61578400	-1.10531300	-3.28784600
H	2.40871200	-2.20944900	-2.84330800
H	-1.36693300	1.67650900	-4.57190800
H	1.36693300	-1.67650900	-4.57190800
H	2.73100700	-0.33525100	-3.72822400
H	-2.73100700	0.33525100	-3.72822400
H	-2.40871200	2.20944900	-2.84330800

P: Pd(PH$_3$)$_2$ + H$_3$C−CH$_3$

E = -1650.23

H = -1565.11

G = -1598.01

***N*$_{imag}$** = 0

Pd	0.00000000	0.00000000	0.01427300
C	-1.02712300	0.98854100	1.60531800
C	1.02712300	-0.98854100	1.60531800
H	-1.52719000	1.91024600	1.28547000
H	-1.77482600	0.28306900	1.98385700
H	-0.30951900	1.22308300	2.39541400
H	1.52719000	-1.91024600	1.28547000
H	1.77482600	-0.28306900	1.98385700
H	0.30951900	-1.22308300	2.39541400
P	-1.28727300	1.25204800	-1.47490600
P	1.28727300	-1.25204800	-1.47490600
H	1.15487400	-2.66682900	-1.44886600
H	-1.28344700	1.11964000	-2.89341800
H	1.28344700	-1.11964000	-2.89341800
H	2.69872300	-1.22524700	-1.31620500
H	-2.69872300	1.22524700	-1.31620500
H	-1.15487400	2.66682900	-1.44886600

RC: Pd(PH$_3$)$_2$ + CH$_3$H$_2$C−CH$_3$

E = -2038.47

H = -1932.95

G = -1971.02

***N*$_{imag}$** = 0

Pd	-2.54114500	-0.49740700	-0.69413200
C	-1.41815500	0.50833000	-4.31397400
C	-2.45655300	-0.62439700	-4.29517200
H	-1.58633100	1.19453700	-3.47335600
H	-0.39591800	0.11743500	-4.23152300
H	-1.48052500	1.09162600	-5.24213800
H	-2.34812500	-1.18825100	-3.35569000
C	-3.89969200	-0.10339400	-4.39673200
H	-2.25586000	-1.32792100	-5.11708300
H	-5.31945200	-1.85070800	-1.66464500
H	0.50795600	-0.31689000	0.45112400
H	-5.48099700	-1.40228300	0.39528100
H	-5.70461400	0.12961900	-1.04359700
H	0.20114800	1.21894900	-0.96447400
H	0.54812200	-0.75765700	-1.61342400
P	-0.30101700	-0.08346900	-0.69426700
P	-4.78151000	-0.91152400	-0.74299600
H	-4.62660300	-0.92428000	-4.36045600
H	-4.11908700	0.58067500	-3.56579100
H	-4.06144100	0.44651000	-5.33355400

TS: Pd(PH$_3$)$_2$ + CH$_3$H$_2$C–CH$_3$
E = -1990.54
H = -1888.00
G = -1923.33
N_{imag} = 1, -426.46 cm^{-1}

Pd	1.05181400	1.96231500	0.01033800
C	2.13533000	0.00849600	0.07354900
C	0.01484400	-0.04652900	0.04297500
H	2.90794600	0.66038600	-0.36861700
H	2.39248300	-0.26075900	1.09810300
H	2.04629500	-0.87754900	-0.55202100
H	-0.70637400	0.66314400	0.48806200
C	-0.46737200	-0.60088300	-1.28942200
H	0.17168900	-0.83214100	0.78183000
H	-1.08090100	2.84176500	-2.47618500
H	2.73211200	4.56703100	1.35015400
H	-0.64555800	4.52589800	-1.32640100
H	0.78018300	3.78461500	-2.66489600
H	1.38717200	3.74825400	2.72494300
H	3.25266900	2.84370700	2.40756600
P	2.15102500	3.29010100	1.61249500
P	-0.02704400	3.27155600	-1.60883200
H	-1.34411800	-1.24993400	-1.13839300
H	-0.75436200	0.19445600	-1.98381000
H	0.30096800	-1.21113000	-1.78034100

P: Pd(PH$_3$)$_2$ + CH$_3$H$_2$C–CH$_3$
E = -2015.07
H = -1911.68
G = -1946.84
N_{imag} = 0

C	0.04921700	-0.09963800	0.02069300
C	2.91211600	-0.02748200	0.10296600
Pd	1.45057700	1.53014600	0.05011900
H	0.51770600	-0.93127200	0.55415100
H	-0.84263500	0.19827900	0.58907400
C	-0.32494500	-0.49742000	-1.41059600
H	2.87183000	-0.47270600	1.10302500
H	3.93172200	0.32467300	-0.09377300
H	2.64310300	-0.77996800	-0.64402800
P	-0.38257400	2.97200800	0.02410500
P	3.21082200	3.07334300	0.04954300
H	-1.11787700	3.14904700	1.22855400
H	-1.49793000	2.63025900	-0.78744200
H	-0.34248500	4.35112900	-0.33077000
H	3.09143500	4.49411400	0.06498400
H	4.17410800	2.98680500	1.09062800
H	4.13912700	3.00879100	-1.02457200
H	-1.04412700	-1.33308800	-1.41960000
H	-0.78772000	0.33123400	-1.96258100
H	0.55423000	-0.81785300	-1.98217800

RC: $Pd(PH_3)_2$ + $(CH_3)_2HC{-}CH_3$

E = -2406.03
H = -2283.23
G = -2321.92
N_{imag} = 0

Pd	2.07333000	0.18405900	0.79141300
C	3.22888500	-0.28178800	4.51232200
C	2.20464800	-1.29161300	3.96713400
H	3.01470000	0.72846600	4.14303600
H	4.24832600	-0.54638800	4.20385400
H	3.20867500	-0.25382700	5.61068000
H	2.26380200	-1.25114300	2.86428800
C	0.77148900	-0.89271800	4.35856800
C	2.53322200	-2.72539000	4.41526000
H	-1.10423500	0.49367400	1.36363700
H	5.06613600	1.00638000	-0.22933500
H	-0.84992700	-0.24328000	-0.59869100
H	-0.64990600	-1.54247100	1.05459200
H	5.20936100	-0.30468800	1.42100700
H	4.76503500	1.73426400	1.73058800
P	4.29864500	0.66451900	0.91791600
P	-0.15273200	-0.27636000	0.64025400
H	0.03705500	-1.59089000	3.93725200
H	0.53276900	0.11282000	3.99140300
H	0.64618400	-0.89399400	5.45034900
H	1.82287000	-3.44653700	3.99181600
H	2.48811900	-2.81424900	5.51000000
H	3.54155100	-3.01924500	4.09735900

TS: $Pd(PH_3)_2$ + $(CH_3)_2HC{-}CH_3$

E = -2356.56
H = -2236.15
G = -2273.40
N_{imag} = 1, -369.94 cm^{-1}

Pd	1.09596400	2.00318800	-0.01821000
C	2.10748700	0.02580600	0.04114400
C	-0.08848300	0.03534800	-0.05145300
H	2.87747700	0.63596800	0.54041400
H	2.41959700	-0.25564200	-0.96331800
H	1.92246100	-0.85366000	0.65360600
H	-0.74050900	0.87660700	-0.35090600
C	-0.53235300	-0.51704700	1.29474200
C	-0.11566100	-0.99283400	-1.17579700
H	-0.87707300	3.01067100	2.57154000
H	2.89572100	4.44393500	-1.48315100
H	-0.47969200	4.64871100	1.34682600
H	1.01497500	3.90642300	2.60561500
H	1.48924300	3.65134200	-2.80976700
H	3.30031900	2.65123400	-2.47212800
P	2.23629500	3.19389400	-1.68504500
P	0.12627100	3.38565500	1.62111000

H	-1.52960200	-0.97656700	1.19806000
H	-0.59154600	0.26208100	2.05974200
H	0.14367500	-1.30105900	1.65928000
H	-1.14808200	-1.35298000	-1.31204700
H	0.50751100	-1.86935600	-0.95914600
H	0.21360500	-0.56102000	-2.12661500

P: $Pd(PH_3)_2$ + $(CH_3)_2HC{-}CH_3$

E = -2381.39

H = -2259.93

G = -2296.53

N_{imag} = 0

C	0.11581200	-2.10490700	-0.47715300
C	2.16181000	-0.19604800	0.14925000
Pd	0.03532600	-0.00777100	0.04867500
H	1.17218900	-2.28667000	-0.69285800
C	-0.30001600	-2.95782600	0.72449700
C	-0.69590000	-2.43228300	-1.73458900
H	2.39316500	-1.03814700	0.80934200
H	2.66961800	0.70111200	0.52348200
H	2.52010100	-0.41381100	-0.86237000
P	-2.29817200	0.02945800	-0.10843300
P	0.32887100	2.24444100	0.66408300
H	-3.06460900	1.03056800	0.55211100
H	-2.91749200	0.18545600	-1.37931700
H	-3.08972000	-1.07321400	0.31197600
H	-0.69462300	3.22188000	0.84641600
H	1.00738900	2.50516500	1.88517500
H	1.16748000	3.05040700	-0.15212700
H	-0.54320500	-3.48589300	-2.02873500
H	-1.77756600	-2.30962100	-1.58227100
H	-0.40464700	-1.80661800	-2.58618400
H	-0.20195100	-4.03319900	0.49109200
H	-1.34721600	-2.78982500	1.00918800
H	0.32055400	-2.75235300	1.60395200

RC: $Pd(PH_3)_2$ + $(CH_3)_3C{-}CH_3$

E = -2773.78

H = -2633.26

G = -2672.98

N_{imag} = 0

C	2.43348400	-4.59204900	1.86597700
C	2.70071000	-3.46885500	2.89060500
C	3.34968600	-4.39554300	0.63826800
C	2.71871000	-5.96524400	2.51346100
C	0.95830900	-4.52988000	1.41186600
H	3.19410600	-5.19088200	-0.10157600
H	3.13648900	-3.43285800	0.15506200
H	4.40750400	-4.40711000	0.93104600
H	2.05246400	-3.57378600	3.76928400
H	3.74297400	-3.48946800	3.23268000

H	2.51089000	-2.48233400	2.44094000
H	0.73395000	-5.32621800	0.69152300
H	0.27796500	-4.64233700	2.26520200
H	0.74812700	-3.56776600	0.92960300
H	2.53438900	-6.78110700	1.80268800
H	3.76329500	-6.03366900	2.84359100
H	2.07659200	-6.12767800	3.38892900
Pd	2.17762600	-0.70919600	0.58377900
H	-0.81236500	0.56771700	0.74597100
H	5.11572400	0.65095100	0.13511400
H	-0.93375600	-1.38432400	-0.05361500
H	-0.69873900	-1.11251800	2.02429300
H	5.06562100	-1.24061100	-0.80338400
H	5.30927600	-1.10966300	1.28836200
P	4.43748300	-0.58866700	0.29285400
P	-0.08691500	-0.65310300	0.82447800

TS: $Pd(PH_3)_2$ + $(CH_3)_3C\text{–}CH_3$

E = -2718.04
H = -2580.45
G = -2618.37
N_{imag} = 1, -281.692 cm^{-1}

C	-1.45249800	-2.00775200	-0.03866900
C	0.75185100	-1.92509700	0.54358700
C	-2.21083200	-1.19881800	-1.09796100
C	-1.20268400	-3.41071500	-0.58136500
C	-2.18366400	-2.03915600	1.29725800
H	-3.17469900	-1.71023500	-1.28399700
H	-2.44386000	-0.17532600	-0.78704700
H	-1.67666700	-1.16451700	-2.05239500
H	0.42839200	-2.59442400	1.33626200
H	1.14969200	-2.46929500	-0.30985700
H	1.50514100	-1.22853600	0.95207100
H	-3.11400600	-2.62374100	1.18558100
H	-1.59046100	-2.52286600	2.08042000
H	-2.45675200	-1.03872400	1.64119500
H	-2.17919500	-3.87567800	-0.79767100
H	-0.63461100	-3.39169700	-1.51761100
H	-0.68284400	-4.06013100	0.12934200
Pd	-0.03165000	0.10397100	0.06600800
H	-1.92678400	1.33938900	2.55836500
H	2.12713000	1.83317000	-1.77469200
H	-1.31162900	2.91294300	1.34284400
H	0.04951900	2.02210100	2.65496400
H	0.41664700	1.43446500	-2.90640400
H	1.90027000	-0.02171000	-2.69891300
P	1.12682500	0.81803600	-1.83406200
P	-0.85562800	1.59314100	1.64134900

P: $Pd(PH_3)_2$ + $(CH_3)_3C{-}CH_3$

E = -2745.30

H = -2605.88

G = -2643.53

N_{imag} = 0

C	-1.67163800	-1.67078300	0.34183600
C	1.25955100	-1.81767000	-0.45202600
C	-3.05982900	-1.01090400	0.33084300
C	-1.68019900	-2.79863900	-0.69654200
C	-1.38296300	-2.21369800	1.74685900
H	-3.83357500	-1.77322100	0.53551000
H	-3.17042000	-0.24494300	1.10841000
H	-3.30056700	-0.56391000	-0.64087300
H	1.04164300	-2.60873400	0.26719900
H	1.08025900	-2.17571900	-1.47097900
H	2.31463900	-1.53534800	-0.35168200
H	-2.16033700	-2.94599600	2.03648000
H	-0.41630300	-2.72371800	1.80588200
H	-1.38970000	-1.41591600	2.49994200
H	-2.49693600	-3.50668500	-0.46409200
H	-1.86029600	-2.41425900	-1.70794400
H	-0.75117300	-3.37275600	-0.71162100
Pd	-0.07399600	-0.16197100	-0.11898600
H	-2.04496800	1.89658400	1.56674700
H	1.83076400	2.55712300	-0.83395600
H	-2.62457000	1.96650100	-0.46574000
H	-0.89132000	2.99577200	0.17721600
H	2.47246100	0.83058200	-1.89172500
H	2.95389300	1.05447800	0.15740800
P	1.80569800	1.13882800	-0.67618400
P	-1.45251700	1.69289900	0.29052100

RC: $Pd(PH_3)_2$ + $CH_3H_2C{-}CH_2CH_3$

E = -2405.22

H = -2282.03

G = -2319.84

N_{imag} = 0

Pd	2.32268500	-0.94852700	-0.24175800
C	2.85822000	-3.99223900	-2.27868900
C	1.91324000	-3.17866000	-3.17812000
H	2.63904300	-3.73889300	-1.23037000
C	4.34212700	-3.72197400	-2.56970400
H	2.64638600	-5.06560900	-2.39647300
H	2.10027400	-2.10946800	-2.99679600
C	0.43055600	-3.48938000	-2.92286500
H	2.15503500	-3.36777400	-4.23468800
H	-0.42904900	-0.38071700	-1.87839200
H	5.11484300	-0.51234100	1.34230900
H	-0.88514500	-1.47795100	-0.13563100
H	-0.51663900	0.59624300	-0.00634900
H	5.53576000	-0.79137000	-0.70849500

H	5.10359200	-2.45653200	0.51262200
P	4.53949200	-1.18265700	0.22801200
P	0.10271900	-0.55224700	-0.57087400
H	-0.22658300	-2.85668800	-3.53147000
H	0.17720900	-3.32457500	-1.86843100
H	0.19825400	-4.53647000	-3.15684900
H	4.99582400	-4.26930200	-1.87976900
H	4.60694800	-4.02229600	-3.59183100
H	4.56690600	-2.65227400	-2.47228900

TS: $Pd(PH_3)_2$ + $CH_3H_2C{-}CH_2CH_3$

E = -2356.74
H = -2235.94
G = -2273.30
N_{imag} = 1, -401.09 cm^{-1}

Pd	1.21292500	1.98031600	-0.01164100
C	2.19889500	-0.05441800	-0.04528300
C	0.05552900	0.03906900	0.00309200
H	2.94128400	0.61447900	0.42740800
C	2.66383000	-0.56291400	-1.40218400
H	2.02126500	-0.86686800	0.65842800
H	-0.62533700	0.77523400	-0.46248500
C	-0.45420600	-0.44107100	1.35416700
H	0.16248700	-0.77805900	-0.70948000
H	-0.76000800	3.00040400	2.55826500
H	2.90500700	4.53431700	-1.37389100
H	-0.29411400	4.64061000	1.35896100
H	1.15264500	3.84781300	2.64266300
H	1.45261100	3.80381500	-2.68752400
H	3.31008000	2.84992400	-2.53333400
P	2.27424500	3.28313400	-1.64573600
P	0.27209600	3.35961100	1.63385300
H	-1.34471800	-1.07627300	1.22630200
H	-0.72816600	0.39277800	2.00755900
H	0.29429800	-1.04567100	1.88258500
H	3.49346500	-1.27720200	-1.28231000
H	1.86405000	-1.09156700	-1.93689100
H	3.01203400	0.25014000	-2.04632200

P: $Pd(PH_3)_2$ + $CH_3H_2C{-}CH_2CH_3$

E = -2379.81
H = -2258.00
G = -2294.94
N_{imag} = 0

Pd	1.20609800	1.83281100	-0.01629700
C	2.39465100	0.18816800	-0.71760300
C	-0.12334400	0.29834400	0.68305200
H	3.45620100	0.46657800	-0.66017200
C	2.01801700	-0.20899200	-2.14915000
H	2.23738700	-0.64089200	-0.02100000
H	-1.15598300	0.67075900	0.63106500

C	0.22114500	-0.13820500	2.11131400
H	-0.04351000	-0.53804300	-0.01808900
H	-0.94717200	3.14244200	2.05125000
H	4.09828200	3.31564200	-0.06799200
H	-1.55810100	3.54413200	0.06681900
H	-0.11588100	4.78289100	0.99486700
H	2.77261800	4.65843400	-1.02493300
H	3.48936900	2.95136500	-2.06057600
P	2.88616700	3.25378700	-0.80945100
P	-0.34264500	3.39080900	0.78898100
H	-0.45679900	-0.93217300	2.46573800
H	0.14448600	0.69356100	2.82390500
H	1.24372500	-0.52937000	2.17715700
H	2.62344200	-1.05878000	-2.50521000
H	0.96477800	-0.50653900	-2.22048000
H	2.17176900	0.61596800	-2.85709600

RC: $Pd(PH_3)_2$ + $(CH_3)_2HC-CH(CH_3)_2$

E = -3138.20
H = -2979.54
G = -3019.01
N_{imag} = 0

C	0.77069500	3.55731800	-0.16308600
H	1.22247800	2.62746900	0.21560100
C	-0.70576900	3.52432900	0.33198700
C	1.59180100	4.73852100	0.38869500
C	0.85582200	3.50630700	-1.70033000
C	-1.52287400	4.77382800	-0.04823500
H	-1.16001400	2.65797600	-0.17390300
C	-0.79203400	3.25683000	1.84648300
H	2.60493300	-1.24438900	-1.73690400
H	1.71988700	0.29451100	-2.88269600
H	-1.73453200	-0.08876300	2.58130800
Pd	-0.01508200	-0.11317000	-0.17063000
H	-2.57072700	4.65129000	0.25391400
H	-1.13975800	5.67105700	0.45448400
H	-1.50988400	4.96003500	-1.12825100
H	-1.83311600	3.09818200	2.15569700
H	-0.21572300	2.36365600	2.11745600
H	-0.40384400	4.10143400	2.42948200
H	-2.64721400	-1.42950200	1.22661900
H	-2.93367100	0.65381100	1.01142300
H	2.92016600	0.77951400	-1.21722200
H	1.21229700	5.69839900	0.01633100
H	1.57843100	4.77199600	1.48400800
H	2.63945100	4.65572400	0.07293200
H	0.47472300	4.42808700	-2.15736100
H	1.89560400	3.38443400	-2.02984700
H	0.27167800	2.66578900	-2.09482200
P	1.82427700	-0.07691300	-1.51454900
P	-1.84986000	-0.25271600	1.17385500

TS: Pd(PH_3)$_2$ + (CH_3)$_2$HC–CH(CH_3)$_2$

E = -3084.50

H = -2927.99

G = -2969.76

N_{imag} = 1, -251.30 cm^{-1}

C	1.55222800	-1.70491100	0.61643700
H	1.42452700	-2.12172100	-1.49633800
H	4.78830600	-0.63039000	-2.71032000
H	2.21234900	-5.34179400	0.64009100
C	4.46422100	-1.53336500	-0.74825300
H	4.44148100	0.54530800	-1.42730400
Pd	3.23814600	-3.20941000	-1.75759000
H	0.84824000	0.25710400	-1.24914400
H	5.05454300	-2.44731400	-0.96189400
C	2.01990600	-1.48622700	-0.81088900
C	1.90737100	-0.05054800	-1.27933200
C	4.93392200	-0.40622100	-1.64998600
H	2.45576100	0.64723600	-0.63515400
H	2.25555000	0.07834900	-2.30842400
H	0.47714900	-1.46474000	0.68257400
H	1.67762500	-2.74087600	0.94308200
H	2.06171300	-1.05270200	1.33233200
H	4.04351000	-0.31848300	1.01773000
H	4.23879500	-2.05389900	1.35498400
H	5.64799100	-1.05121400	0.97555500
H	6.01407400	-0.24616200	-1.49112200
C	4.58678700	-1.22650200	0.72961700
H	3.93102700	-6.05258600	-0.31056500
H	2.05987900	-6.29026800	-1.20593300
H	2.57171600	-3.52429000	-4.95728200
H	4.12326100	-2.13269000	-4.83392500
P	2.82387800	-5.22072700	-0.64932800
H	4.53899900	-4.16614000	-4.67562700
P	3.64384500	-3.23983200	-4.06169800

P: Pd(PH_3)$_2$ + (CH_3)$_2$HC–CH(CH_3)$_2$

E = -3107.74

H = -2949.84

G = -2989.64

N_{imag} = 0

C	1.48642700	1.82818700	-0.74413700
H	2.41413700	1.24591800	-0.85571400
C	-1.38279300	1.77700400	0.77735500
C	1.81713700	2.96799800	0.21772200
C	1.07520800	2.31454400	-2.13719900
C	-1.73829100	2.93916200	-0.14818700
H	-2.29920500	1.17502900	0.87510300
C	-0.97500800	2.22701900	2.18362900
H	2.15264500	-0.99496600	-2.17203500
H	1.40586500	-2.63043400	-1.03964400

H	-1.25932600	-2.69065000	0.87055400
Pd	0.06370000	0.30147000	-0.01235000
H	-2.56591900	3.52972100	0.28381300
H	-0.90318200	3.62894600	-0.29955600
H	-2.06484800	2.58825900	-1.13414700
H	-1.75139400	2.87816200	2.62453300
H	-0.83792500	1.37468300	2.86060300
H	-0.04125700	2.79749900	2.17988000
H	-1.99089800	-1.12056600	2.10066700
H	2.88241400	-1.35201900	-0.21885900
H	-2.74488700	-1.36878000	0.14006900
H	0.96902300	3.63782200	0.38614800
H	2.14689300	2.59354300	1.19378400
H	2.63527800	3.58595100	-0.19331600
H	0.12879300	2.86376700	-2.11993600
H	1.83879800	2.99684400	-2.55242100
H	0.96053700	1.48212900	-2.84259900
P	1.62460300	-1.23115300	-0.87319200
P	-1.47937500	-1.28472600	0.78437000

RC: $Pd(PH_3)_2$ + $(CH_3)_3C–C(CH_3)_3$

E = -3868.97

H = -3674.32

G = -3718.49

N_{imag} = 0

C	0.85612900	3.69924900	-0.04592600
C	1.48740600	2.51092300	0.71880900
C	-0.72665600	3.75338500	0.16192000
C	1.53221500	4.99660100	0.46684200
C	1.21455000	3.53793500	-1.54443300
C	-1.30449200	5.11670800	-0.29643500
C	-1.44185800	2.64679600	-0.64985200
C	-1.09796300	3.55784000	1.65310000
H	2.54838500	2.42147500	0.45463500
H	1.42792700	2.64413600	1.80388600
H	1.00745500	1.55121900	0.46893800
Pd	-0.07126600	-0.56399000	-0.04108100
H	-2.40019000	5.08208500	-0.26277000
H	-0.98032900	5.93867300	0.35003900
H	-1.01500300	5.35769900	-1.32546900
H	-2.17524200	3.71439500	1.78934900
H	-0.86468200	2.54509700	1.99674300
H	-0.57576700	4.26754100	2.30495400
H	-2.50714300	2.62454800	-0.38779300
H	-1.37172600	2.82037100	-1.72895200
H	-1.03342400	1.64546600	-0.44118600
H	1.26836500	5.86593800	-0.14429800
H	1.26154100	5.21529100	1.50621600
H	2.62281900	4.88371300	0.42751200
H	0.74658200	4.30957100	-2.16591700
H	2.30055700	3.62062900	-1.67580700

H	0.90861800	2.55984400	-1.92894000
P	0.88563100	-0.66541600	-2.11122600
P	-1.11626100	-0.72291000	1.98257600
H	-2.35707300	-0.06249400	2.20176400
H	2.18919300	-0.14115000	-2.33469100
H	-1.51550600	-1.97220500	2.53116300
H	-0.48834000	-0.24031800	3.16575700
H	1.10461800	-1.89367100	-2.79351200
H	0.27522800	0.00382500	-3.20811600

TS: $Pd(PH_3)_2$ + $(CH_3)_3C{-}C(CH_3)_3$

E = -3802.99

H = -3611.83

G = -3654.06

N_{imag} = 1, -175.07 cm^{-1}

C	1.32480200	2.33038200	0.06558900
C	2.33040800	1.33293300	0.65242500
C	-1.27283900	2.25584400	-0.05332500
C	1.31515900	3.54488200	0.97354200
C	1.65150300	2.64221500	-1.38189900
C	-1.33342400	3.51308200	-0.90054400
C	-2.20147700	1.22624300	-0.70766700
C	-1.63856700	2.48539800	1.40080000
H	3.32213500	1.83053200	0.64468000
H	2.11428000	1.07824300	1.69329100
H	2.43811600	0.40943000	0.07280100
Pd	0.08382900	-0.11421900	0.01115800
H	-2.37716300	3.87571200	-0.88524000
H	-0.71312600	4.33705400	-0.54726900
H	-1.08934400	3.31015500	-1.94780800
H	-2.67214700	2.87685500	1.44841400
H	-1.61384700	1.55776500	1.97790900
H	-1.00121400	3.22018400	1.89474800
H	-3.22478000	1.65347400	-0.68222000
H	-1.95733600	1.05130200	-1.75889700
H	-2.24936000	0.26240000	-0.18690400
H	0.63725100	4.34245000	0.66898100
H	1.09690100	3.27289800	2.01118300
H	2.33254300	3.97653400	0.96860200
H	0.99048800	3.38841400	-1.82417200
H	2.67665100	3.05489100	-1.43202100
H	1.63172400	1.74375900	-2.00447700
P	0.41826600	-1.08590700	-2.04655300
P	-0.18467400	-1.10071300	2.06511200
H	-1.33334800	-0.91625900	2.89825200
H	0.81236700	-2.45090300	-2.17194400
H	-0.17956700	-2.51773800	2.22895100
H	0.74095200	-0.87585100	3.13174200
H	-0.66992100	-1.20877100	-2.96332300
H	1.33605800	-0.63776800	-3.05181600

P: $Pd(PH_3)_2$ + $(CH_3)_3C{-}C(CH_3)_3$

E = -3835.20
H = -3641.79
G = -3684.76
***N*imag** = 0

C	1.67274100	1.84260000	-0.37093400
C	2.99053100	1.07837300	-0.13462300
C	-1.59706400	1.75156900	0.38956900
C	1.61687500	2.96796500	0.66535200
C	1.63718800	2.38888800	-1.79915500
C	-1.60095500	2.88760800	-0.63641900
C	-2.87318700	0.92155300	0.14665000
C	-1.58872400	2.28521400	1.82293700
H	3.83365100	1.79292400	-0.13126000
H	3.00274800	0.55815300	0.83030200
H	3.21014800	0.34921700	-0.92283200
Pd	0.07682600	0.30778100	0.00123900
H	-2.51588000	3.49497400	-0.50736600
H	-0.75430400	3.56698700	-0.53611600
H	-1.60942300	2.50333900	-1.66310300
H	-2.46825600	2.93520300	1.99141000
H	-1.64138000	1.47440700	2.55942100
H	-0.69903000	2.88047600	2.04191900
H	-3.75265300	1.59082400	0.15086000
H	-2.85917200	0.41084600	-0.82329700
H	-3.05313100	0.17429100	0.92789300
H	0.73579800	3.60295800	0.57072200
H	1.64550600	2.57546900	1.68855000
H	2.49865500	3.62358800	0.54210700
H	0.71736200	2.93835800	-2.01341900
H	2.48119200	3.08608700	-1.96025200
H	1.73328300	1.58934200	-2.54350700
P	1.25164900	-1.34391300	-1.28229500
P	-1.00841500	-1.42036300	1.26163600
H	-1.61832800	-1.09159100	2.50348300
H	2.29744600	-2.24495300	-0.90999000
H	-2.00378000	-2.37092900	0.87491300
H	-0.10937700	-2.38911200	1.79006800
H	0.40574900	-2.35196900	-1.82474900
H	1.84171200	-0.96426700	-2.51921400

RC: $PdCl^-$ + $H_3C{-}CH_3$

E = -1038.43
H = -987.97
G = -1014.06
***N*imag** = 0

C	-0.03093500	-1.53404200	0.00000000
C	-0.01385700	0.00242900	0.00000000
Pd	1.93855600	1.34205700	0.00000000
H	-0.54363300	-1.93323800	0.88843700
H	0.99220100	-1.92486800	0.00000000

H -0.54363300 -1.93323800 -0.88843700
H -1.02274900   0.43118500   0.00000000
H   0.50367900   0.38716800   0.92898900
H   0.50367900   0.38716800 -0.92898900
Cl 3.89279200   2.64577900   0.00000000

TS: $PdCl^-$ + $H_3C–CH_3$
E = -1011.42
H = -963.07
G = -987.94
N_{imag} = 1, -498.09 cm^{-1}
Cl 0.00000000   0.00000000 -2.34013800
Pd 0.00000000   0.00000000   0.06029500
C   0.93161000   0.07223800   2.04277400
C -0.93161000 -0.07223800   2.04277400
H   1.65793000   0.38149800   1.24493800
H -1.65793000 -0.38149800   1.24493800
H   1.25858700 -0.88373000   2.45925000
H -1.25858700   0.88373000   2.45925000
H   0.97215600   0.84500900   2.81575900
H -0.97215600 -0.84500900   2.81575900

P: $PdCl^-$ + $H_3C–CH_3$
E = -1036.11
H = -987.81
G = -1013.07
N_{imag} = 0
Cl 0.00000000   0.00000000 -4.34997800
Pd 0.00000000   0.00000000 -1.90558600
C   1.34887900   0.00521000 -0.35963900
C -1.34887900 -0.00521000 -0.35963900
H   2.30935600   0.22770900 -0.84666500
H -2.30935600 -0.22770900 -0.84666500
H   1.38846100 -0.98694700   0.11071100
H -1.38846100   0.98694700   0.11071100
H   1.13452700   0.76303000   0.40532600
H -1.13452700 -0.76303000   0.40532600

RC: $PdCl^-$ + $CH_3H_2C–CH_3$
E = -1404.87
H = -1336.34
G = -1364.70
N_{imag} = 0
C   1.51934300   0.02187500 -0.05128800
H   1.86406100   1.06402000 -0.05673100
C -0.01825100   0.00826800   0.00001800
C   2.09469800 -0.71468000 -1.27870600
H   1.92617600 -0.43663800   0.86579900
H -0.41574800 -1.01399900   0.04670500
H -0.38996700   0.54833000   0.92201400
H -0.44833000   0.48695100 -0.93056200

```
H   1.77182900  -1.76566900  -1.29634300
H   1.74816000  -0.24707100  -2.21012400
H   3.19458600  -0.70233000  -1.28141600
Pd -1.48707500   1.88913300  -0.00837200
Cl -2.92718300   3.75031000  -0.01649600
```

TS: $PdCl^-$ + $CH_3H_2C–CH_3$

E = -1377.16
H = -1310.69
G = -1338.27
N_{imag} = 1, -486.19 cm^{-1}

```
C  -0.95556400  -1.99931800   0.01144300
C   0.93699800  -1.97429200   0.01114700
H  -0.99759100  -2.73455100   0.82144200
C  -1.44325600  -2.60193600  -1.30895200
H  -1.65639800  -1.17821400   0.32171700
H   1.68743200  -1.21615400  -0.32423600
H   1.20101400  -2.31518800   1.01646500
H   1.01247800  -2.81012100  -0.69146200
H  -2.46318700  -3.00792600  -1.20387600
H  -1.46457700  -1.84140900  -2.09666900
H  -0.79590900  -3.42388400  -1.65290100
Pd -0.02036200  -0.00627000  -0.01148100
Cl  0.01742300   2.39276300  -0.07193700
```

P: $PdCl^-$ + $CH_3H_2C–CH_3$

E = -1402.68
H = -1335.52
G = -1364.55
N_{imag} = 0

```
C  -1.12396400   1.73510500   0.35084500
C   1.35366800   1.59433400  -0.84109700
H  -0.66691700   2.69923700   0.61164100
C  -2.23186200   1.35418500   1.33805300
H  -1.50628800   1.80005000  -0.67744300
H   2.25969700   1.05503300  -1.16312600
H   0.78307000   1.89640400  -1.73166900
H   1.65354400   2.49799900  -0.28952000
H  -3.04350100   2.10862100   1.32798700
H  -2.66912500   0.38011600   1.09277900
H  -1.84899500   1.28397800   2.36263700
Pd  0.31409800   0.28253000   0.36945100
Cl -0.13182600  -1.74599200   1.65835900
```

RC: $PdCl^-$ + $(CH_3)_2HC–CH_3$

E = -1765.99
H = -1681.00
G = -1707.44
N_{imag} = 0

```
C   0.68387502   0.14423807   3.42525640
C   2.15020802   0.14423807   2.95293040
```

```
C  -0.04929198  -1.12564393   2.95293040
C  -0.04929198   1.41412007   2.95293040
H   0.68387502   0.14423807   4.52639440
H   2.68472702   1.03182807   3.31356040
H   2.68472702  -0.74335193   3.31356040
H   2.20212602   0.14423807   1.85534040
H  -1.08522698   1.43323107   3.31356040
H   0.45212502   2.32082207   3.31356040
H  -0.07524998   1.45908207   1.85534040
H  -1.08522698  -1.14475493   3.31356040
H  -0.07524998  -1.17060593   1.85534040
H   0.45212502  -2.03234593   3.31356040
Pd  1.12322575  -0.04915043  -0.30743724
Cl  1.25302999   0.15467558  -2.63463183
```

TS: $PdCl^-$ + $(CH_3)_2HC{-}CH_3$

E = -1744.36

H = -1660.02

G = -1689.31

N_{imag} = 1, -465.58 cm^{-1}

```
C  -1.03288200   1.66309600   1.16028500
C   0.89287400   1.71391000   0.97339100
C  -1.37504600   2.99942100   0.49924700
H  -1.71836300   0.90139000   0.69823500
C  -1.30530800   1.67233700   2.66516800
H   1.68085400   0.93144700   1.04535700
H   1.08596900   2.34898300   0.10506400
H   0.97524300   2.31460700   1.88595100
H  -2.42797600   3.26548600   0.69463700
H  -0.75209700   3.82352900   0.88449700
H  -1.23568700   2.94612800  -0.58626900
H  -2.36322800   1.91446800   2.86544300
H  -1.09114400   0.69338000   3.10578300
H  -0.69319000   2.42546200   3.18825100
Pd -0.05227300  -0.13404900   0.30284700
Cl  0.10645500  -2.32789400  -0.64888500
```

P: $PdCl^-$ + $(CH_3)_2HC{-}CH_3$

E = -1768.25

H = -1683.25

G = -1713.84

N_{imag} = 0

```
C  -2.09162700  -2.07173500   0.02695700
C  -0.07653600  -0.01992900   0.11797400
C  -2.75107500  -1.63803000  -1.28795100
H  -2.20921300  -3.16872200   0.13307200
C  -2.75790000  -1.39742000   1.23086600
H  -0.89495200   0.42148200  -0.46157900
H   0.89296400   0.32775500  -0.25976500
H  -0.18646000   0.23636100   1.17986700
H  -3.84522500  -1.82599500  -1.27813900
```

H	-2.61495600	-0.56252900	-1.46746700
H	-2.32551500	-2.17046400	-2.14718800
H	-3.85233700	-1.57904300	1.24737200
H	-2.34099100	-1.76425200	2.17673200
H	-2.61606100	-0.30832800	1.20793100
Pd	-0.01177600	-2.06001400	-0.01769900
Cl	2.26565800	-2.93513800	-0.12458200

RC: $PdCl^-$ + $(CH_3)_3C-CH_3$

E = -2140.93

H = -2037.36

G = -2067.61

N_{imag} = 0

C	1.53001300	-0.03998500	0.00000000
C	2.07417700	1.40416000	0.00000000
C	-0.01575200	-0.02450800	0.00000000
C	2.03440000	-0.77985300	-1.26124200
C	2.03440000	-0.77985300	1.26124200
H	-0.42373900	-1.04260400	0.00000000
H	-0.40550400	0.48807200	0.93029500
H	-0.40550400	0.48807200	-0.93029500
H	3.17486100	1.40589100	0.00000000
H	1.71510000	1.95166600	-0.87877900
H	1.71510000	1.95166600	0.87877900
H	1.65676600	-1.81135500	1.28748000
H	3.13324400	-0.81861000	1.28804900
H	1.68746700	-0.26997100	2.16862100
H	1.65676600	-1.81135500	-1.28748000
H	1.68746700	-0.26997100	-2.16862100
H	3.13324400	-0.81861000	-1.28804900
Pd	-1.43464400	1.86037400	0.00000000
Cl	-2.84616400	3.74207200	0.00000000

TS: $PdCl^-$ + $(CH_3)_3C-CH_3$

E = -2102.83

H = -2000.69

G = -2031.96

N_{imag} = 1, -452.47 cm^{-1}

C	-1.41555200	-1.97716400	0.00000000
C	0.61148200	-2.08644800	0.00000000
C	-2.09344200	-1.45408200	-1.27926700
C	-1.56450300	-3.51359700	0.00000000
C	-2.09344200	-1.45408200	1.27926700
H	-3.10606300	-1.90373900	-1.37370000
H	-2.19222900	-0.36501200	-1.27987300
H	-1.52325100	-1.73959800	-2.17081300
H	0.68809900	-2.70243100	0.89911400
H	0.68809900	-2.70243100	-0.89911400
H	1.48475100	-1.36388000	0.00000000
H	-3.10606300	-1.90373900	1.37370000
H	-1.52325100	-1.73959800	2.17081300

```
H  -2.19222900  -0.36501200   1.27987300
H  -2.64147400  -3.76011600   0.00000000
H  -1.11834600  -3.97639800  -0.88824700
H  -1.11834600  -3.97639800   0.88824700
Pd  0.03837600  -0.02904200   0.00000000
Cl -0.20691500   2.36366600   0.00000000
```

P: PdCl⁻ + $(CH_3)_3C$–CH_3

E = -2137.02
H = -2034.26
G = -2066.57
N_{imag} = 0

```
Pd -2.05984800   0.00572500  -0.03079300
C  -2.11761100   2.10179200  -0.01314900
C  -0.02218600   0.05731900   0.16383200
C  -1.59348400   2.70644500  -1.32243200
C  -3.63687600   2.32308600   0.08684200
C  -1.41959700   2.75690800   1.18392100
H   0.44387300   0.88221900  -0.38668600
H   0.33038800  -0.90787900  -0.21989900
H   0.20252900   0.14565500   1.23505300
H  -1.76853700   3.80403400  -1.35076000
H  -0.51523300   2.54500200  -1.43930800
H  -2.09210900   2.26661700  -2.19490400
H  -4.17461800   1.83542900  -0.73827200
H  -4.04144700   1.93599000   1.03092400
H  -3.88370300   3.40535500   0.03960100
H  -1.59309100   3.85431800   1.19295500
H  -1.79233800   2.35223800   2.13303100
H  -0.33519600   2.59890400   1.15597300
Cl -2.86621600  -2.29365700  -0.13302800
```

RC: PdCl⁻ + CH_3H_2C–CH_2CH_3

E = -1772.32
H = -1686.20
G = -1715.35
N_{imag} = 0

```
C  -1.54108500  -0.05255400  -0.00009500
C  -0.00487800   0.02167300   0.00022200
C   0.67067900  -1.35562800   0.00017000
C  -2.22180100   1.32441900   0.00023200
H   0.32577100   0.58883400   0.92228500
H   0.32607500   0.58898900  -0.92173100
H   1.76007100  -1.24828900   0.00037000
H   0.38376300  -1.94051200   0.88867700
H   0.38407900  -1.94029900  -0.88857500
H  -1.87349800  -0.62803300  -0.88137800
H  -1.87382900  -0.62861800   0.88067800
H  -3.31750100   1.22817400  -0.00015600
H  -1.91740900   1.90590000   0.87848600
H  -1.91681800   1.90660200  -0.87735100
```

Pd	1.27237000	2.03751200	-0.00005100
Cl	2.56731100	3.99803000	0.00021600

TS: $PdCl^-$ + $CH_3H_2C–CH_2CH_3$

E = -1742.85
H = -1658.21
G = -1687.92
N_{imag} = 1, -468.80 cm^{-1}

C	-0.95810400	-2.04671500	0.01325600
C	0.95795300	-2.04681200	-0.01320200
C	-1.45120700	-2.55118200	1.37275600
C	1.45101900	-2.55133800	-1.37269300
H	-1.65415700	-1.25648800	-0.36849000
H	-0.99547600	-2.84341900	-0.73772700
H	0.99525300	-2.84349700	0.73780400
H	1.65408300	-1.25664100	0.36852500
H	-2.44016200	-3.03111800	1.28022300
H	-0.76682500	-3.29527000	1.80932200
H	-1.54270800	-1.72470100	2.08497000
H	2.43972300	-3.03177900	-1.28007700
H	0.76634500	-3.29502600	-1.80948100
H	1.54303500	-1.72480200	-2.08477800
Pd	0.00001500	-0.06615700	0.00005900
Cl	0.00011200	2.33144500	0.00023100

P: $PdCl^-$ + $CH_3H_2C–CH_2CH_3$

E = -1766.56
H = -1681.14
G = -1711.44
N_{imag} = 0

C	0.25806100	-0.04502900	0.15596300
C	2.14286300	2.18817400	-0.03458800
C	-0.58835300	0.54002700	-0.97614100
C	1.39407800	2.88468200	1.11221500
H	0.13189400	-1.13318300	0.22273500
H	0.00350400	0.40910400	1.12248400
H	1.74315800	2.53022500	-1.00343900
H	3.21114100	2.47683500	-0.00242700
H	-1.66527800	0.34194800	-0.81235000
H	-0.46425800	1.62670400	-1.05855900
H	-0.31700600	0.10075100	-1.94365300
H	1.46817700	3.98821300	1.06109400
H	0.32378700	2.63856300	1.10112200
H	1.78470700	2.57463800	2.09082100
Pd	2.28676900	0.11789600	-0.07434300
Cl	3.07535600	-2.19144600	-0.23783400

RC: $PdCl^-$ + $(CH_3)_2HC-CH(CH_3)_2$

E = -2503.18

H = -2381.24

G = -2414.31

N_{imag} = 1, -25.48 cm^{-1}

C	0.54955700	2.50888700	0.95605500
H	1.27341600	3.67578800	2.65908400
C	-0.93900100	2.92586100	1.08386900
C	1.31429800	2.63856400	2.28630800
C	1.28727800	3.28846600	-0.14811200
H	1.24293600	4.37300100	0.04555400
C	-1.69999100	2.77112100	-0.24883900
C	-1.67281800	2.12438800	2.17858600
H	0.90516300	1.97697800	3.05400300
H	2.36848000	2.36833400	2.14665100
H	-0.94321300	3.99561800	1.36888900
Pd	-0.12485100	-0.19129000	0.24998300
H	2.34272200	2.99181300	-0.18243500
H	0.85996100	3.09416700	-1.13492300
Cl	-0.38985200	-2.46038800	-0.34095900
H	-2.74036800	2.38412400	2.19650900
H	-1.57712900	1.04513400	1.95316900
H	-1.26393100	2.30940900	3.17878600
H	-2.76568900	3.00078200	-0.11315600
H	-1.31033300	3.43031400	-1.03319400
H	-1.60712500	1.72332500	-0.59144600
H	0.63555100	1.40893500	0.66118200

TS: $PdCl^-$ + $(CH_3)_2HC-CH(CH_3)_2$

E = -2470.33

H = -2349.84

G = -2382.71

N_{imag} = 1, -403.76 cm^{-1}

C	1.70337100	-1.84049900	0.67759100
H	1.58761800	-2.13971400	-1.44671400
H	4.43264500	-0.68573600	-2.80881500
Cl	3.28087500	-5.27780100	-2.90878800
C	4.23824600	-1.44519700	-0.77120000
H	4.30863500	0.60174200	-1.58711800
Pd	3.24157000	-3.18970400	-1.72671100
H	0.74957900	0.09325800	-0.98827900
H	4.86218600	-2.36668000	-0.93647900
C	2.18475100	-1.50135900	-0.73767800
C	1.83444300	-0.06435200	-1.12030800
C	4.70853300	-0.40036700	-1.78929400
H	2.34319900	0.68522300	-0.49558200
H	2.07075300	0.14689100	-2.16788700
H	0.60051300	-1.80313100	0.70831500
H	2.00804200	-2.84973800	0.97022400
H	2.06841000	-1.13286100	1.43319300
H	4.01361100	-0.10490400	0.96591000

H	4.32769000	-1.80429600	1.38300600
H	5.63091500	-0.78841700	0.74313900
H	5.80900000	-0.32102400	-1.75127900
C	4.55320600	-1.01395800	0.66022200

P: $PdCl^-$ + $(CH_3)_2HC\text{–}CH(CH_3)_2$

E = -2500.47
H = -2379.23
G = -2413.04
N_{imag} = 0

C	-1.54359200	-0.94537000	0.02540300
C	1.56101700	-1.52225700	-0.20625700
H	-2.18472100	-0.09537100	-0.24143300
C	-1.70940600	-2.05296700	-1.00737800
C	-1.87280100	-1.40951200	1.44393400
C	1.46302700	-2.67823100	0.79255100
C	1.72985800	-2.04295000	-1.63955300
H	2.46683200	-0.92923700	0.05037700
H	1.89074800	-1.22295200	-2.35016400
Pd	0.25237300	0.08157400	0.01786900
Cl	-0.65291200	2.34427700	0.33872200
H	-2.89753200	-1.83345200	1.48399500
H	-1.18579900	-2.19350600	1.78630800
H	-1.82318100	-0.57655000	2.15253000
H	-2.75270300	-2.42746400	-1.00349200
H	-1.48430300	-1.69917800	-2.01956500
H	-1.06078800	-2.91236300	-0.79796900
H	2.32407100	-3.37372700	0.70743900
H	1.42429100	-2.31591300	1.82693600
H	0.55829200	-3.27831100	0.62148800
H	2.59101600	-2.73945500	-1.72517100
H	0.84228500	-2.59623000	-1.97267300

RC: $PdCl^-$ + $(CH_3)_3C\text{–}C(CH_3)_3$

E = -3237.21
H = -3078.83
G = -3115.42
N_{imag} = 0

C	0.64775300	2.67635000	1.05504200
C	1.14827800	1.22724600	0.81109500
C	-0.94641500	2.78325100	1.08949900
C	1.26421600	3.15423400	2.39694600
C	1.24497900	3.56179800	-0.07001400
C	-1.39801900	4.20235400	1.52414600
C	-1.56015100	2.49151800	-0.30239600
C	-1.55580900	1.76355000	2.08328200
H	2.24451500	1.21936000	0.80827600
H	0.82079500	0.52166500	1.59917400
H	0.84953500	0.87057000	-0.23429700
Pd	-0.03583900	-0.72313700	0.05458800
H	-2.49077100	4.27108400	1.45007500

H	-1.12499200	4.42213500	2.56273000
H	-0.97574700	4.98760800	0.88525300
H	-2.64067200	1.92141100	2.14872900
H	-1.38685700	0.73307300	1.73815500
H	-1.14264500	1.87146200	3.09362900
H	-2.65539400	2.50995400	-0.22736700
H	-1.26550300	3.24040700	-1.04722800
H	-1.27121300	1.49176000	-0.65735100
H	1.08964200	4.22194000	2.57537900
H	0.86566600	2.59353900	3.24900500
H	2.34992900	2.99539600	2.37677900
H	0.94147600	4.61214400	0.01872400
H	2.34111500	3.52845500	-0.01758600
H	0.95262700	3.19998600	-1.06024500
Cl	-1.19797300	-2.67396000	-0.54586400

TS: $PdCl^-$ + $(CH_3)_3C{-}C(CH_3)_3$

E = -3180.27
H = -3025.11
G = -3061.06
N_{imag} = 1, -234.25 cm^{-1}

C	1.19914000	2.08571100	0.99857300
C	1.98240600	0.87254900	1.51969300
C	-1.16273100	2.05061400	0.97229000
C	1.37052100	3.18317100	2.05014100
C	1.66225100	2.59351500	-0.36156600
C	-1.38077800	3.50264100	1.40367800
C	-1.96147000	1.87756700	-0.32935500
C	-1.58540800	1.10236100	2.08731900
H	3.02769900	1.18892700	1.74484500
H	1.55405800	0.49584500	2.45602300
H	2.02254600	0.03617700	0.80554800
Pd	0.05689700	0.09200600	-0.22597900
H	-2.47234600	3.68412000	1.39713000
H	-1.02682700	3.72366800	2.41275200
H	-0.93709100	4.22224200	0.70534800
H	-2.62181900	1.33597900	2.41264500
H	-1.54768200	0.05600400	1.76228100
H	-0.93668900	1.20932100	2.96614700
H	-3.01687900	2.18676700	-0.14277600
H	-1.57427400	2.52721000	-1.12309200
H	-1.96988800	0.84551200	-0.71015600
H	0.99682000	4.16037100	1.73725800
H	0.91930300	2.92109600	3.01449200
H	2.45749900	3.29444000	2.22660400
H	1.00824100	3.39572500	-0.72696900
H	2.68758700	3.01524000	-0.28150300
H	1.66885900	1.79353000	-1.11206100
Cl	0.07185200	-1.95571000	-1.45880900

P: PdCl^{-} + $(CH_3)_3C–C(CH_3)_3$

E = -3235.81

H = -3078.84

G = -3116.45

N_{imag} = 0

C	-1.64998300	-1.03780900	-0.02096200
C	1.52759900	-1.40602900	-0.19459300
C	-2.71635400	-0.01814900	-0.46108900
C	-1.68323700	-2.23282300	-0.97490500
C	-1.92497600	-1.49322100	1.41843400
C	1.44642400	-2.57623100	0.78831600
C	1.66299100	-1.92115100	-1.63286600
C	2.75401700	-0.53638100	0.15018200
H	-3.72035200	-0.49065900	-0.42289200
H	-2.72294600	0.86545700	0.18263800
H	-2.54520500	0.32265700	-1.48855100
H	-2.91691800	-1.99025600	1.47774500
H	-1.17610300	-2.20948600	1.77180600
H	-1.93680000	-0.64228900	2.10817800
H	-2.69246600	-2.69254200	-0.96351800
H	-1.47000900	-1.92893500	-2.00607700
H	-0.97052100	-3.01380500	-0.69476600
H	2.35177900	-3.21565500	0.71550000
H	1.37106800	-2.22361400	1.82405500
H	0.58363200	-3.22193500	0.59189100
H	2.56943000	-2.55604200	-1.73822000
H	0.80493400	-2.52891600	-1.93529900
H	1.75439700	-1.09263000	-2.34569600
H	3.69404200	-1.10690800	-0.00325000
H	2.81481300	0.35640100	-0.48799700
H	2.73675900	-0.20331500	1.19516800
Pd	0.09886300	0.11994600	0.01839600
Cl	-0.34847500	2.51982000	0.39227100

Table S3. Cartesian coordinates (in Å), energies (E, H and G, in kcal mol^{-1}), and number of imaginary vibrational frequencies (N_{imag}) of all stationary points of the C–H activation reactions in the gas phase, computed at ZORA-BLYP-D3(BJ)/TZ2P.

R: H_3C–H
E = -536.50
H = -506.79
G = -520.05
N_{imag} = 0

C	0.00000000	0.00000000	0.00000000
H	0.63179000	0.63179000	-0.63179000
H	-0.63179000	0.63179000	0.63179000
H	0.63179000	-0.63179000	0.63179000
H	-0.63179000	-0.63179000	-0.63179000

R: CH_3H_2C–H
E = -900.45
H = -852.17
G = -869.07
N_{imag} = 0

C	0.00000000	0.00000000	0.76939100
H	0.00000000	1.02244700	1.16701000
H	0.88546500	-0.51122300	1.16701000
H	-0.88546500	-0.51122300	1.16701000
C	0.00000000	0.00000000	-0.76939100
H	-0.88546500	0.51122300	-1.16701000
H	0.88546500	0.51122300	-1.16701000
H	0.00000000	-1.02244700	-1.16701000

R: $(CH_3)_2HC$–H
E = -1266.37
H = -1199.88
G = -1219.05
N_{imag} = 0

C	0.00000000	0.00000000	0.02094500
C	1.28119400	0.00000000	0.87213100
C	-1.28119400	0.00000000	0.87213100
H	0.00000000	0.87932900	-0.63858500
H	0.00000000	-0.87932900	-0.63858500
H	2.18105200	0.00000000	0.24508500
H	1.32251400	0.88587900	1.51945600
H	1.32251400	-0.88587900	1.51945600
H	-2.18105200	0.00000000	0.24508500
H	-1.32251400	-0.88587900	1.51945600
H	-1.32251400	0.88587900	1.51945600

R: $(CH_3)_3C$–H

E = -1633.44
H = -1549.08
G = -1570.66
N_{imag} = 0

C	0.00000000	0.00000000	0.36109500
C	1.46633500	0.00000000	-0.11122800
C	-0.73316800	-1.26988400	-0.11122800
C	-0.73316800	1.26988400	-0.11122800
H	0.00000000	0.00000000	1.46223100
H	2.00085300	0.88759000	0.24940600
H	2.00085300	-0.88759000	0.24940600
H	1.51826500	0.00000000	-1.20881700
H	-1.76910200	1.28899500	0.24940600
H	-0.23175100	2.17658500	0.24940600
H	-0.75913200	1.31485600	-1.20881700
H	-1.76910200	-1.28899500	0.24940600
H	-0.75913200	-1.31485600	-1.20881700
H	-0.23175100	-2.17658500	0.24940600

R: $PdCl^-$

E = -121.87
H = -119.13
G = -136.80
N_{imag} = 0

Pd	0.00000000	0.00000000	-0.01116000
Cl	0.00000000	0.00000000	2.32854700

R: $Pd(PH_3)_2$

E = -767.39
H = -730.11
G = -754.65
N_{imag} = 0

Pd	0.00000000	0.00000000	0.00000000
P	0.00000000	0.00000000	2.27833700
H	0.60992000	1.05641300	3.00983000
H	0.60992000	-1.05641300	3.00983000
H	-1.21984000	0.00000000	3.00983000
P	0.00000000	0.00000000	-2.27833700
H	-1.21984000	0.00000000	-3.00983000
H	0.60992000	-1.05641300	-3.00983000
H	0.60992000	1.05641300	-3.00983000

R: $Pd(PH_3)_2Cl^-$

E = - 872.25
H = - 833.90
G = - -865.49
N_{imag} = 0

Pd	0.00000000	0.00000000	0.00000000
P	0.00000000	0.00000000	2.27833700
H	0.60992000	1.05641300	3.00983000

H   0.60992000 -1.05641300   3.00983000
H -1.21984000   0.00000000   3.00983000
P   0.00000000   0.00000000 -2.27833700
H -1.21984000   0.00000000 -3.00983000
H   0.60992000 -1.05641300 -3.00983000
H   0.60992000   1.05641300 -3.00983000

RC: Pd + H_3C–H
E = -546.93
H = -516.00
G = -535.89
N_{imag} = 0
C   0.08877100   0.11135500   0.00000000
H   0.87756900 -0.64364600   0.00000000
H   0.18785400   0.70929700   0.93969100
H -0.90170600 -0.34816700   0.00000000
H   0.18785400   0.70929700 -0.93969100
Pd 0.49702100   2.54053000   0.00000000

TS: Pd + H_3C–H
E = -535.52
H = -508.36
G = -527.13
N_{imag} = 1, -771.93 cm^{-1}
C -0.13613000   0.16969800 -0.13615800
H   0.89358600 -0.09917700 -0.37942800
H -0.16303300   1.15843700   0.34631800
H -0.59124300 -0.57664200   0.51782100
H -0.73097800 -0.51844700 -1.48009900
Pd -1.32418900   0.90400000 -1.71927400

P: Pd + H_3C–H
E = -542.58
H = -514.10
G = -534.12
N_{imag} = 0
C   0.00012400 -0.00014200   0.00000000
H   0.37089100 -0.49025400 -0.90371300
H   0.23738100   1.07435000   0.00000000
H   0.37089100 -0.49025400   0.90371300
H -1.94358200 -1.53553000   0.00000000
Pd -2.02037400   0.00012000   0.00000000

RC: Pd + CH_3H_2C–H
E = -912.03
H = -862.43
G = -884.83
N_{imag} = 0
C   0.18942700 -2.76422900   0.00000000
C   0.90813100 -1.40770900   0.00000000
H   0.45740800 -3.35109400 -0.88686500

H -0.89717600 -2.63155900   0.00000000
H   0.45740800 -3.35109400   0.88686500
H   1.99892400 -1.49985500   0.00000000
H   0.65219800 -0.83773600 -0.93036100
H   0.65219800 -0.83773600   0.93036100
Pd -0.24376400   0.78828300   0.00000000

TS: Pd + $CH_3H_2C{-}H$
E = -900.13
H = -853.94
G = -875.64
N_{imag} = 1, -784.51 cm^{-1}

C	1.30663800	-0.65255300	-0.22935800
C	2.43169400	0.34981200	0.03907300
H	2.19332900	1.34301700	-0.35369300
H	2.63606700	0.45459300	1.10973400
H	3.35800600	0.00875600	-0.44860800
H	1.08276400	-0.75418400	-1.30326700
H	1.54433100	-1.65170200	0.14657700
H	0.31841800	-0.52985500	1.05919200
Pd	-0.68900900	0.06151700	0.02050100

P: Pd + $CH_3H_2C{-}H$
E = -908.42
H = -861.63
G = -883.80
N_{imag} = 0

C	1.21526100	-0.60678300	-0.36188000
C	2.34814600	0.29758100	0.10772500
H	2.26182400	1.31281000	-0.29318300
H	2.38321900	0.36394600	1.19940100
H	3.31326400	-0.11293300	-0.23389300
H	1.17069000	-0.68753600	-1.46284900
H	1.26463600	-1.60875400	0.07655000
H	-0.44073900	0.02052500	1.48408000
Pd	-0.66862300	0.06906600	-0.04195300

RC: Pd + $(CH_3)_2HC{-}H$
E = -1278.97
H = -1211.11
G = -1235.18
N_{imag} = 0

C	1.28923500	0.00000000	-0.91171500
C	0.00000000	0.00000000	-0.07570700
C	-1.28923500	0.00000000	-0.91171500
H	2.17698400	0.00000000	-0.27114000
H	1.33280800	-0.88745700	-1.55645400
H	1.33280800	0.88745700	-1.55645400
H	0.00000000	-0.92592700	0.56045500
H	0.00000000	0.92592700	0.56045500
H	-2.17698400	0.00000000	-0.27114000

H -1.33280800   0.88745700 -1.55645400
H -1.33280800 -0.88745700 -1.55645400
Pd 0.00000000   0.00000000   2.41838200

TS: Pd + $(CH_3)_2$HC–H
E = -1266.28
H = -1202.05
G = -1225.69
N_{imag} = 1, -761.81 cm^{-1}

C	-0.75130900	-0.42475000	1.28216900
C	-0.03140300	0.00225300	0.00000000
C	-0.75130900	-0.42475000	-1.28216900
H	-0.21063400	-0.10075400	2.17646000
H	-1.75571400	0.02814300	1.30662500
H	-0.87483300	-1.51260600	1.33598300
H	1.10370800	-1.18904800	0.00000000
H	-0.21063400	-0.10075400	-2.17646000
H	-0.87483300	-1.51260600	-1.33598300
H	-1.75571400	0.02814300	-1.30662500
H	0.09097000	1.09874400	0.00000000
Pd	2.12427000	-0.00792200	0.00000000

P: Pd + $(CH_3)_2$HC–H
E = -1276.00
H = -1211.30
G = -1235.56
N_{imag} = 0

C	-0.60126500	-0.41108100	1.27689900
C	0.02772800	0.13416400	0.00000000
C	-0.60126500	-0.41108100	-1.27689900
H	-0.12543500	-0.01146100	2.17773100
H	-1.66961300	-0.13374800	1.30768400
H	-0.54190500	-1.50401300	1.31150900
H	2.07085700	-1.54488500	0.00000000
H	-0.12543500	-0.01146100	-2.17773100
H	-0.54190500	-1.50401300	-1.31150900
H	-1.66961300	-0.13374800	-1.30768400
H	0.01172100	1.24327300	0.00000000
Pd	2.05901700	0.00534700	0.00000000

RC: Pd + $(CH_3)_3$C–H
E = -1645.43
H = -1560.16
G = -1588.02
N_{imag} = 0

C	-0.01166100	-0.04631800	0.00000000
C	1.52086600	0.03791800	0.00000000
C	-0.54941000	-0.71576300	1.27198400
C	-0.54941000	-0.71576300	-1.27198400
H	-0.40141800	1.02879400	0.00000000
H	1.88850900	0.56518200	-0.88751400

```
H   1.88850900   0.56518200   0.88751400
H   1.95502000  -0.97247800   0.00000000
H  -1.64516800  -0.72212300  -1.28340500
H  -0.20123600  -0.19508800  -2.17114000
H  -0.20235000  -1.75790300  -1.32619400
H  -1.64516800  -0.72212300   1.28340500
H  -0.20235000  -1.75790300   1.32619400
H  -0.20123600  -0.19508800   2.17114000
Pd -1.03892100   2.73987700   0.00000000
```

TS: Pd + $(CH_3)_3C$–H

E = -1630.85
H = -1548.65
G = -1574.82
N_{imag} = 1, -785.63 cm^{-1}

```
C   0.05282600   0.07242200   0.00000000
C   1.22580400  -0.93200000   0.00000000
C   0.11368000   0.93556100  -1.26934000
C   0.11368000   0.93556100   1.26934000
H  -1.51643500  -0.00993500   0.00000000
H   1.23800200  -1.56798900   0.89124600
H   1.23800200  -1.56798900  -0.89124600
H   2.16703100  -0.34988400   0.00000000
H  -0.68799100   1.68132300   1.29629100
H  -0.68799100   1.68132300  -1.29629100
H   1.07828200   1.47000500   1.29709000
H   1.07828200   1.47000500  -1.29709000
H   0.03892000   0.32247800  -2.17304100
H   0.03892000   0.32247800   2.17304100
Pd -1.46991800  -1.56703700   0.00000000
```

P: Pd + $(CH_3)_3C$–H

E = -1645.29
H = -1562.81
G = -1588.93
N_{imag} = 0

```
C  -0.01347100  -0.01514500   0.00000000
C   0.64017100  -0.54615500  -1.26822400
C   0.64017100  -0.54615500   1.26822400
C  -1.54747200  -0.05430600   0.00000000
H   1.33078500   2.50819100   0.00000000
H   0.19055800  -0.12294400  -2.17163200
H   1.71145300  -0.32399500  -1.27842700
H   0.52242700  -1.64396100  -1.31111300
H  -1.99745700   0.97887500   0.00000000
H  -1.95405700  -0.53118200  -0.89820400
H  -1.95405700  -0.53118200   0.89820400
H   0.19055800  -0.12294400   2.17163200
H   0.52242700  -1.64396100   1.31111300
H   1.71145300  -0.32399500   1.27842700
Pd -0.16689000   2.01141000   0.00000000
```

RC: Pd(PH_3)$_2$ + H_3C–H

E = -1305.98
H = -1239.08
G = -1269.66
N_{imag} = 0

Pd	0.02959800	-0.43885300	0.00000000
P	2.30856200	-0.38598300	0.00000000
H	3.02036600	0.84466500	0.00000000
H	3.05040200	-0.98468800	-1.05541900
H	3.05040200	-0.98468800	1.05541900
P	-2.25028500	-0.42554000	0.00000000
H	-2.98080400	-1.03813900	1.05532000
H	-2.98080400	-1.03813900	-1.05532000
H	-2.98484400	0.79175500	0.00000000
H	-0.01416300	-3.09837000	0.00000000
C	-0.09944900	-4.19381700	0.00000000
H	0.90053600	-4.63912400	0.00000000
H	-0.64201900	-4.51616600	0.89428600
H	-0.64201900	-4.51616600	-0.89428600

TS: Pd(PH_3)$_2$ + H_3C–H

E = -1277.13
H = -1212.56
G = -1242.18
N_{imag} = 1, -740.97 cm^{-1}

C	-0.02224400	0.00743200	0.00000000
Pd	2.18536800	-0.03005600	0.00000000
H	-0.68424400	-0.85924300	0.00000000
H	-0.21165100	0.59660000	-0.90233000
H	-0.21165100	0.59660000	0.90233000
H	1.14930700	-1.24620200	0.00000000
H	3.52227300	2.87286300	-1.05049100
H	5.51140600	-0.57152600	0.00000000
H	4.49540700	-2.07947400	-1.05316000
H	3.52227300	2.87286300	1.05049100
H	1.74769100	3.29494800	0.00000000
H	4.49540700	-2.07947400	1.05316000
P	2.75755800	2.28853600	0.00000000
P	4.21392000	-1.16535000	0.00000000

P: Pd(PH_3)$_2$ + H_3C–H

E = -1282.76
H = -1217.33
G = -1246.52
N_{imag} = 0

C	-0.02104600	0.02960100	0.00000000
Pd	2.10251500	-0.04050800	0.00000000
H	-0.48870800	-0.95430200	0.00000000
H	-0.32616100	0.58296600	-0.89644100
H	-0.32616100	0.58296600	0.89644100
H	1.81164100	-1.61242700	0.00000000

H	2.83575200	3.03813800	-1.05618100
H	5.47344500	0.26903300	0.00000000
H	4.82917400	-1.44470000	-1.05929100
H	2.83575200	3.03813800	1.05618100
H	0.99261200	3.06162400	0.00000000
H	4.82917400	-1.44470000	1.05929100
P	2.20628000	2.32141500	0.00000000
P	4.36033400	-0.62311900	0.00000000

RC: $Pd(PH_3)_2$ + CH_3H_2C–H

E = -1671.13
H = -1584.99
G = -1619.18
N_{imag} = 0

C	1.53277900	2.76822300	0.76567100
C	0.78767800	2.76212900	-0.57771000
H	1.54838200	3.76900300	1.21597700
H	1.05702400	2.07911600	1.47473400
H	2.57341100	2.44296600	0.64132100
H	1.28357600	3.40340200	-1.31733100
H	-0.24221100	3.12147500	-0.46077200
H	0.73377400	1.74365800	-0.98476000
Pd	0.23240200	-0.70717400	0.52025200
P	2.46048200	-1.18077100	0.50513200
H	3.13666500	-1.58142700	1.69034000
H	3.39233500	-0.17136500	0.14039800
H	3.01193300	-2.19639100	-0.32462800
P	-1.97594200	-0.14711100	0.57913400
H	-2.78242300	-0.42523700	1.71652300
H	-2.89928800	-0.65011900	-0.37937500
H	-2.36372400	1.21452200	0.45190600

TS: $Pd(PH_3)_2$ + CH_3H_2C–H

E = -1641.28
H = -1557.68
G = -1591.45
N_{imag} = 1, -735.63 cm^{-1}

Pd	-0.11125900	-0.03291100	-0.05302600
H	1.86857400	2.63166400	0.35684200
H	1.88552800	-2.73326600	-0.30455200
H	0.04040800	-3.23714200	0.56331100
H	2.88882500	0.95333600	1.11026400
H	2.82258300	1.29924000	-0.96065400
H	0.18085100	-2.93913400	-1.51515900
P	1.89892700	1.22503800	0.12157700
P	0.54364700	-2.24680700	-0.32592900
C	-1.68690100	1.54807900	0.13484700
C	-1.71638200	2.04224400	1.58583400
H	-1.70892700	-0.16644700	-0.07651800
H	-2.35920500	2.93179500	1.68214200
H	-0.71814500	2.31343000	1.94837600

H	-2.11219300	1.27758200	2.26372400
H	-1.20238600	2.25997000	-0.54272500
H	-2.70315800	1.40608700	-0.24007200

P: $Pd(PH_3)_2$ + CH_3H_2C–H

E = -1646.22
H = -1562.47
G = -1593.72
N_{imag} = 0

C	2.15388200	-0.01908300	0.00000000
Pd	0.01946600	0.04159400	0.00000000
C	2.96153100	1.28355500	0.00000000
H	2.39830100	-0.62262800	-0.88617800
H	2.39830100	-0.62262800	0.88617800
H	0.24038600	1.62623700	0.00000000
H	-0.66025700	-3.04377000	-1.05612600
H	-3.36279000	-0.34587300	0.00000000
H	-2.74519700	1.37687800	-1.05852700
H	-0.66025700	-3.04377000	1.05612600
H	1.18366100	-3.03976100	0.00000000
H	-2.74519700	1.37687800	1.05852700
P	-0.04161000	-2.31891100	0.00000000
P	-2.26208300	0.56307300	0.00000000
H	4.04485200	1.07541500	0.00000000
H	2.74041700	1.89332700	0.88283700
H	2.74041700	1.89332700	-0.88283700

RC: $Pd(PH_3)_2$ + $(CH_3)_2HC$–H

E = -2037.92
H = -1933.68
G = -1968.89
N_{imag} = 0

C	-3.46213100	-1.35229900	1.26591800
C	-3.37555200	-0.25154600	0.19823800
C	-3.54498700	-0.78811300	-1.23072500
H	-3.30379500	-0.94811600	2.27304700
H	-4.44205600	-1.84834300	1.24970800
H	-2.70084700	-2.12483300	1.09347800
H	-2.39217500	0.24531100	0.27454400
H	-3.47455400	0.01762500	-1.97139400
H	-2.76146900	-1.52043600	-1.46519700
H	-4.51658200	-1.28368500	-1.35917800
H	-4.13342000	0.51883800	0.39941400
Pd	0.07806800	0.41104800	-0.05049300
H	1.25772600	3.36112900	-0.72943900
H	1.60448000	-2.45811700	0.10076700
H	-0.40094500	3.56501600	0.56092500
H	-0.70318900	3.21103300	-1.49679800
H	-0.41015400	-2.78001500	-0.44603500
H	0.08722700	-2.40902700	1.56953200
P	0.05980600	2.65569100	-0.43202500

P	0.34462500	-1.82956400	0.29622400

TS: Pd(PH$_3$)$_2$ + (CH$_3$)$_2$HC–H

E = -2006.78

H = -1905.71

G = -1939.75

N_{imag} = 1, -685.00 cm^{-1}

C	-2.97465500	-0.50109400	1.27943900
C	-2.29239200	-0.00794000	0.00000000
C	-2.97465500	-0.50109400	-1.27943900
H	-2.45699500	-0.14420200	2.17684900
H	-4.01495600	-0.13768000	1.32373400
H	-3.00143100	-1.59692300	1.32646200
H	-1.07431400	-1.22738700	0.00000000
H	-2.45699500	-0.14420200	-2.17684900
H	-3.00143100	-1.59692300	-1.32646200
H	-4.01495600	-0.13768000	-1.32373400
H	-2.30901200	1.08752800	0.00000000
Pd	-0.02450900	-0.02255800	0.00000000
H	1.37380700	2.83222200	-1.05073600
H	3.30600700	-0.63058300	0.00000000
H	1.37380700	2.83222200	1.05073600
H	-0.38584800	3.31110400	0.00000000
H	2.25326000	-2.11244900	-1.05247200
H	2.25326000	-2.11244900	1.05247200
P	0.59178400	2.27103500	0.00000000
P	1.99260200	-1.19114500	0.00000000

P: Pd(PH$_3$)$_2$ + (CH$_3$)$_2$HC–H

E = -2012.85

H = -1911.63

G = -1943.23

N_{imag} = 0

C	-2.81660400	-0.18870400	1.27349700
C	-2.14597900	0.33876100	0.00000000
C	-2.81660400	-0.18870400	-1.27349700
H	-2.32766300	0.18344300	2.18182700
H	-3.87374100	0.13026800	1.31111800
H	-2.79518600	-1.28354600	1.31044400
H	-0.56349600	-1.56065300	0.00000000
H	-2.32766300	0.18344300	-2.18182700
H	-2.79518600	-1.28354600	-1.31044400
H	-3.87374100	0.13026800	-1.31111800
H	-2.22660100	1.43500700	0.00000000
Pd	-0.02986100	-0.04259100	0.00000000
H	1.27628100	2.81437600	-1.05534500
H	3.38064200	-0.32716100	0.00000000
H	1.27628100	2.81437600	1.05534500
H	-0.51827400	3.22779500	0.00000000
H	2.42600900	-1.88806800	-1.05829200
H	2.42600900	-1.88806800	1.05829200

P   0.51004000   2.24495700   0.00000000
P   2.11716300 -0.99320600   0.00000000

RC: Pd$(PH_3)_2$ + $(CH_3)_3$C–H

E = -2405.82
H = -2283.77
G = -2321.51
N_{imag} = 0

C -2.03072500 -4.68970800   1.27081800
C -2.73310200 -4.18591700   0.00000000
C -2.03072500 -4.68970800 -1.27081800
H -2.50897000 -4.28736500   2.17230200
H -2.06335400 -5.78692100   1.33118800
H -0.97655700 -4.38492200   1.28580800
H -2.65907200 -3.07812900   0.00000000
H -2.50897000 -4.28736500 -2.17230200
H -0.97655700 -4.38492200 -1.28580800
H -2.06335400 -5.78692100 -1.33118800
Pd -2.42317200 -0.78238000   0.00000000
H -5.29043900   0.33327700 -1.05548100
H   0.49500700 -2.20506800   0.00000000
H -5.29043900   0.33327700   1.05548100
H -5.59772600 -1.46921200   0.00000000
H   0.63033200 -0.38312400 -1.05513200
H   0.63033200 -0.38312400   1.05513200
P -4.66988200 -0.39063400   0.00000000
P -0.14734400 -0.93509800   0.00000000
C -4.22788300 -4.54354600   0.00000000
H -4.73049100 -4.13815400 -0.88731600
H -4.73049100 -4.13815400   0.88731600
H -4.37368900 -5.63314500   0.00000000

TS: Pd$(PH_3)_2$ + $(CH_3)_3$C–H

E = -2371.80
H = -2252.84
G = -2287.96
N_{imag} = 1, -652.16 cm^{-1}

C -0.68211500 -0.64060300   1.26764900
C -0.07386400 -0.02129100   0.00000000
C -0.68211500 -0.64060300 -1.26764900
H -0.20321700 -0.25016600   2.17288900
H -1.75736100 -0.39620800   1.32526300
H -0.58179300 -1.73178200   1.27931500
H   1.19552400 -1.14533800   0.00000000
H -0.20321700 -0.25016600 -2.17288900
H -0.58179300 -1.73178200 -1.27931500
H -1.75736100 -0.39620800 -1.32526300
Pd 2.25684100   0.03339500   0.00000000
H   3.93617300   2.69849100 -1.05022300
H   5.55608800 -0.76825900   0.00000000
H   3.93617300   2.69849100   1.05022300

H	2.26937600	3.43863100	0.00000000
H	4.40855100	-2.17727700	-1.05215800
H	4.40855100	-2.17727700	1.05215800
P	3.07816400	2.26319600	0.00000000
P	4.20776700	-1.24066300	0.00000000
C	-0.36874800	1.49338300	0.00000000
H	0.02839300	1.99482200	-0.88836100
H	0.02839300	1.99482200	0.88836100
H	-1.46640900	1.63953100	0.00000000

RC: $PdCl^-$ + $H_3C–H$

E = -673.42
H = -641.47
G = -664.99
N_{imag} = 0

C	-1.81278000	-1.87757500	0.00000000
Pd	-0.21678100	-0.13249700	0.00000000
Cl	1.36418600	1.61295100	0.00000000
H	-2.08493900	-0.78243700	0.00000000
H	-2.22400300	-2.34003800	-0.90209400
H	-0.69120700	-2.02797200	0.00000000
H	-2.22400300	-2.34003800	0.90209400

TS: $PdCl^-$ + $H_3C–H$

E = -666.53
H = -637.08
G = -660.53
N_{imag} = 1, -662.31 cm^{-1}

C	-1.09155800	-2.12526500	0.00000000
Pd	-0.26691100	-0.15460400	0.00000000
Cl	1.26815700	1.67714600	0.00000000
H	-1.62539200	-2.47331300	0.89303600
H	-0.07743100	-2.55595500	0.00000000
H	-1.62539200	-2.47331300	-0.89303600
H	-1.73017300	-0.68605700	0.00000000

P: $PdCl^-$ + $H_3C–H$

E = -669.77
H = -639.72
G = -663.68
N_{imag} = 0

C	-0.89254600	-2.12801200	0.00000000
Pd	-0.31081600	-0.15108200	0.00000000
Cl	1.31485100	1.64618000	0.00000000
H	-1.46376800	-2.41516900	0.89335500
H	0.07460900	-2.65726000	0.00000000
H	-1.46376800	-2.41516900	-0.89335500
H	-1.84838400	-0.15045700	0.00000000

RC: PdCl$^-$ + CH_3H_2C–H

E = -1038.43
H = -987.99
G = -1013.98
N_{imag} = 0

C	-0.95023900	-1.26026700	0.00000000
C	-0.07092300	0.00002200	0.00000000
Pd	2.29578600	0.00726900	0.00000000
H	-1.59868000	-1.30069500	0.88845600
H	-0.32543500	-2.15985000	0.00000000
H	-1.59868000	-1.30069500	-0.88845600
H	-0.66468100	0.92151600	0.00000000
H	0.57364200	0.02842700	0.92894800
H	0.57364200	0.02842700	-0.92894800
Cl	4.64533900	-0.02127300	0.00000000

TS: PdCl$^-$ + CH_3H_2C–H

E = -1030.87
H = -983.09
G = -1008.37
N_{imag} = 1, -656.40 cm^{-1}

C	-0.11601500	-0.20796400	-2.07127300
Pd	-0.11952600	-0.16456200	0.07066200
C	1.24802500	-0.15450000	-2.78066300
H	1.92290700	0.54871700	-2.27952100
H	1.74437100	-1.13410500	-2.77774700
H	1.14490600	0.16511600	-3.83399000
H	-0.59141500	0.78658200	-2.06121300
H	-0.79937500	-0.87714400	-2.61179800
H	0.06636100	-1.32875400	-0.94971000
Cl	-0.23866800	0.65912800	2.30628000

P: PdCl$^-$ + CH_3H_2C–H

E = -1034.38
H = -985.94
G = -1012.42
N_{imag} = 0

C	-0.10565900	0.02311000	-0.19671400
Pd	1.54043400	0.21358000	-1.42330600
C	-0.34760300	-1.39935300	0.33195800
H	-0.31526100	-2.14067500	-0.47631000
H	0.41587500	-1.68682300	1.06517800
H	-1.33621200	-1.48835500	0.82418000
H	-0.89501800	0.31260800	-0.91132200
H	-0.12665200	0.74398000	0.63107700
H	2.07879200	0.32052000	0.02445600
Cl	2.28397900	0.24877700	-3.73344100

RC: PdCl⁻ + $(CH_3)_2HC-H$

E = -1405.59
H = -1336.98
G = -1365.17
N_{imag} = 0

C	-0.88728400	0.00007100	-1.28318600
C	-0.04545500	-0.00000500	0.00000000
C	-0.88728400	0.00007100	1.28318600
H	-0.24054100	0.00001200	-2.16682000
H	-1.53677200	-0.88850200	-1.33342700
H	-1.53660500	0.88876500	-1.33343400
H	0.61001100	-0.92256900	0.00000000
H	0.61013400	0.92252100	0.00000000
H	-0.24054100	0.00001200	2.16682000
H	-1.53660500	0.88876500	1.33343400
H	-1.53677200	-0.88850200	1.33342700
Pd	2.33686800	0.00019100	0.00000000
Cl	4.68496700	0.00017900	0.00000000

TS: PdCl⁻ + $(CH_3)_2HC-H$

E = -1397.38
H = -1331.69
G = -1358.84
N_{imag} = 1, -633.42 cm^{-1}

C	-0.75706800	-0.41796900	1.27244800
C	-0.00507200	0.00130100	0.00000000
C	-0.75706800	-0.41796900	-1.27244800
H	-0.22856000	-0.07692600	2.16945700
H	-1.77827500	0.00883600	1.28725300
H	-0.85548000	-1.51064700	1.34276100
H	1.10171500	-1.16775600	0.00000000
H	-0.22856000	-0.07692600	-2.16945700
H	-0.85548000	-1.51064700	-1.34276100
H	-1.77827500	0.00883600	-1.28725300
H	0.08679100	1.09993200	0.00000000
Pd	2.15304400	-0.01790600	0.00000000
Cl	4.40521800	0.76996700	0.00000000

P: PdCl⁻ + $(CH_3)_2HC-H$

E = -1401.75
H = -1335.12
G = -1363.27
N_{imag} = 0

C	-0.62519100	-0.28028300	1.26968800
C	0.13795400	0.10280500	0.00000000
C	-0.62519100	-0.28028300	-1.26968800
H	-0.07228300	0.00444000	2.17166900
H	-1.60931400	0.23188600	1.29660900
H	-0.80674000	-1.36099900	1.31307500
H	1.42708100	-1.95624700	0.00000000
H	-0.07228300	0.00444000	-2.17166900

H	-0.80674000	-1.36099900	-1.31307500
H	-1.60931400	0.23188600	-1.29660900
H	0.32609900	1.18726300	0.00000000
Pd	2.09099400	-0.52132500	0.00000000
Cl	3.55417400	1.43719500	0.00000000

RC: $PdCl^-$ + $(CH_3)_3C$–H

E = -1767.87
H = -1682.36
G = -1710.92
N_{imag} = 0

C	0.36506500	-1.03803900	0.00000000
C	1.90071900	-1.02612300	0.00000000
C	-0.19294300	-1.69738800	1.26948400
C	-0.19294300	-1.69738800	-1.26948400
H	0.02923600	0.03484500	0.00000000
H	2.28616700	-0.50827200	-0.88530600
H	2.28616700	-0.50827200	0.88530600
H	2.29258100	-2.05738700	0.00000000
H	-1.28755300	-1.65565800	-1.28080800
H	0.17062200	-1.18472300	-2.16689400
H	0.11781100	-2.75487000	-1.32080100
H	-1.28755300	-1.65565800	1.28080800
H	0.11781100	-2.75487000	1.32080100
H	0.17062200	-1.18472300	2.16689400
Pd	-0.59326300	1.71450800	0.00000000
Cl	-1.35947400	3.94372000	0.00000000

TS: $PdCl^-$ + $(CH_3)_3C$–H

E = -1762.55
H = -1678.88
G = -1708.15
N_{imag} = 1, -639.38 cm^{-1}

C	0.00087700	0.02262800	0.00000000
C	0.12904100	-1.51802200	0.00000000
C	0.70088800	0.56551000	-1.26124000
C	0.70088800	0.56551000	1.26124000
H	-1.10230200	1.05275600	0.00000000
H	-0.32896300	-1.96900700	0.88588900
H	-0.32896300	-1.96900700	-0.88588900
H	1.21045700	-1.78840800	0.00000000
H	0.67876300	1.66231100	1.29596800
H	0.67876300	1.66231100	-1.29596800
H	1.75992300	0.24247400	1.28143900
H	1.75992300	0.24247400	-1.28143900
H	0.21008400	0.19375100	-2.16757400
H	0.21008400	0.19375100	2.16757400
Pd	-2.23272200	-0.01818200	0.00000000
Cl	-4.49996200	-0.72046100	0.00000000

P: $PdCl^-$ + $(CH_3)_3C$–H

E = -1770.33
H = -1685.98
G = -1716.13
N_{imag} = 0

C	-0.13397700	-0.09816000	0.00000000
C	-0.17883200	-1.63567900	0.00000000
C	0.58714300	0.40197200	-1.26032600
C	0.58714300	0.40197200	1.26032600
H	-1.50493000	1.80871900	0.00000000
H	-0.69596400	-2.02375900	0.88442200
H	-0.69596400	-2.02375900	-0.88442200
H	0.85391800	-2.04323700	0.00000000
H	0.65967400	1.49452500	1.27102700
H	0.65967400	1.49452500	-1.27102700
H	1.61584000	-0.01467400	1.30203200
H	1.61584000	-0.01467400	-1.30203200
H	0.06188800	0.09058300	-2.17043000
H	0.06188800	0.09058300	2.17043000
Pd	-2.15333300	0.38583100	0.00000000
Cl	-4.05715100	-1.13905800	0.00000000

RC: $Pd(PH_3)_2Cl^-$ + $H_3C{-}H$
E = -1412.33
H = -1344.89
G = -1380.26
N_{imag} = 0

C	2.76407900	-0.36722300	-4.14688000
H	1.95611000	0.07191800	-3.55215900
H	2.79825300	0.09127000	-5.14198100
Cl	0.34075300	1.10674600	-1.48670300
Pd	2.36374600	0.11347000	-0.34893300
H	4.14357500	-2.57095500	-0.82106300
H	3.99376500	2.79318900	0.90166900
H	5.19103300	1.11277700	0.88244700
H	2.31865300	-2.97083300	-1.70803900
H	2.50371100	-3.04911700	0.35440100
H	3.71852500	1.31947400	2.32838900
P	2.79868000	-2.08510600	-0.66991800
P	3.77471900	1.36226600	0.89196600
H	3.71138100	-0.19234600	-3.62756500
H	2.60173500	-1.44593800	-4.24622000

TS: $Pd(PH_3)_2Cl^-$ + $H_3C{-}H$
E = -1380.57
H = -1315.09
G = -1348.14
N_{imag} = 1, -840.70 cm^{-1}

C	-0.05290300	0.10259100	0.58209800
H	-0.05636400	-0.58113500	1.43775000
H	-0.69417700	0.95659500	0.81941700
H	-0.45209700	-0.39285300	-0.30937500

H 1.09144600 1.23252400 0.44526600
Cl 6.31463200 -1.90184100 -1.33971000
Pd 2.12854900 0.06250700 0.07934500
H 2.63285500 -3.22603300 0.67119600
H 5.31891900 0.16443000 -0.68531700
H 4.87844900 1.77689700 0.70667000
H 4.15697300 -2.49246300 -0.70672900
H 2.14288600 -2.99105200 -1.35800100
H 4.38488200 2.01690900 -1.32258400
P 2.78817900 -2.22726300 -0.33862800
P 4.22586500 1.02146500 -0.31339200

P: $Pd(PH_3)_2Cl^-$ + H_3C-H

E = -1391.24
H = -1325.11
G = -1357.81
N_{imag} = 0

C 0.07803600 -0.45358900 0.50936000
H 0.10438400 -1.16765900 1.34391700
H -0.57450000 0.38272000 0.77270700
H -0.32371500 -0.95899000 -0.37958200
H 1.56103500 1.63290000 0.39152100
Cl 6.28042900 -1.92462000 -1.18823700
Pd 2.08602200 0.14111800 0.08244900
H 2.53300700 -3.07388400 0.68104600
H 5.30378700 0.11674700 -0.71184900
H 4.86153700 1.75376700 0.68177600
H 4.10093900 -2.41924600 -0.71995900
H 2.03338100 -2.82787500 -1.35959700
H 4.35961300 1.98941100 -1.35948900
P 2.74047700 -2.11404500 -0.35035300
P 4.22615300 1.00007200 -0.34493800

RC: $Pd(PH_3)_2Cl^-$ + CH_3H_2C-H

E = -1777.21
H = -1690.58
G = -1728.70
N_{imag} = 0

C 1.89525000 0.90696600 -4.27928100
H 2.00268100 0.14312500 -3.50215200
H 1.10326800 1.60912200 -3.98329100
Cl 2.69289200 -2.56317700 -3.19098200
Pd 3.29403500 -1.44382600 -1.01491200
H 4.27970600 -2.33745100 1.96514000
H 4.34020400 1.61678300 -1.91476400
H 4.41487100 1.39822200 0.14008800
H 3.53645800 -4.06907000 1.11850400
H 2.23073100 -2.60934100 1.78854800
H 2.59194800 1.69735200 -0.80524200
P 3.36099400 -2.64776300 0.90137100
P 3.68844500 0.77830700 -0.93674300

H	2.83780800	1.46883400	-4.32563600
C	1.58254300	0.23313800	-5.62431700
H	2.35231000	-0.50910200	-5.86249200
H	1.52412400	0.95954500	-6.44912400
H	0.62750200	-0.30581800	-5.57720600

TS: $Pd(PH_3)_2Cl^-$ + $CH_3H_2C–H$

E = -1743.51
H = -1660.42
G = -1694.30
N_{imag} = 1, -792.10 cm^{-1}

Pd	-0.01304000	0.11697500	0.17440200
Cl	4.14783200	-1.76057800	-1.41941600
H	3.15145400	0.26147500	-0.95135800
H	-0.13494300	-2.90619000	-1.14850900
H	1.93551200	-2.36989800	-0.75218400
H	2.14048800	2.12783000	-1.47909600
H	2.84319100	1.86961600	0.48464200
H	0.60148600	-3.05984700	0.81454900
P	2.09381300	1.11747500	-0.46997900
P	0.61860800	-2.10158400	-0.24220000
C	-1.40508500	1.76898400	0.81236100
C	-2.89289000	1.79884100	1.21304200
H	-1.51533600	0.13251300	0.71381900
H	-3.23449800	2.82642900	1.42436200
H	-3.08137600	1.19659700	2.11205000
H	-3.53119900	1.39716800	0.41473100
H	-0.78967400	2.16742000	1.62761400
H	-1.25231400	2.39111100	-0.07758500

P: $Pd(PH_3)_2Cl^-$ + $CH_3H_2C–H$

E = -1755.38
H = -1671.01
G = -1705.68
N_{imag} = 0

Pd	-0.35100900	0.00449500	-0.26753200
Cl	4.20258300	-1.63453900	-0.24530900
H	1.94432500	1.69053900	1.37433100
H	0.16766600	-3.17936300	0.24736000
H	0.26897900	-2.80658300	-1.83199300
H	2.97540200	0.25658100	0.05721900
H	2.11261700	2.14707300	-0.68310900
H	2.00500000	-2.31728600	-0.57692100
P	1.75916200	1.02946600	0.12615400
P	0.57821700	-2.12744500	-0.61953000
C	-1.39616800	1.85537600	0.04578900
C	-1.60218700	2.15809100	1.53893000
H	-1.83040900	-0.60139800	-0.51330300
H	-2.11569900	3.12460700	1.70101300
H	-0.64851100	2.20523300	2.08170400
H	-2.20807700	1.38163600	2.02342400

```
H  -0.79708400   2.64646700  -0.42755600
H  -2.36047900   1.82079200  -0.47103000
```

RC: Pd(PH_3)$_2$Cl$^-$ + (CH_3)$_2$HC–H

E = -2143.47
H = -2039.89
G = -2076.93
N_{imag} = 0

```
C   1.50643600   1.09036900  -5.57101800
H   1.31700000   1.30619900  -4.51067200
H   0.79871300   1.70306700  -6.15337300
Cl  3.99232000  -1.04794700  -3.31093100
Pd  3.75291600  -1.04847400  -0.80038300
H   4.43826500  -3.19304700   1.53380900
H   3.82627700   2.23138600   0.07381300
H   4.34494900   0.99053200   1.63977000
H   3.98565600  -4.36757000  -0.10351900
H   2.46384200  -3.43585800   0.94453100
H   2.35798800   1.16690400   1.06850000
P   3.68797700  -3.01524800   0.31962700
P   3.59858900   0.84360400   0.41963900
H   3.18172500   1.29145900  -6.96198800
C   1.24413400  -0.40409900  -5.82468100
H   1.91372700  -1.00892300  -5.20128100
H   1.42446100  -0.65983800  -6.87954400
H   0.20670600  -0.67583200  -5.58578200
C   2.95273100   1.49351600  -5.90501900
H   3.12393600   2.56372800  -5.72213700
H   3.64856700   0.91956500  -5.28159300
```

TS: Pd(PH_3)$_2$Cl$^-$ + (CH_3)$_2$HC–H

E = -2110.65
H = -2009.53
G = -2044.89
N_{imag} = 1, -797.86 cm^{-1}

```
C  -4.76674100  -2.13175300   1.13100400
C  -4.34675200  -1.30888500  -0.09227300
C  -4.64020100  -2.02637000  -1.41514900
H  -4.57488000  -1.58619600   2.06232300
H  -5.84448100  -2.37552600   1.09539100
H  -4.21392400  -3.08002700   1.18717500
H  -2.76892300  -1.67654700  -0.05281900
H  -4.34622200  -1.41090900  -2.27321900
H  -4.09102900  -2.97563400  -1.48932200
H  -5.71736800  -2.25473900  -1.51286300
H  -4.90920500  -0.36875900  -0.08407700
Pd -2.40267200  -0.11982100   0.08019200
H  -3.15212000   2.83098800   1.46759900
H   0.49065700   1.39667600  -0.21051500
H  -4.00811000   2.83501000  -0.45172900
H  -1.84334200   3.15644600  -0.21408600
```

H	0.63467200	-0.12085600	1.32688100
H	0.78071100	-0.72125500	-0.68327400
P	-2.87908100	2.24107200	0.19759000
P	-0.06936400	0.10876000	0.10783700
Cl	0.38804700	3.72706200	-0.51436700

P: $Pd(PH_3)_2Cl-$ + $(CH_3)_2HC-H$

E = -2121.85
H = -2019.50
G = -2056.12
N_{imag} = 0

C	-4.77626500	-1.63532500	1.26335100
C	-4.40424700	-0.85406900	-0.00426300
C	-4.78487900	-1.62792700	-1.27386400
H	-4.52426100	-1.07740000	2.17407700
H	-5.86325400	-1.85353400	1.29533000
H	-4.24287500	-2.59298700	1.30775400
H	-2.03938700	-1.87001500	-0.02363300
H	-4.53785000	-1.06521500	-2.18299700
H	-4.25265700	-2.58578900	-1.32685900
H	-5.87228500	-1.84483700	-1.30035400
H	-4.98104400	0.08378200	0.00035300
Pd	-2.32964000	-0.27611500	-0.00820200
H	-3.71947900	2.50786900	-1.03471200
H	0.76784400	-0.43544300	1.03263300
H	-1.85437400	3.03900300	0.02544800
H	-3.71681900	2.48543300	1.07940000
H	0.75756200	-0.40882100	-1.08222200
H	0.49655600	1.48063900	0.00014300
P	-2.88613300	2.02866600	0.01632900
P	-0.01103700	0.13372400	-0.01402800
Cl	0.27339500	3.80134300	0.02765200

RC: $Pd(PH_3)_2Cl^-$ + $(CH_3)_3C-H$

E = -2514.89
H = -2392.35
G = -2433.29
N_{imag} = 0

C	2.70435900	0.62757100	-4.52960000
H	2.72771300	0.33866700	-3.46292400
H	4.85716600	1.02120500	-4.58827700
Cl	-0.08277600	-1.71552800	-3.01680400
Pd	1.87246900	-1.00443300	-1.57333800
H	5.05895800	-1.76503900	-1.35979700
H	0.86543500	2.00148500	-0.51747500
H	2.77634200	1.61912300	0.16959800
H	4.25528000	-2.94329400	-2.85725000
H	3.92924300	-3.43429200	-0.86715900
H	1.14797700	0.71406600	1.08225000
P	3.75065600	-2.26679200	-1.68730700
P	1.65641700	0.82303500	-0.25771600

H 2.71948000 -0.46807800 -6.42272400
C 1.37007100 1.35458800 -4.76329400
H 0.53787400 0.70341500 -4.47166200
H 1.25406800 1.63288600 -5.82317600
H 1.31217900 2.27397200 -4.16371300
C 2.77112800 -0.67532500 -5.34183700
H 3.70800000 -1.21445700 -5.14417600
H 1.93702700 -1.32932800 -5.06328500
C 3.91251000 1.53830800 -4.80699900
H 3.93852100 1.85154300 -5.86283700
H 3.87510100 2.44662700 -4.18997400

TS: $Pd(PH_3)_2Cl^-$ + $(CH_3)_3C-H$

E = -2476.86
H = -2357.41
G = -2396.31
N_{imag} = 1, -773.1 cm^{-1}

C -4.66603200 -1.29370900 1.45234400
C -4.26860900 -1.49713400 -0.01643400
C -4.35780500 -3.00511500 -0.33660000
H -4.75891000 -0.23423800 1.70962200
H -5.64279000 -1.78295300 1.65165800
H -3.93175700 -1.74128300 2.13285600
H -2.67917600 -1.67795700 -0.40745100
H -4.08351400 -3.21557400 -1.37792400
H -3.69879600 -3.59707000 0.31077800
H -5.39451300 -3.36184800 -0.18112100
Pd -2.37721300 -0.13452100 -0.14573300
H -3.64112500 2.43361400 1.66294300
H 0.45490800 1.51195800 -0.25885400
H -3.78462000 2.90779800 -0.37899500
H -1.84443700 3.08114400 0.59311800
H 0.87609700 -0.59236200 0.11745600
H 0.43487000 0.02496100 -1.84386800
P -2.92696300 2.13591100 0.46137800
P -0.10188100 0.21528300 -0.53577200
C -5.22069400 -0.75018200 -0.96189300
H -4.87767300 -0.80225400 -2.00219100
H -5.31902300 0.30627600 -0.69426500
H -6.23078400 -1.21032900 -0.92369500
Cl 0.33162400 3.78676900 0.40292500

P: $Pd(PH_3)_2Cl^-$ + $(CH_3)_3C-H$

E = -2488.60
H = -2368.41
G = -2406.12
N_{imag} = 0

C -4.49704500 -2.01628800 1.26325700
C -4.33939800 -1.16728200 -0.00984600
C -4.49950000 -2.05943400 -1.25323900
H -4.35417400 -1.41546200 2.17067000

H	-5.51781900	-2.45245300	1.31305400
H	-3.77420200	-2.83816700	1.29148100
H	-1.87724000	-1.83953900	-0.05746800
H	-4.34765400	-1.49215700	-2.18050500
H	-3.78323100	-2.88730600	-1.24990100
H	-5.52394100	-2.48802300	-1.29161800
Pd	-2.33042800	-0.29271800	-0.01622100
H	-3.73579100	2.48472600	1.13097700
H	0.41970100	1.61145000	-0.01009200
H	-3.77273200	2.53720800	-0.98150200
H	-1.89454700	3.00510800	0.05493300
H	0.76413500	-0.31377900	1.00174600
H	0.73560400	-0.26769300	-1.11449600
P	-2.94095700	2.01352100	0.04806500
P	-0.03710000	0.24205000	-0.03419900
C	-5.45492100	-0.11054400	-0.02883900
H	-5.40934700	0.52123200	-0.92436400
H	-5.41871000	0.54460600	0.85013800
H	-6.44852100	-0.60457400	-0.02709400
Cl	0.21165300	3.87902300	0.04555500

Table S4. Cartesian coordinates (in Å), energies (*E*, *H* and *G*, in kcal mol^{-1}), and number of imaginary vibrational frequencies (N_{imag}) all stationary points of the C–C activation reactions in the gas phase, computed at ZORA-BLYP/TZ2P.

R: $H_3C–CH_3$
E = -896.24
H = -847.97
G = -864.88
N_{imag} = 0

C	0.00000000	0.00000000	0.76970900
H	0.00000000	1.02233700	1.16808800
H	0.88537000	-0.51116800	1.16808800
H	-0.88537000	-0.51116800	1.16808800
C	0.00000000	0.00000000	-0.76970900
H	-0.88537000	0.51116800	-1.16808800
H	0.88537000	0.51116800	-1.16808800
H	0.00000000	-1.02233700	-1.16808800

R: $CH_3H_2C–CH_3$
E = -1258.70
H = -1192.28
G = -1211.46
N_{imag} = 0

C	0.00000000	0.00000000	0.02375700
C	1.28541800	0.00000000	0.87229900
C	-1.28541800	0.00000000	0.87229900
H	0.00000000	0.87911600	-0.63672100
H	0.00000000	-0.87911600	-0.63672100
H	2.18284600	0.00000000	0.24110400
H	1.33169600	0.88606900	1.51967000
H	1.33169600	-0.88606900	1.51967000
H	-2.18284600	0.00000000	0.24110400
H	-1.33169600	-0.88606900	1.51967000
H	-1.33169600	0.88606900	1.51967000

R: $(CH_3)_2HC–CH_3$
E = -1621.51
H = -1537.28
G = -1558.89
N_{imag} = 0

C	0.00000000	0.00000000	0.35696000
C	1.47046700	0.00000000	-0.11187200
C	-0.73523300	-1.27346200	-0.11187200
C	-0.73523300	1.27346200	-0.11187200
H	0.00000000	0.00000000	1.45859800
H	2.00502500	0.88715800	0.25135400
H	2.00502500	-0.88715800	0.25135400
H	1.53017500	0.00000000	-1.20955500
H	-1.77081400	1.29282400	0.25135400
H	-0.23421100	2.17998200	0.25135400
H	-0.76508800	1.32517000	-1.20955500

H -1.77081400 -1.29282400 0.25135400
H -0.76508800 -1.32517000 -1.20955500
H -0.23421100 -2.17998200 0.25135400

R: $(CH_3)_3C-CH_3$
E = -1983.86
H = -1881.94
G = -1903.88
N_{imag} = 0
C 0.00000000 0.00000000 0.00000000
C -0.89431400 0.89431400 -0.89431400
C -0.89431400 -0.89431400 0.89431400
C 0.89431400 -0.89431400 -0.89431400
C 0.89431400 0.89431400 0.89431400
H -0.28612300 -1.54062500 1.54062500
H -1.54062500 -0.28612300 1.54062500
H -1.54062500 -1.54062500 0.28612300
H -0.28612300 1.54062500 -1.54062500
H -1.54062500 0.28612300 -1.54062500
H -1.54062500 1.54062500 -0.28612300
H 1.54062500 0.28612300 1.54062500
H 1.54062500 1.54062500 0.28612300
H 0.28612300 1.54062500 1.54062500
H 1.54062500 -1.54062500 -0.28612300
H 0.28612300 -1.54062500 -1.54062500
H 1.54062500 -0.28612300 -1.54062500

R: $CH_3H_2C-CH_2CH_3$
E = -1621.04
H = -1536.47
G = -1558.33
N_{imag} = 0
C 0.00179200 0.01317500 0.00000000
C 1.54316200 -0.00867500 0.00000000
C 2.13631200 -1.42972200 0.00000000
C -0.59119400 1.43430800 0.00000000
H 1.91079400 0.54113800 0.87977800
H 1.91079400 0.54113800 -0.87977800
H 3.23311700 -1.40547400 0.00000000
H 1.81473600 -1.99320200 0.88609300
H 1.81473600 -1.99320200 -0.88609300
H -0.36590400 -0.53657700 -0.87977800
H -0.36590400 -0.53657700 0.87977800
H -1.68798100 1.41032000 0.00000000
H -0.26948100 1.99772500 0.88608900
H -0.26948100 1.99772500 -0.88608900

R: $(CH_3)_2HC-CH(CH_3)_2$
E = -2343.21
H = -2222.59
G = -2248.33

N_{imag} = 0

C	0.77732500	0.04724400	0.05040100
H	-1.08186200	-0.21129100	-2.12745200
C	-0.77732500	-0.04724400	0.05040100
C	1.31801100	0.75602700	1.30963500
C	1.31799200	0.75600800	-1.20885100
H	2.40828500	0.85754900	1.25581100
C	-1.31799200	-0.75600800	-1.20885100
C	-1.31801100	-0.75602700	1.30963500
H	-0.89334600	-1.76580000	-1.29541200
H	0.89342500	1.76585300	1.39614700
H	1.08183500	0.21136300	2.22825600
H	0.89334600	1.76580000	-1.29541200
H	2.40825500	0.85759700	-1.15500900
H	1.08186200	0.21129100	-2.12745200
H	-2.40828500	-0.85754900	1.25581100
H	-0.89342500	-1.76585300	1.39614700
H	-1.08183500	-0.21136300	2.22825600
H	-2.40825500	-0.85759700	-1.15500900
H	1.16327700	-0.98589800	0.05040500
H	-1.16327700	0.98589800	0.05040500

R: $(CH_3)_3C–C(CH_3)_3$

E = -3061.44

H = -2905.28

G = -2934.12

N_{imag} = 0

C	1.56205800	0.02912700	-0.00002900
C	2.15749900	-1.40251900	-0.11128500
C	-0.04430400	-0.00264900	0.00002700
C	2.11605500	0.66564800	1.30545200
C	2.11252600	0.85794000	-1.19431100
C	-0.63955600	1.42909000	-0.11104900
C	-0.59498700	-0.83129800	-1.19427000
C	-0.59829800	-0.63918000	1.30550900
H	3.24999900	-1.35533700	-0.01948600
H	1.79303800	-2.06701500	0.68036300
H	1.93193000	-1.86850400	-1.07655100
H	-1.73206800	1.38202900	-0.01932600
H	-0.27502700	2.09339800	0.68073200
H	-0.41386500	1.89517900	-1.07623400
H	-1.68999700	-0.72762200	1.23795800
H	-0.20055200	-1.64561500	1.47845300
H	-0.37528800	-0.02952100	2.18772000
H	-1.68981400	-0.76051200	-1.21820200
H	-0.22429000	-0.46670900	-2.15902300
H	-0.33938400	-1.89317600	-1.11142700
H	1.71808000	1.67198600	1.47854800
H	1.89324300	0.05584000	2.18764100
H	3.20774100	0.75432300	1.23782100
H	1.85640100	1.91968500	-1.11160700

H	3.20737800	0.78766300	-1.21805200
H	1.74218500	0.49305200	-2.15907200

R: $Pd(PH_3)_2$

E = -756.21
H = -718.97
G = -743.55
N_{imag} = 0

Pd	0.00000000	0.00000000	0.00000000
P	0.00000000	0.00000000	2.28490600
H	0.60928100	1.05530600	3.01943100
H	0.60928100	-1.05530600	3.01943100
H	-1.21856200	0.00000000	3.01943100
P	0.00000000	0.00000000	-2.28490600
H	-1.21856200	0.00000000	-3.01943100
H	0.60928100	-1.05530600	-3.01943100
H	0.60928100	1.05530600	-3.01943100

R: $PdCl^-$

E = -119.61
H = -116.86
G = -134.51
N_{imag} = 0

Pd	0.00000000	0.00000000	-0.00847700
Cl	0.00000000	0.00000000	2.32586400

RC: Pd + H_3C–CH_3

E = -902.91
H = -853.86
G = -876.73
N_{imag} = 0

C	0.21136100	-2.77545300	0.00000000
C	0.87831800	-1.38788700	0.00000000
H	0.50501400	-3.35063200	-0.88698900
H	-0.88050100	-2.69114200	0.00000000
H	0.50501400	-3.35063200	0.88698900
H	1.97157200	-1.44682800	0.00000000
H	0.60141900	-0.82796200	-0.93569500
H	0.60141900	-0.82796200	0.93569500
Pd	-0.21921800	0.76689800	0.00000000

TS: Pd + H_3C–CH_3

E = -877.59
H = -830.66
G = -851.96
N_{imag} = 1, -491.46 cm^{-1}

Pd	0.00000000	0.00000000	-1.93577400
C	-0.97262500	0.00082000	-0.00067900
C	0.97262500	-0.00082000	-0.00067900
H	-1.74108200	0.24267600	-0.78129500

H	1.74108200	-0.24267600	-0.78129500
H	-1.04258900	0.78133400	0.75529400
H	-1.19760700	-0.97839500	0.41931700
H	1.04258900	-0.78133400	0.75529400
H	1.19760700	0.97839500	0.41931700

P: Pd + $H_3C{-}CH_3$

E = -904.97
H = -857.50
G = -880.04
N_{imag} = 0

Pd	0.00000000	0.00000000	-1.81399200
C	-1.50566200	0.04156800	-0.46171100
C	1.50566200	-0.04156800	-0.46171100
H	-2.40646600	0.06328900	-1.09312500
H	2.40646600	-0.06328900	-1.09312500
H	-1.43394500	0.94553300	0.14920300
H	-1.48059100	-0.86214700	0.15322900
H	1.43394500	-0.94553300	0.14920300
H	1.48059100	0.86214700	0.15322900

RC: Pd + $CH_3H_2C{-}CH_3$

E = -1265.51
H = -1198.33
G = -1223.46
N_{imag} = 0

C	0.03246100	-0.05236700	-0.03517200
H	0.39776600	0.98233300	-0.01997800
C	-1.50877800	-0.04770700	0.01214700
C	0.59200300	-0.78208100	-1.27032100
H	0.41447400	-0.53080400	0.87827300
H	-1.92823200	-1.05980500	0.03858600
H	-1.86428500	0.46271000	0.94887200
H	-1.91506300	0.42998400	-0.92417500
H	0.25909100	-1.82808900	-1.29798600
H	0.25828300	-0.30066100	-2.19895900
H	1.68903200	-0.78073300	-1.26735600
Pd	-2.99775300	1.85442100	0.00577000

TS: Pd + $CH_3H_2C{-}CH_3$

E = -1238.63
H = -1173.57
G = -1197.68
N_{imag} = 1, -475.13 cm^{-1}

C	0.04051200	-0.06590700	0.01655900
C	2.00643700	-0.01564100	0.10782400
H	-0.03229700	-0.69775400	0.90183900
C	-0.32745600	-0.81076600	-1.26886600
H	-0.70003900	0.76439300	0.18824100
H	2.78482800	0.77110700	-0.08162300
H	2.17436700	-0.43010000	1.10149500

H	2.13819600	-0.77147100	-0.66525800
H	-1.38107600	-1.12527900	-1.23546700
H	-0.19947900	-0.17943800	-2.15462800
H	0.28138300	-1.71354500	-1.40575200
Pd	1.00642200	1.89640100	-0.01416600

P: Pd + CH_3H_2C–CH_3

E = -1266.88
H = -1201.14
G = -1226.53
N_{imag} = 0

C	0.01867100	-0.01079800	0.01654600
C	3.10118300	-0.05932100	-0.08146700
H	0.07961300	0.53101600	-0.93365700
C	-0.08676900	0.92438800	1.21722000
H	-0.82050600	-0.72871300	-0.03011400
H	3.96453400	-0.69749300	-0.31969100
H	2.93710000	0.67500000	-0.87486700
H	3.22500900	0.42453700	0.89240400
H	-1.00301400	1.53397600	1.13763400
H	-0.13645100	0.37918800	2.16600300
H	0.76012800	1.61803100	1.26649500
Pd	1.54260300	-1.35571100	0.03179500

RC: Pd + $(CH_3)_2HC$–CH_3

E = -1626.93
H = -1541.94
G = -1569.05
N_{imag} = 0

C	0.50470600	0.20287100	0.74921300
C	1.88961400	0.45439100	1.37316400
C	-0.22235900	1.52158300	0.42850700
H	0.67509000	-0.34035100	-0.24645500
C	-0.35618900	-0.72107500	1.62992300
H	2.42163000	-0.48618500	1.55862100
H	2.51483900	1.07645100	0.72185300
H	1.78209000	0.97623100	2.33570900
H	-1.18874000	1.33822200	-0.05587700
H	-0.41111300	2.08461500	1.35465300
H	0.37583700	2.15725100	-0.23495500
H	-1.32423600	-0.93309000	1.16081400
H	0.14709200	-1.67661800	1.81898900
H	-0.55020300	-0.24422300	2.60224000
Pd	0.96744200	-1.23217200	-1.86699700

TS: Pd + $(CH_3)_2HC$–CH_3

E = -1600.08
H = -1517.09
G = -1542.89
N_{imag} = 1, -455.16 cm^{-1}

C	0.01541400	-0.05000200	0.12656500

C	2.02248400	-0.14509400	0.09128700
C	-0.15233400	1.45828000	0.33068100
H	-0.67784700	-0.33261800	-0.71489500
C	-0.38739400	-0.88022200	1.34910300
H	2.76974100	-0.82838100	-0.38498000
H	2.30253800	0.88730800	-0.11586600
H	2.05596900	-0.35535900	1.15982300
H	-1.20172600	1.68586300	0.57449400
H	0.46569300	1.82951000	1.15955900
H	0.11004300	2.02140000	-0.57106100
H	-1.45300500	-0.72051200	1.57545800
H	-0.23895400	-1.95183400	1.18212900
H	0.18345700	-0.59123300	2.24226400
Pd	1.00422000	-1.08910600	-1.56056100

P: Pd + $(CH_3)_2HC{-}CH_3$

E = -1629.72
H = -1546.09
G = -1573.92
N_{imag} = 0

C	0.00082900	-0.00336500	0.00000000
C	3.19449500	0.01012700	0.00000000
C	-0.13246200	0.82144400	-1.27773200
H	-0.73004900	-0.84018300	0.00000000
C	-0.13246200	0.82144400	1.27773200
H	4.07158000	-0.65354000	0.00000000
H	3.17536100	0.62546300	-0.90409000
H	3.17536100	0.62546300	0.90409000
H	-1.11594200	1.32324100	-1.29804300
H	0.63182000	1.60595200	-1.32769800
H	-0.05298100	0.20688800	-2.17983500
H	-1.11594200	1.32324100	1.29804300
H	-0.05298100	0.20688800	2.17983500
H	0.63182000	1.60595200	1.32769800
Pd	1.60495000	-1.26131500	0.00000000

RC: Pd + $(CH_3)_2HC{-}CH_3$

E = -1990.22
H = -1887.78
G = -1916.28
N_{imag} = 0

C	-0.05309100	-0.65990300	-0.12538000
C	1.48390000	-0.62617500	-0.27843100
C	-0.71765400	-0.24856300	-1.46138100
C	-0.50473800	-2.09354600	0.26089100
C	-0.46743600	0.32923900	0.98677300
H	-1.81206200	-0.25935300	-1.37807700
H	-0.40970300	0.76517800	-1.75159100
H	-0.43427800	-0.93346700	-2.27093100
H	1.99081500	-0.95966800	0.63473300
H	1.82972100	-1.24736900	-1.11431500

H   1.82830500   0.42608500 -0.48934400
H -1.55754000   0.41129900   1.08192800
H -0.05469500   0.04167000   1.96092900
H -0.08182100   1.36140700   0.74954900
H -1.59463200 -2.14368200   0.37900700
H -0.21806300 -2.81761700 -0.51231700
H -0.04790900 -2.41144100   1.20679700
Pd 1.56267700   2.37090800   0.07845900

TS: Pd + $(CH_3)_2HC–CH_3$
E = -1953.30
H = -1852.64
G = -1880.38
N_{imag} = 1, -405.97 cm^{-1}
C -0.22794600 -0.03986800 -0.07823600
C   1.87223900 -0.00358700   0.03021300
C -0.93413900   1.31106300 -0.29918600
C -0.20385800 -0.80107600 -1.41349000
C -0.91499900 -0.91865800   0.98160900
H -1.92398200   1.13474600 -0.76103400
H -1.09930900   1.86703600   0.63268800
H -0.36788600   1.95475300 -0.98089900
H   1.96125500 -1.08843600 -0.01401500
H   1.99858900   0.45290600 -0.95067300
H   2.71775100   0.37211300   0.68572200
H -1.90458300 -1.24012400   0.60644800
H -0.33586600 -1.82519100   1.18882900
H -1.07421200 -0.39560700   1.93277100
H -1.24653200 -0.98491400 -1.72205200
H   0.27834400 -0.23133000 -2.21528900
H   0.29113100 -1.77522000 -1.33678300
Pd 1.10350500   1.03829600   1.69607700

P: Pd + $(CH_3)_2HC–CH_3$
E = -1993.15
H = -1892.08
G = -1921.43
N_{imag} = 0
C   0.87120600   1.26175700   0.00000000
C   4.24033900   1.02916100   0.00000000
C   0.78002000   2.10538400 -1.26908700
C -0.00396600 -0.00277400   0.00000000
C   0.78002000   2.10538400   1.26908700
H   5.01825100   0.24850500   0.00000000
H   4.33855900   1.64537500 -0.89977300
H   4.33855900   1.64537500   0.89977300
H -0.19725800   2.62081900 -1.30430000
H   1.55774600   2.87632300 -1.28745200
H   0.87343700   1.50067200 -2.17672600
H -0.62701300 -0.08378200 -0.89785100
H -0.62701300 -0.08378200   0.89785100

H 0.61524300 -0.94587400 0.00000000
H -0.19725800 2.62081900 1.30430000
H 0.87343700 1.50067200 2.17672600
H 1.55774600 2.87632300 1.28745200
Pd 2.47114200 -0.00775900 0.00000000

RC: Pd + $CH_3H_2C{-}CH_2CH_3$
E = -1628.11
H = -1543.11
G = -1569.88
N_{imag} = 0
C 0.41275800 0.82245600 0.97489200
C 1.83798400 0.27244300 0.80000600
C -0.27481100 1.12729900 -0.36365300
C 2.51182800 -0.18372800 2.10371300
H -0.18755300 0.09433600 1.53903400
H 0.45041800 1.73770400 1.58584300
H 2.46581400 1.01907800 0.29442300
H 1.78358300 -0.61304100 0.09103300
H -1.33265900 1.38871200 -0.24130300
H 0.22280300 1.94204100 -0.90301300
H -0.23891300 0.21672800 -1.02835800
H 3.51571000 -0.58234900 1.91511300
H 2.61243000 0.65427600 2.80702400
H 1.92545300 -0.96793900 2.59951600
Pd 1.03565600 -1.27151600 -1.64426800

TS: Pd + $CH_3H_2C{-}CH_2CH_3$
E = -1599.55
H = -1516.32
G = -1542.19
N_{imag} = 1, -450.48 cm^{-1}
C -1.00832600 0.02041200 0.11428200
C 1.00832600 -0.02041200 0.11428200
C -1.38422200 1.29041300 0.88119500
C 1.38422200 -1.29041300 0.88119500
H -1.74132400 -0.12580100 -0.72741300
H -1.08782200 -0.87977600 0.72392200
H 1.08782200 0.87977600 0.72392200
H 1.74132400 0.12580100 -0.72741300
H -2.44689200 1.26414200 1.16467000
H -0.80058800 1.39924200 1.80449600
H -1.22825700 2.18996600 0.27546100
H 2.44689200 -1.26414200 1.16467000
H 0.80058800 -1.39924200 1.80449600
H 1.22825700 -2.18996600 0.27546100
Pd 0.00000000 0.00000000 -1.82035500

P: Pd + $CH_3H_2C{-}CH_2CH_3$
E = -1628.62
H = -1544.59

G = -1571.71
N_{imag} = 0

C	-0.10116200	-0.07064600	-0.11312600
C	3.08347600	-0.06632300	-0.08589600
C	-0.17945400	1.37692000	0.36271600
C	3.15606000	-0.65405900	1.32014900
H	-0.90303500	-0.31176500	-0.83419000
H	-0.13419700	-0.78626500	0.71682100
H	3.09782900	1.02996200	-0.08290200
H	3.89901900	-0.43928000	-0.73158300
H	-1.12733200	1.54798700	0.90076200
H	0.63283200	1.61979100	1.05776900
H	-0.13963800	2.08912700	-0.46842700
H	4.09301900	-0.34324100	1.81276800
H	2.33008400	-0.30297200	1.94970600
H	3.13568300	-1.74911100	1.31267400
Pd	1.50171600	-0.60432400	-1.25044100

RC: Pd + $(CH_3)_2HC\text{–}CH(CH_3)_2$

E = -2350.03
H = -2229.83
G = -2259.04
N_{imag} = 0

C	0.61623700	2.74744100	0.99453700
H	0.83544800	4.05359500	2.72824500
C	-0.93023500	2.62221400	1.02714300
C	1.22424900	3.09167700	2.36488200
C	1.12273300	3.72715500	-0.07736800
H	0.71467700	4.73324500	0.09518500
C	-1.49809700	2.24096700	-0.35151200
C	-1.39221600	1.58081900	2.06064400
H	1.00572000	2.32913700	3.11735700
H	2.31372900	3.17969100	2.28867500
H	-1.33563500	3.60662800	1.31596400
Pd	0.46261400	-0.14840900	0.34210100
H	2.21562300	3.80044100	-0.04234200
H	0.84322600	3.41511700	-1.08737400
H	1.00415300	1.71565300	0.70960500
H	-2.47686100	1.43401200	2.02138600
H	-0.91561100	0.59394700	1.81805900
H	-1.11315900	1.83623900	3.08646000
H	-2.57899100	2.07048900	-0.30012500
H	-1.30561100	2.99523700	-1.11963300
H	-1.02340000	1.28751900	-0.68229400

TS: Pd + $(CH_3)_2HC\text{–}CH(CH_3)_2$

E = -2315.47
H = -2196.18
G = -2225.61
N_{imag} = 1, -389.1 cm^{-1}

C	2.16625500	-0.00646800	-0.04008500
H	2.77829100	-0.93413400	-0.25078600
C	0.04576500	-0.01761100	0.03955200
C	2.50753700	0.36585700	1.39778000
C	2.60989800	1.05606400	-1.04725800
C	-0.26749500	1.41660200	-0.36978400
H	-0.59263200	-0.65773600	-0.64066800
C	-0.38507600	-0.33058700	1.47388700
H	3.70873200	1.12353100	-1.03859400
H	2.30253300	0.81259600	-2.06863300
H	2.22522500	2.05041000	-0.79915800
Pd	1.07778100	-1.68657100	-0.95738600
H	-1.34562300	1.59857700	-0.23987600
H	0.25994200	2.15091200	0.25182300
H	-0.02585000	1.61286800	-1.41783300
H	-1.48204400	-0.26727500	1.54200800
H	-0.09319800	-1.33844900	1.78369900
H	0.02313400	0.38393700	2.19574200
H	2.00620900	1.28745100	1.71898600
H	2.25653100	-0.42938200	2.10477100
H	3.59134900	0.54493700	1.47322500

P: Pd + $(CH_3)_2HC{-}CH(CH_3)_2$

E = -2353.32

H = -2233.33

G = -2264.36

N_{imag} = 0

C	-0.83601800	-0.12065800	0.13356500
C	2.44202900	-0.16639100	-0.22804000
H	-1.54640500	0.72649100	0.04188400
C	-0.98381500	-1.07126000	-1.04609900
C	-0.99033700	-0.80703400	1.48680000
C	2.53309000	-1.30813800	0.77386200
C	2.57966700	-0.62104600	-1.67702600
H	3.18947400	0.61820700	0.00856600
H	2.56258000	0.21804200	-2.37899100
H	1.77859800	-1.31566200	-1.95418100
Pd	0.83080700	1.06091200	0.04512900
H	-1.96982400	-1.31439700	1.54243500
H	-0.22296700	-1.57629400	1.63308600
H	-0.93030700	-0.10075700	2.32090600
H	-1.97223500	-1.56279400	-1.01907600
H	-0.88733300	-0.55790100	-2.00815800
H	-0.23063600	-1.86864200	-1.00460700
H	3.49818900	-1.83376500	0.66784300
H	2.44934700	-0.96118000	1.80826600
H	1.74491700	-2.05096700	0.59562700
H	3.53497900	-1.15838900	-1.81467600

RC: Pd + $(CH_3)_3C{-}C(CH_3)_3$

E = -3068.34

H = -2911.98
G = -2945.36
***N*_{imag}** = 0

C	1.86744800	0.71602000	0.40519200
C	2.46701500	-0.70224100	0.59252400
C	0.25696600	0.69743200	0.41086700
C	2.42018300	1.59195800	1.56729300
C	2.42154600	1.29586000	-0.92496400
C	-0.31788700	2.13681600	0.55629500
C	-0.32392100	0.11708400	-0.90512500
C	-0.29432800	-0.15011300	1.58994500
H	3.55640400	-0.66992300	0.47428500
H	2.24559200	-1.12605100	1.57658700
H	2.09294700	-1.43380600	-0.17796500
Pd	1.09379300	-2.56930400	-1.44555700
H	-1.40912500	2.11094800	0.44540400
H	-0.10055200	2.56719000	1.53955600
H	0.07211100	2.81883700	-0.20796700
H	-1.38599600	-0.05012700	1.63922800
H	-0.06935900	-1.21532800	1.46732900
H	0.10664900	0.17580800	2.55661300
H	-1.41264300	0.01694100	-0.82505500
H	-0.10450700	0.74416400	-1.77436600
H	0.06662300	-0.91509800	-1.13089700
H	2.18915300	2.65303800	1.42591600
H	2.02611300	1.28438200	2.54272000
H	3.51248900	1.49899200	1.61176100
H	2.00823800	2.28612100	-1.14859700
H	3.51123400	1.40525000	-0.85568800
H	2.21311300	0.63755000	-1.77553400

TS: Pd + $(CH_3)_3C–C(CH_3)_3$
E = -3018.36
H = -2864.81
G = -2898.88
N_{imag} = 1, -253.64 cm^{-1}

C	2.24756300	-0.00333500	0.03205700
C	3.08779700	-1.30640300	0.07343400
C	-0.10579900	-0.00789300	-0.01748100
C	2.44421200	0.69538800	1.37524500
C	2.65666600	0.88621300	-1.13903200
C	-0.32491900	1.49958900	-0.12219600
C	-0.93571600	-0.66303500	-1.15415900
C	-0.51275800	-0.55163800	1.34984300
H	4.16298800	-1.03821400	0.04559500
H	2.92639200	-1.88215600	0.98999900
H	3.00921100	-2.00812400	-0.82042600
Pd	1.08582400	-1.88878800	-1.07003200
H	-1.40765700	1.69938200	-0.02189100
H	0.17492400	2.07270900	0.66249400
H	-0.02192900	1.89969100	-1.09533500

H	-1.59600300	-0.39122500	1.50102500
H	-0.32774300	-1.62804800	1.43981400
H	-0.00009000	-0.04301700	2.17128200
H	-2.01124000	-0.58102200	-0.90053500
H	-0.79529500	-0.16465900	-2.11819300
H	-0.83032700	-1.78924700	-1.30331200
H	1.93054000	1.65696200	1.45093800
H	2.14276600	0.06948400	2.22151400
H	3.52322600	0.90024800	1.50294800
H	2.12787900	1.84377500	-1.14085500
H	3.73539300	1.11716500	-1.06552800
H	2.49329800	0.39900000	-2.10701000

P: Pd + $(CH_3)_3C–C(CH_3)_3$

E = -3078.49
H = -2923.10
G = -2958.42
N_{imag} = 0

C	-0.95417300	-0.11840800	-0.00539000
C	2.51133400	-0.31305900	-0.09734000
C	-1.86547700	1.12264100	-0.10950900
C	-1.10067500	-1.03594300	-1.21893800
C	-1.18281400	-0.87722500	1.30488200
C	2.54065500	-1.40937800	0.96702500
C	2.66348700	-0.89247400	-1.50668600
C	3.55708600	0.78713400	0.18078500
H	-2.92385900	0.81177900	-0.03776300
H	-1.70026700	1.83653100	0.70998100
H	-1.74888600	1.64843500	-1.06452700
H	-2.20065800	-1.31102100	1.31389500
H	-0.47757900	-1.70708200	1.42258500
H	-1.09277500	-0.22695800	2.18190500
H	-2.12454300	-1.45316000	-1.25428300
H	-0.92449300	-0.50761100	-2.16206100
H	-0.41094400	-1.88615100	-1.16826000
H	3.51016400	-1.94092900	0.93354600
H	2.41618100	-1.00869400	1.97875600
H	1.75951500	-2.15900200	0.79656400
H	3.62415300	-1.43559700	-1.58677800
H	1.86790100	-1.60829500	-1.74013000
H	2.65838000	-0.11439800	-2.27785600
H	4.57366900	0.37474600	0.04495000
H	3.47519200	1.63250000	-0.51742100
H	3.49894500	1.16989100	1.20645800
Pd	0.84458500	0.92092500	0.03040900

RC: $Pd(PH_3)_2$ + $H_3C–CH_3$

E = -1652.18
H = -1566.65
G = -1600.14
N_{imag} = 1, -36.48 cm^{-1}

Pd	0.00000000	0.00000000	-0.94625800
C	0.13411700	0.75747900	3.63477000
C	-0.13411700	-0.75747900	3.63477000
H	0.69225500	1.05978700	2.74026600
H	-0.80376100	1.32723100	3.64675900
H	0.71648900	1.06108700	4.51399500
H	-0.69225500	-1.05978700	2.74026600
H	0.80376100	-1.32723100	3.64675900
H	-0.71648900	-1.06108700	4.51399500
P	-1.92926900	1.22429200	-0.97845000
P	1.92926900	-1.22429200	-0.97845000
H	1.97048700	-2.54677000	-0.45539600
H	-2.57098100	1.53596300	-2.20920100
H	2.57098100	-1.53596300	-2.20920100
H	3.10353100	-0.77172800	-0.31461400
H	-3.10353100	0.77172800	-0.31461400
H	-1.97048700	2.54677000	-0.45539600

TS: Pd(PH$_3$)$_2$ + H$_3$C–CH$_3$
E = -1600.62
H = -1516.49
G = -1549.67
N_{imag} = 1, -444.77 cm^{-1}

Pd	0.00000000	0.00000000	-1.97791800
C	0.00819100	1.05268000	-0.00289400
C	-0.00819100	-1.05268000	-0.00289400
H	0.41946800	1.83119900	-0.66774700
H	-0.99690300	1.30978000	0.33030000
H	0.68317500	0.93838200	0.84370600
H	-0.41946800	-1.83119900	-0.66774700
H	0.99690300	-1.30978000	0.33030000
H	-0.68317500	-0.93838200	0.84370600
P	-1.63804200	1.10273600	-3.29662900
P	1.63804200	-1.10273600	-3.29662900
H	2.43408700	-2.20752600	-2.85866700
H	-1.40350600	1.66341800	-4.58872000
H	1.40350600	-1.66341800	-4.58872000
H	2.75400300	-0.32461100	-3.72173900
H	-2.75400300	0.32461100	-3.72173900
H	-2.43408700	2.20752600	-2.85866700

P: Pd(PH$_3$)$_2$ + H$_3$C–CH$_3$
E = -1625.40
H = -1540.30
G = -1573.18
N_{imag} = 0

Pd	0.00000000	0.00000000	0.01991300
C	-1.02106600	0.98675400	1.61424800
C	1.02106600	-0.98675400	1.61424800
H	-1.52391600	1.90824800	1.29702000
H	-1.76762700	0.28219300	1.99813600

```
H  -0.30285300   1.22472800   2.40342700
H   1.52391600  -1.90824800   1.29702000
H   1.76762700  -0.28219300   1.99813600
H   0.30285300  -1.22472800   2.40342700
P  -1.28601900   1.27424000  -1.48368400
P  1.28601900  -1.27424000  -1.48368400
H   1.14097200  -2.68890100  -1.45792800
H  -1.28295600   1.14843600  -2.90426100
H   1.28295600  -1.14843600  -2.90426100
H   2.69981700  -1.26413800  -1.33311400
H  -2.69981700   1.26413800  -1.33311400
H  -1.14097200   2.68890100  -1.45792800
```

RC: $Pd(PH_3)_2$ + $CH_3H_2C–CH_3$

E = -2014.86
H = -1911.15
G = -1946.99
N_{imag} = 1, -12.66 cm^{-1}

```
Pd -2.29295500   0.20554300   0.64808800
C  -2.66180000   0.59973300  -4.96138900
C  -2.33819800  -0.60451500  -5.86445200
H  -2.60862400   0.32246200  -3.90041100
H  -1.95958300   1.42616700  -5.12752500
H  -3.67460500   0.97754400  -5.15644500
H  -1.30933000  -0.93975700  -5.66879400
C  -3.30495200  -1.78634800  -5.66662800
H  -2.36140900  -0.28828400  -6.91744300
H  -5.51895500   0.22753500   0.20411000
H   0.62838200  -0.87423300   1.59448600
H  -5.08583500   0.99492700   2.12207600
H  -4.84339800   2.21587400   0.41729700
H   0.86792100   0.33502800  -0.11887600
H   0.19758700  -1.65627000  -0.31802900
P  -0.13099900  -0.50393900   0.44952600
P  -4.45451800   0.91709900   0.84927800
H  -3.04861200  -2.62899100  -6.32098400
H  -3.28066800  -2.14787200  -4.63000400
H  -4.33925100  -1.49220100  -5.89048100
```

TS: $Pd(PH_3)_2$ + $CH_3H_2C–CH_3$

E = -1961.73
H = -1859.51
G = -1895.39
N_{imag} = 1, -416.72 cm^{-1}

```
Pd  1.05369600   1.96359400  -0.00727200
C   2.15516500   0.01601900   0.01844400
C   0.02003800  -0.05619400   0.05081500
H   2.91713500   0.67845000  -0.42725100
H   2.43335100  -0.27062400   1.03319000
H   2.05682800  -0.86113800  -0.61888100
H  -0.69617800   0.66237600   0.49007300
```

C	-0.49523400	-0.65988100	-1.25021300
H	0.20319600	-0.81862100	0.80829400
H	-1.04791000	2.92119500	-2.53243100
H	2.70781900	4.58009100	1.40507000
H	-0.61259200	4.57658900	-1.34594400
H	0.82861600	3.83812700	-2.66760700
H	1.34354500	3.73660500	2.74331800
H	3.22083300	2.85198900	2.45405400
P	2.13094600	3.29475500	1.63986000
P	-0.00505000	3.31736700	-1.63530800
H	-1.37210200	-1.29656100	-1.05048700
H	-0.79802700	0.10622200	-1.97103000
H	0.25472700	-1.29755900	-1.73559500

P: Pd(PH$_3$)$_2$ + CH$_3$H$_2$C–CH$_3$

E = -1985.37
H = -1882.65
G = -1916.54
N_{imag} = 1, -28.33 cm^{-1}

C	0.05430100	-0.10146100	0.05210300
C	2.91333000	0.00037900	0.14885400
Pd	1.44179500	1.54398800	0.02304900
H	0.52053200	-0.89411800	0.64501100
H	-0.84734700	0.22222400	0.59100700
C	-0.30923600	-0.60660000	-1.35093200
H	2.89595600	-0.37524600	1.17853200
H	3.92671100	0.34663300	-0.08867700
H	2.64544000	-0.80806400	-0.53786500
P	-0.40387600	3.00645200	-0.03575000
P	3.21098200	3.11318300	-0.02604700
H	-1.12398300	3.21925000	1.17318600
H	-1.53628800	2.66145800	-0.82388600
H	-0.36199600	4.37797700	-0.42156500
H	3.08980000	4.53469600	-0.02589100
H	4.19538300	3.04676700	0.99778400
H	4.12232600	3.04325900	-1.11552000
H	-1.02107100	-1.44763200	-1.29767700
H	-0.77969000	0.17138000	-1.96693000
H	0.57263300	-0.96412600	-1.89648600

RC: Pd(PH$_3$)$_2$ + (CH$_3$)$_2$HC–CH$_3$

E = -2377.66
H = -2256.69
G = -2292.20
N_{imag} = 1, -28.68 cm^{-1}

Pd	2.26958500	-0.09458400	-0.17907700
C	3.11983700	-0.14745000	5.28984800
C	2.07001200	-1.05378400	4.61458700
H	2.91147700	0.91344400	5.10088500
H	4.13020200	-0.36275500	4.91882700
H	3.12552100	-0.29831500	6.37885600

H	2.10725500	-0.85965300	3.53111900
C	0.64617400	-0.71591900	5.10393800
C	2.39595900	-2.54528500	4.83654800
H	-0.92926300	0.20237200	0.36331600
H	5.19593400	0.73287400	-1.32117500
H	-0.62385700	-0.59597400	-1.56713400
H	-0.46518000	-1.84293300	0.12875600
H	5.45853500	-0.49173100	0.37810800
H	4.96753000	1.54728300	0.61318600
P	4.49316900	0.42582800	-0.12288500
P	0.04293100	-0.58842400	-0.31038700
H	-0.10629100	-1.33678600	4.60073700
H	0.39768000	0.33573500	4.91183200
H	0.55227800	-0.88946600	6.18547100
H	1.67177100	-3.19242400	4.32525800
H	2.37166800	-2.79668200	5.90659500
H	3.39507500	-2.79577100	4.45749100

TS: $Pd(PH_3)_2$ + $(CH_3)_2HC\text{–}CH_3$

E = -2322.40
H = -2202.92
G = -2239.29
N_{imag} = 1, -363.47 cm^{-1}

Pd	1.09339600	1.99442300	-0.00044300
C	2.11828400	0.02058200	0.07600200
C	-0.09636900	0.00895100	-0.05000700
H	2.88371200	0.63877000	0.57372800
H	2.44117700	-0.27418800	-0.92185000
H	1.93178100	-0.85260500	0.69793400
H	-0.74593100	0.85567700	-0.33797200
C	-0.55554600	-0.57352900	1.28165500
C	-0.10928600	-1.00193000	-1.19419200
H	-0.85017100	3.11739300	2.60877100
H	2.89690300	4.44843300	-1.48793900
H	-0.44143600	4.70656800	1.32849900
H	1.05910300	3.97263000	2.58407600
H	1.45602900	3.68766600	-2.79424800
H	3.26599700	2.67106500	-2.51313000
P	2.22316400	3.20609900	-1.69296900
P	0.14431200	3.43984200	1.63012800
H	-1.55427200	-1.02664600	1.16390300
H	-0.62305200	0.18584900	2.06635800
H	0.11072100	-1.37061500	1.63751300
H	-1.13738000	-1.37376000	-1.33815000
H	0.52299300	-1.87633200	-0.99315800
H	0.21347100	-0.55364300	-2.14000900

P: $Pd(PH_3)_2$ + $(CH_3)_2HC\text{–}CH_3$

E = -2345.81
H = -2224.51
G = -2261.71

N

N $_{imag}$ = 0

C	0.13702800	-2.17005900	-0.24992200
C	2.15364900	-0.14004600	0.00521700
Pd	0.01761200	-0.01557400	-0.00676900
H	1.21075100	-2.37391000	-0.29037600
C	-0.44620600	-2.90570600	0.96489900
C	-0.48736100	-2.63027300	-1.57504500
H	2.44961600	-0.80443200	0.82403500
H	2.65269000	0.82809600	0.13749300
H	2.47079200	-0.57348900	-0.94939500
P	-2.34402200	0.02393800	-0.04087900
P	0.29727500	2.33133300	0.26031700
H	-3.05492100	0.66664800	1.01363000
H	-3.01505400	0.71019200	-1.09385900
H	-3.16376700	-1.13844900	-0.08042400
H	-0.71234000	3.33681800	0.36264200
H	1.05235000	2.77175700	1.38179400
H	1.07417000	3.00724600	-0.72026900
H	-0.33713800	-3.71642600	-1.71317400
H	-1.57198200	-2.45877200	-1.61730300
H	-0.03627100	-2.12662100	-2.43827800
H	-0.30383900	-3.99650800	0.85821400
H	-1.52624600	-2.74215300	1.08282300
H	0.04041400	-2.60330900	1.89983000

RC: $Pd(PH_3)_2$ + $(CH_3)_3C–CH_3$

E = -2740.03

H = -2601.38

G = -2638.48

N $_{imag}$ = 1, -46.46 cm^{-1}

C	2.47029300	-5.39334800	2.30300400
C	2.88165200	-4.15230700	3.13400100
C	3.25616000	-5.40388700	0.96803100
C	2.79208700	-6.68086700	3.10238700
C	0.95046200	-5.33596500	2.00697500
H	2.98432200	-6.27451900	0.35659700
H	3.04660900	-4.50092100	0.37954600
H	4.33898100	-5.44474900	1.14739800
H	2.33941000	-4.11866000	4.08818700
H	3.95652300	-4.16692500	3.35929700
H	2.66430700	-3.22315100	2.59136400
H	0.62960600	-6.20558400	1.41816300
H	0.36680300	-5.32687400	2.93704900
H	0.69284200	-4.43175600	1.43949800
H	2.50938200	-7.57881400	2.53687500
H	3.86521200	-6.74907100	3.32633800
H	2.24802300	-6.70016800	4.05605100
Pd	2.10269400	0.89211100	-0.26982600
H	-0.92730800	2.05541300	-0.03912300
H	5.08752800	2.18250400	-0.38625400
H	-0.95476400	0.30859400	-1.22565000

H	-0.85574500	0.15423200	0.87820100
H	5.08006500	0.37941700	-1.48329800
H	5.18360900	0.33191800	0.62414700
P	4.38411400	0.94614400	-0.37945800
P	-0.17926600	0.85103100	-0.16250100

TS: $Pd(PH_3)_2$ + $(CH_3)_3C{-}CH_3$

E = -2676.85

H = -2538.91

G = -2578.73

N_{imag} = 1, -295.17 cm^{-1}

C	-1.47152200	-2.04047600	-0.04010000
C	0.73332100	-1.93632700	0.55167800
C	-2.25723400	-1.23997200	-1.09199200
C	-1.21160300	-3.44270900	-0.59386100
C	-2.20436100	-2.09721400	1.29909100
H	-3.21695600	-1.76480900	-1.26486200
H	-2.50343100	-0.21943300	-0.78006800
H	-1.74013200	-1.19865500	-2.05602000
H	0.40746000	-2.59871400	1.35004100
H	1.14415200	-2.49186400	-0.28895500
H	1.48274100	-1.23540200	0.96089800
H	-3.14126300	-2.66913000	1.17329300
H	-1.61964500	-2.60803200	2.07227500
H	-2.46996100	-1.10366400	1.67030800
H	-2.18452000	-3.91438800	-0.81432800
H	-0.64308400	-3.41830700	-1.53027500
H	-0.68867900	-4.09501500	0.11288600
Pd	-0.05395200	0.09370600	0.04605300
H	-1.84276100	1.46077400	2.60863900
H	2.14618100	1.89980600	-1.68826600
H	-1.27993500	2.96519900	1.28762900
H	0.14268500	2.11391500	2.55890900
H	0.48144500	1.52768100	-2.89257700
H	1.97893100	0.08558500	-2.69766300
P	1.16307900	0.87404300	-1.82248200
P	-0.82395400	1.65220100	1.61835100

P: $Pd(PH_3)_2$ + $(CH_3)_3C{-}CH_3$

E = -2703.02

H = -2563.82

G = -2601.59

N_{imag} = 0

C	-1.65857500	-1.68387200	0.34357200
C	1.26421100	-1.79493700	-0.44519000
C	-3.05868100	-1.03719400	0.35631100
C	-1.67865500	-2.80337500	-0.71163500
C	-1.36186600	-2.25261400	1.74216300
H	-3.82160100	-1.81040800	0.56357000
H	-3.17190200	-0.27955800	1.14255400
H	-3.32058700	-0.58489500	-0.60815200

H 1.04973500 -2.60470800 0.25452300
H 1.11505300 -2.14240000 -1.47368500
H 2.31384600 -1.50078100 -0.31852300
H -2.13654800 -2.99209500 2.02192100
H -0.39468900 -2.76367500 1.79287500
H -1.36810500 -1.47130300 2.51308000
H -2.48631100 -3.52072400 -0.47390000
H -1.88132400 -2.41139700 -1.71654700
H -0.74741000 -3.37398600 -0.75300700
Pd -0.08750100 -0.16280700 -0.12037400
H -2.05160300 1.93416500 1.58281700
H 1.82735500 2.58468200 -0.86497200
H -2.66084500 2.00042500 -0.43939400
H -0.91645800 3.02030500 0.17376700
H 2.46049100 0.85414400 -1.91320500
H 2.95074400 1.09025000 0.13120800
P 1.79655800 1.16676200 -0.69619700
P -1.47773200 1.71789500 0.29892200

RC: $Pd(PH_3)_2$ + $CH_3H_2C{-}CH_2CH_3$
E = -2376.95
H = -2256.86
G = -2289.23
N_{imag} = 1, -49.40 cm^{-1}
Pd 2.28501300 0.06335600 0.04250700
C 2.67937800 -4.57650900 -2.26005300
C 2.13839100 -3.76135000 -3.45033900
H 2.26763900 -4.16445800 -1.32694100
C 4.21622900 -4.59195900 -2.17590500
H 2.30734100 -5.60993700 -2.33151600
H 2.50174700 -2.72613300 -3.37051800
C 0.60184700 -3.75655200 -3.54112200
H 2.55819700 -4.16452900 -4.38455200
H -0.72931300 0.59937200 -1.06325900
H 5.31425300 0.67878000 1.07908900
H -0.71378600 -1.19726400 0.04436300
H -0.75909100 0.65910300 1.04609500
H 5.30259200 0.64424000 -1.03097700
H 5.29630000 -1.16610400 0.05422100
P 4.56994900 0.05698000 0.03793600
P 0.00037300 0.03281400 0.01866200
H 0.25354200 -3.16457300 -4.39657500
H 0.15434100 -3.32845700 -2.63452800
H 0.20746800 -4.77485300 -3.65730700
H 4.56384800 -5.18006600 -1.31750400
H 4.65950700 -5.02846600 -3.08085200
H 4.61593300 -3.57513600 -2.06782500

TS: $Pd(PH_3)_2$ + $CH_3H_2C{-}CH_2CH_3$
E = -2322.73
H = -2202.29

G = -2240.04
***N*$_{imag}$** = 1, -389.47 cm^{-1}

Pd	1.21306000	1.98803300	-0.01138000
C	2.20864400	-0.05058300	-0.02654900
C	0.04510100	0.04510100	-0.01889300
H	2.94915600	0.63344900	0.42797000
C	2.69749600	-0.61523400	-1.35567500
H	2.02224300	-0.83687800	0.70473600
H	-0.63051300	0.79544400	-0.47038400
C	-0.49441300	-0.48412200	1.30502000
H	0.16288400	-0.74936700	-0.75539400
H	-0.68297500	3.05325300	2.63906900
H	2.84634000	4.59027900	-1.41616800
H	-0.25779700	4.67525500	1.40467600
H	1.23391300	3.89109800	2.63973400
H	1.38334100	3.83046100	-2.69918900
H	3.26463900	2.92145900	-2.58856300
P	2.23792200	3.32464600	-1.67606300
P	0.31441600	3.39406600	1.67010200
H	-1.39597100	-1.09384400	1.13266000
H	-0.76604400	0.32224400	1.99387800
H	0.22914300	-1.13131900	1.81857800
H	3.53982600	-1.30603200	-1.18977400
H	1.91817800	-1.18909100	-1.87477900
H	3.04160800	0.16928200	-2.03741200

P: $Pd(PH_3)_2$ + CH_3H_2C–CH_2CH_3

E = -2345.18
H = -2224.14
G = -2260.23
***N*$_{imag}$** = 0

Pd	1.20877600	1.83611700	-0.01501800
C	2.38629700	0.18044700	-0.73126900
C	-0.12740400	0.29920500	0.69047500
H	3.44710200	0.46367700	-0.67571900
C	2.01911800	-0.24060000	-2.16118400
H	2.23658600	-0.64634700	-0.02963000
H	-1.15474300	0.68922600	0.65454700
C	0.20925000	-0.18745300	2.10722500
H	-0.07149800	-0.52342000	-0.02979600
H	-0.91293800	3.22969100	2.08960000
H	4.06962100	3.42968400	-0.01770600
H	-1.56703400	3.56790400	0.10908700
H	-0.10497000	4.82984500	0.96530900
H	2.74873300	4.67892100	-1.09177400
H	3.57613300	2.96494000	-2.01937700
P	2.89705100	3.28821200	-0.81214700
P	-0.33393000	3.43100300	0.80602500
H	-0.48659100	-0.97882800	2.43342200
H	0.14676300	0.61926800	2.84965700
H	1.22216200	-0.60501800	2.16579400

H   2.63474500 -1.09205300 -2.49729500
H   0.96977400 -0.55180400 -2.23803000
H   2.17169700   0.57008200 -2.88609600

RC: Pd(PH$_3$)$_2$ + (CH$_3$)$_2$HC–CH(CH$_3$)$_2$

E = -3095.79
H = -2937.17
G = -2976.87
N_{imag} = 0

C   0.77069500   3.55731800 -0.16308600
H   1.22247800   2.62746900   0.21560100
C -0.70576900   3.52432900   0.33198700
C   1.59180100   4.73852100   0.38869500
C   0.85582200   3.50630700 -1.70033000
C -1.52287400   4.77382800 -0.04823500
H -1.16001400   2.65797600 -0.17390300
C -0.79203400   3.25683000   1.84648300
H   2.60493300 -1.24438900 -1.73690400
H   1.71988700   0.29451100 -2.88269600
H -1.73453200 -0.08876300   2.58130800
Pd -0.01508200 -0.11317000 -0.17063000
H -2.57072700   4.65129000   0.25391400
H -1.13975800   5.67105700   0.45448400
H -1.50988400   4.96003500 -1.12825100
H -1.83311600   3.09818200   2.15569700
H -0.21572300   2.36365600   2.11745600
H -0.40384400   4.10143400   2.42948200
H -2.64721400 -1.42950200   1.22661900
H -2.93367100   0.65381100   1.01142300
H   2.92016600   0.77951400 -1.21722200
H   1.21229700   5.69839900   0.01633100
H   1.57843100   4.77199600   1.48400800
H   2.63945100   4.65572400   0.07293200
H   0.47472300   4.42808700 -2.15736100
H   1.89560400   3.38443400 -2.02984700
H   0.27167800   2.66578900 -2.09482200
P   1.82427700 -0.07691300 -1.51454900
P -1.84986000 -0.25271600   1.17385500

TS: Pd(PH$_3$)$_2$ + (CH$_3$)$_2$HC–CH(CH$_3$)$_2$

E = -3038.33
H = -2883.27
G = -2922.26
N_{imag} = 1, -256.80 cm^{-1}

C   1.55222800 -1.70491100   0.61643700
H   1.42452700 -2.12172100 -1.49633800
H   4.78830600 -0.63039000 -2.71032000
H   2.21234900 -5.34179400   0.64009100
C   4.46422100 -1.53336500 -0.74825300
H   4.44148100   0.54530800 -1.42730400
Pd 3.23814600 -3.20941000 -1.75759000

H	0.84824000	0.25710400	-1.24914400
H	5.05454300	-2.44731400	-0.96189400
C	2.01990600	-1.48622700	-0.81088900
C	1.90737100	-0.05054800	-1.27933200
C	4.93392200	-0.40622100	-1.64998600
H	2.45576100	0.64723600	-0.63515400
H	2.25555000	0.07834900	-2.30842400
H	0.47714900	-1.46474000	0.68257400
H	1.67762500	-2.74087600	0.94308200
H	2.06171300	-1.05270200	1.33233200
H	4.04351000	-0.31848300	1.01773000
H	4.23879500	-2.05389900	1.35498400
H	5.64799100	-1.05121400	0.97555500
H	6.01407400	-0.24616200	-1.49112200
C	4.58678700	-1.22650200	0.72961700
H	3.93102700	-6.05258600	-0.31056500
H	2.05987900	-6.29026800	-1.20593300
H	2.57171600	-3.52429000	-4.95728200
H	4.12326100	-2.13269000	-4.83392500
P	2.82387800	-5.22072700	-0.64932800
H	4.53899900	-4.16614000	-4.67562700
P	3.64384500	-3.23983200	-4.06169800

P: $Pd(PH_3)_2$ + $(CH_3)_2HC{-}CH(CH_3)_2$

E = -3060.82
H = -2902.98
G = -2943.29
N_{imag} = 0

C	1.48642700	1.82818700	-0.74413700
H	2.41413700	1.24591800	-0.85571400
C	-1.38279300	1.77700400	0.77735500
C	1.81713700	2.96799800	0.21772200
C	1.07520800	2.31454400	-2.13719900
C	-1.73829100	2.93916200	-0.14818700
H	-2.29920500	1.17502900	0.87510300
C	-0.97500800	2.22701900	2.18362900
H	2.15264500	-0.99496600	-2.17203500
H	1.40586500	-2.63043400	-1.03964400
H	-1.25932600	-2.69065000	0.87055400
Pd	0.06370000	0.30147000	-0.01235000
H	-2.56591900	3.52972100	0.28381300
H	-0.90318200	3.62894600	-0.29955600
H	-2.06484800	2.58825900	-1.13414700
H	-1.75139400	2.87816200	2.62453300
H	-0.83792500	1.37468300	2.86060300
H	-0.04125700	2.79749900	2.17988000
H	-1.99089800	-1.12056600	2.10066700
H	2.88241400	-1.35201900	-0.21885900
H	-2.74488700	-1.36878000	0.14006900
H	0.96902300	3.63782200	0.38614800
H	2.14689300	2.59354300	1.19378400

H	2.63527800	3.58595100	-0.19331600
H	0.12879300	2.86376700	-2.11993600
H	1.83879800	2.99684400	-2.55242100
H	0.96053700	1.48212900	-2.84259900
P	1.62460300	-1.23115300	-0.87319200
P	-1.47937500	-1.28472600	0.78437000

RC: $Pd(PH_3)_2$ + $(CH_3)_3C–C(CH_3)_3$

E = -3817.65

H = -3626.03

G = -3664.54

N_{imag} = 1, -35.47 cm^{-1}

C	0.76460400	4.79759200	0.10596800
C	1.27756500	3.57899700	0.92305000
C	-0.83196000	4.75480200	-0.06936300
C	1.21868900	6.08441600	0.85049900
C	1.48644600	4.77492200	-1.27031300
C	-1.36964700	6.07541700	-0.68791900
C	-1.26856900	3.58999800	-1.00161800
C	-1.54637300	4.55288100	1.29619800
H	2.37481400	3.58545000	0.94535300
H	0.93098300	3.60453500	1.96182000
H	0.96695300	2.62386800	0.48475300
Pd	0.16484300	-3.72397900	0.04727100
H	-2.44608100	5.98114500	-0.88018100
H	-1.23262300	6.93037900	-0.01705400
H	-0.88825900	6.31295500	-1.64351600
H	-2.63295100	4.62092100	1.15762300
H	-1.33468200	3.56970500	1.73001700
H	-1.26370700	5.31614900	2.03017000
H	-2.36406700	3.52971500	-1.02779800
H	-0.92611400	3.73664800	-2.03166000
H	-0.89779000	2.61882200	-0.65512500
H	1.02626500	6.98933400	0.26407700
H	0.72527200	6.19813700	1.82256700
H	2.29939500	6.04041000	1.03694400
H	1.15287500	5.58563600	-1.92801700
H	2.56675400	4.89932600	-1.12229300
H	1.33749600	3.82664300	-1.79779300
P	0.34986100	-3.76361800	-2.22948300
P	-0.02262400	-3.69062200	2.32299600
H	-0.30405600	-2.48250100	3.02014300
H	1.55334300	-4.18450400	-2.86148500
H	-1.00773300	-4.47218400	2.98915000
H	1.06081300	-4.08706400	3.15592600
H	-0.51630300	-4.56711900	-3.02241500
H	0.19086700	-2.57841400	-3.00069400

TS: $Pd(PH_3)_2$ + $(CH_3)_3C–C(CH_3)_3$

E = -3743.96

H = -3553.82

G = -3597.89
***N*_{imag}** = 1, -126.37 cm^{-1}

C	1.52179100	2.40420500	0.10915200
C	2.46336500	1.37901400	0.71636800
C	-1.48230500	2.32647100	-0.08338900
C	1.39659600	3.61387300	1.00063700
C	1.80138000	2.69929800	-1.34423800
C	-1.42254600	3.55954700	-0.94912500
C	-2.36210200	1.26254500	-0.71558700
C	-1.78078100	2.57565300	1.37470700
H	3.48649600	1.81510900	0.71880500
H	2.21889300	1.14827900	1.75784400
H	2.51794100	0.44171200	0.15028400
Pd	0.09806000	-0.24650300	-0.00421500
H	-2.44635100	3.97668300	-1.03622200
H	-0.79759100	4.35962300	-0.54484000
H	-1.09539400	3.33410300	-1.97064800
H	-2.79811000	3.00967900	1.46667400
H	-1.77217100	1.65216800	1.96172600
H	-1.09558500	3.29169800	1.83662700
H	-3.40653300	1.64477500	-0.72589900
H	-2.09368400	1.05944600	-1.75706900
H	-2.37350100	0.31653300	-0.16231600
H	0.72781900	4.38667200	0.61384000
H	1.08407300	3.34943400	2.01738800
H	2.39611900	4.08473500	1.09575400
H	1.07839200	3.38820100	-1.78927100
H	2.79468400	3.18688900	-1.42983100
H	1.83788800	1.78909000	-1.95080200
P	0.41832700	-1.20449900	-2.07414800
P	-0.18019300	-1.24911300	2.04761600
H	-0.85911000	-0.73442200	3.20188800
H	1.39714700	-2.23018300	-2.22667300
H	-0.81898200	-2.52119400	2.13182400
H	0.96814600	-1.65573900	2.79105500
H	-0.63770300	-1.97353600	-2.64662000
H	0.75682500	-0.57534500	-3.31919700

P: $Pd(PH_3)_2$ + $(CH_3)_3C–C(CH_3)_3$
E = -3774.94
H = -3582.32
G = -3626.79
***N*_{imag}** = 0

C	1.68114600	1.85126500	-0.31590000
C	2.96218200	1.03563800	-0.03586700
C	-1.60786800	1.75406500	0.33511000
C	1.61954500	2.97697900	0.72579900
C	1.72149300	2.42120300	-1.73940400
C	-1.60976500	2.89482700	-0.69181700
C	-2.84095100	0.87133900	0.04322600
C	-1.68007000	2.30221200	1.76596400

H	3.83177800	1.71817300	-0.01061000
H	2.93028000	0.52409000	0.93404000
H	3.17505500	0.28937100	-0.80977800
Pd	0.07775400	0.34072700	0.00096900
H	-2.54562300	3.47630500	-0.59343700
H	-0.78762800	3.60061000	-0.55710600
H	-1.57147200	2.51885700	-1.72125700
H	-2.58540300	2.92748400	1.88664600
H	-1.74224900	1.50226300	2.51351800
H	-0.82105300	2.93076800	2.01782200
H	-3.74772700	1.50415800	0.02657100
H	-2.78027500	0.37545400	-0.93346900
H	-3.01111000	0.10384800	0.80680300
H	0.75857400	3.63678100	0.60074500
H	1.60387600	2.58625500	1.75024200
H	2.52074500	3.61164100	0.63456400
H	0.82863300	3.00431500	-1.98277500
H	2.59042900	3.09730300	-1.85186500
H	1.82763300	1.63597500	-2.49760900
P	1.09685500	-1.34094700	-1.46503300
P	-0.84397700	-1.41700000	1.44156600
H	-1.55699700	-1.09509900	2.63257700
H	1.92400900	-2.48627200	-1.22123500
H	-1.60460300	-2.60353200	1.17669200
H	0.21281000	-2.10848100	2.09914100
H	0.08087600	-2.08032800	-2.13408100
H	1.78909800	-0.95834700	-2.65025200

RC: $PdCl^-$ + $H_3C–CH_3$

E = -1028.11

H = -977.67

G = -1003.89

N_{imag} = 0

C	-0.05716800	-1.53796200	0.00000000
C	-0.00071900	-0.00017500	0.00000000
Pd	1.97221700	1.34913800	0.00000000
H	-0.58189300	-1.92302800	0.88809600
H	0.95366200	-1.96121600	0.00000000
H	-0.58189300	-1.92302800	-0.88809600
H	-1.00137900	0.44863900	0.00000000
H	0.52518200	0.37647700	0.92642700
H	0.52518200	0.37647700	-0.92642700
Cl	3.92291100	2.66507600	0.00000000

TS: $PdCl^-$ + $H_3C–CH_3$

E = -999.94

H = -951.61

G = -976.55

N_{imag} = 1, -497.17 cm^{-1}

Cl	0.00000000	0.00000000	-2.35167900
Pd	0.00000000	0.00000000	0.05645600

C	0.93328200	0.07360300	2.04465500
C	-0.93328200	-0.07360300	2.04465500
H	1.66015400	0.37551800	1.24509900
H	-1.66015400	-0.37551800	1.24509900
H	1.25946500	-0.87987900	2.46796400
H	-1.25946500	0.87987900	2.46796400
H	0.97489300	0.85153800	2.81269400
H	-0.97489300	-0.85153800	2.81269400

P: PdCl$^-$ + H_3C–CH_3
E = -1024.93
H = -976.70
G = -1001.90
N_{imag} = 0

Cl	0.00000000	0.00000000	-4.35654800
Pd	0.00000000	0.00000000	-1.90361900
C	1.35759400	0.00581700	-0.36019200
C	-1.35759400	-0.00581700	-0.36019200
H	2.31996000	0.21976600	-0.84917400
H	-2.31996000	-0.21976600	-0.84917400
H	1.39668100	-0.98312800	0.11848700
H	-1.39668100	0.98312800	0.11848700
H	1.15361700	0.76945000	0.40291300
H	-1.15361700	-0.76945000	0.40291300

RC: PdCl$^-$ + CH_3H_2C–CH_3
E = -1390.88
H = -1322.36
G = -1350.84
N_{imag} = 0

C	1.51934300	0.02187500	-0.05128800
H	1.86406100	1.06402000	-0.05673100
C	-0.01825100	0.00826800	0.00001800
C	2.09469800	-0.71468000	-1.27870600
H	1.92617600	-0.43663800	0.86579900
H	-0.41574800	-1.01399900	0.04670500
H	-0.38996700	0.54833000	0.92201400
H	-0.44833000	0.48695100	-0.93056200
H	1.77182900	-1.76566900	-1.29634300
H	1.74816000	-0.24707100	-2.21012400
H	3.19458600	-0.70233000	-1.28141600
Pd	-1.48707500	1.88913300	-0.00837200
Cl	-2.92718300	3.75031000	-0.01649600

TS: PdCl$^-$ + CH_3H_2C–CH_3
E = -1361.21
H = -1294.83
G = -1322.41
N_{imag} = 1, -484.94 cm^{-1}

C	-0.95860200	-1.99642100	0.00498600
C	0.94461300	-1.97107500	0.01210400

H	-1.00352500	-2.72967500	0.81743100
C	-1.45032000	-2.60957500	-1.31215200
H	-1.65587300	-1.17121300	0.31288100
H	1.69404900	-1.20902300	-0.31729600
H	1.20533800	-2.31433900	1.01792800
H	1.02562500	-2.80576700	-0.69217100
H	-2.46963000	-3.01615500	-1.19865700
H	-1.47794600	-1.85842700	-2.10905200
H	-0.80531800	-3.43573400	-1.65194000
Pd	-0.01596500	-0.00045800	-0.00880900
Cl	0.02605500	2.40136200	-0.05455400

P: $PdCl^-$ + $CH_3H_2C\text{–}CH_3$

E = -1386.95
H = -1319.88
G = -1349.00
N_{imag} = 0

C	-1.14034200	1.73814800	0.33885600
C	1.35353400	1.58398500	-0.81528200
H	-0.68639200	2.70876100	0.58214700
C	-2.25747600	1.38512800	1.32991200
H	-1.52358000	1.78581300	-0.69056000
H	2.24013700	1.02396400	-1.15485400
H	0.78449400	1.91987900	-1.69472700
H	1.68352800	2.46647400	-0.24649500
H	-3.07053700	2.13778900	1.29355800
H	-2.69942800	0.40617700	1.10979200
H	-1.88643400	1.34725200	2.36136100
Pd	0.29928000	0.27378700	0.38569200
Cl	0.04481600	-1.83555700	1.60949900

RC: $PdCl^-$ + $(CH_3)_2HC\text{–}CH_3$

E = -1637.73
H = -1554.43
G = -1579.10
N_{imag} = 0

C	-0.64207500	2.19403600	1.32694500
C	0.84572500	2.57397700	1.43005100
C	-1.44067600	3.30429200	0.61257000
H	-0.80181800	1.28227400	0.66038700
C	-1.24194900	1.89321600	2.71251600
H	1.42244000	1.78279200	1.91974800
H	1.28302200	2.73559000	0.43875500
H	0.95989500	3.50593100	2.01397900
H	-2.49622500	3.02537600	0.50347700
H	-1.39232100	4.24397200	1.18657700
H	-1.03874500	3.49463500	-0.38981700
H	-2.28483000	1.56483500	2.62840200
H	-0.68370000	1.09684200	3.21537100
H	-1.21645500	2.79577300	3.34773900
Pd	-0.13542900	-0.35793900	0.23686200

TS: PdCl⁻ + $(CH_3)_2HC\text{–}CH_3$

E = -1723.17
H = -1638.96
G = -1668.32
N_{imag} = 1, -463.01 cm^{-1}

C	-1.03393100	1.66388800	1.16241900
C	0.90154500	1.71164400	0.97165200
C	-1.38036200	3.00237500	0.49919600
H	-1.71322600	0.89834500	0.69816200
C	-1.31029200	1.67478200	2.66984800
H	1.68787100	0.92655700	1.03975200
H	1.09593200	2.34629000	0.10246200
H	0.99102700	2.31361100	1.88361800
H	-2.43446300	3.26655600	0.69461100
H	-0.76105200	3.83091100	0.88207500
H	-1.24413300	2.95212200	-0.58740700
H	-2.36747700	1.92478300	2.86837100
H	-1.10689100	0.69482400	3.11455400
H	-0.69563300	2.42329500	3.19763200
Pd	-0.04622800	-0.14137900	0.29882100
Cl	0.11151400	-2.33690400	-0.65676500

P: PdCl⁻ + $(CH_3)_2HC\text{–}CH_3$

E = -1747.49
H = -1662.63
G = -1693.49
N_{imag} = 0

C	-2.09031500	-2.06908900	0.02301400
C	-0.05081500	0.01090700	0.11215100
C	-2.76501800	-1.63954900	-1.28977400
H	-2.18360400	-3.17008000	0.11655800
C	-2.77621900	-1.42651400	1.23792000
H	-0.88151000	0.46030200	-0.44435400
H	0.90962700	0.35007000	-0.29623600
H	-0.12490300	0.27584800	1.17564400
H	-3.85439700	-1.85468400	-1.27812200
H	-2.65740500	-0.55948900	-1.46486900
H	-2.33164900	-2.15634100	-2.15514600
H	-3.86756400	-1.62895500	1.24563100
H	-2.36001100	-1.80324100	2.18070800
H	-2.65602700	-0.33381400	1.23819200
Pd	-0.00096800	-2.03598200	-0.01405800
Cl	2.27477800	-2.93539000	-0.11086000

RC: PdCl⁻ + $(CH_3)_3C\text{–}CH_3$

E = -2116.08
H = -2012.58
G = -2043.01
N_{imag} = 0

C	1.53952000	-0.03775700	0.00000000

C	2.13526400	1.38990300	0.00000000
C	-0.01104400	0.02207000	0.00000000
C	2.02358600	-0.79660800	-1.26323900
C	2.02358600	-0.79660800	1.26323900
H	-0.44271000	-0.98670400	0.00000000
H	-0.39071700	0.54619700	0.92622600
H	-0.39071700	0.54619700	-0.92622600
H	3.23550100	1.35406200	0.00000000
H	1.80057600	1.95179100	-0.87989300
H	1.80057600	1.95179100	0.87989300
H	1.61472900	-1.81637300	1.29084500
H	3.12087400	-0.87159400	1.28875600
H	1.69700900	-0.27970600	2.17468000
H	1.61472900	-1.81637300	-1.29084500
H	1.69700900	-0.27970600	-2.17468000
H	3.12087400	-0.87159400	-1.28875600
Pd	-1.49701000	1.89847600	0.00000000
Cl	-2.98993700	3.71783400	0.00000000

TS: $PdCl^-$ + $(CH_3)_3C–CH_3$

E = -2075.40
H = -1973.48
G = -2004.86
N_{imag} = 1, -446.34 cm^{-1}

C	-1.42778000	-1.98257500	0.00000000
C	0.62137600	-2.06992100	0.00000000
C	-2.11129600	-1.46396300	-1.28115500
C	-1.56017800	-3.52373100	0.00000000
C	-2.11129600	-1.46396300	1.28115500
H	-3.13064200	-1.90177000	-1.36253600
H	-2.19846300	-0.37366600	-1.29485600
H	-1.55538600	-1.76580300	-2.17709900
H	0.70074100	-2.68556100	0.89948900
H	0.70074100	-2.68556100	-0.89948900
H	1.48896500	-1.34069200	0.00000000
H	-3.13064200	-1.90177000	1.36253600
H	-1.55538600	-1.76580300	2.17709900
H	-2.19846300	-0.37366600	1.29485600
H	-2.63456000	-3.78425700	0.00000000
H	-1.10990300	-3.98419100	-0.88799000
H	-1.10990300	-3.98419100	0.88799000
Pd	0.04705700	-0.00432800	0.00000000
Cl	-0.10928100	2.40631000	0.00000000

P: $PdCl^-$ + $(CH_3)_3C–CH_3$

E = -2110.37
H = -2007.78
G = -2040.40
N_{imag} = 0

Pd	-2.04319700	-0.00045500	-0.01998000
C	-2.11964000	2.10829700	-0.01283800

C	0.00056100	0.03171800	0.16519200
C	-1.59952800	2.71649100	-1.32690300
C	-3.64493400	2.31669700	0.08430100
C	-1.42970900	2.78155200	1.18427200
H	0.47727200	0.87459700	-0.34865600
H	0.34359300	-0.91865400	-0.26250400
H	0.23104200	0.06769300	1.23870200
H	-1.78399000	3.81300600	-1.35929800
H	-0.51906900	2.56654900	-1.44561300
H	-2.09283800	2.27250000	-2.20105600
H	-4.17980600	1.82153000	-0.73883000
H	-4.05009700	1.93163100	1.02956500
H	-3.90292400	3.39660100	0.03167600
H	-1.60910700	3.87867500	1.18286100
H	-1.80318900	2.38684600	2.13805200
H	-0.34306000	2.63180000	1.16539600
Cl	-2.86667900	-2.30157400	-0.11143900

RC: $PdCl^-$ + $CH_3H_2C{-}CH_2CH_3$

E = -1752.88
H = -1666.87
G = -1696.17
N_{imag} = 0

C	-1.53797500	-0.06618600	-0.00012300
C	0.00127000	0.03929700	0.00019600
C	0.69465000	-1.33435400	0.00022800
C	-2.26927400	1.28830900	0.00021100
H	0.32802300	0.61192700	0.91869900
H	0.32832100	0.61195100	-0.91827300
H	1.78343600	-1.21758500	0.00067200
H	0.41431300	-1.92336900	0.88856400
H	0.41497400	-1.92311900	-0.88845900
H	-1.85564700	-0.65124000	-0.88111800
H	-1.85596500	-0.65185300	0.88032200
H	-3.36074800	1.14813100	-0.00029300
H	-1.99267000	1.88183000	0.88008200
H	-1.99189700	1.88269100	-0.87882100
Pd	1.30465200	2.07845700	-0.00007700
Cl	2.61783400	4.03131600	0.00019000

TS: $PdCl^-$ + $CH_3H_2C{-}CH_2CH_3$

E = -1722.27
H = -1637.75
G = -1667.61
N_{imag} = 1, -461.17 cm^{-1}

C	-0.96630400	-2.02530600	0.01621100
C	0.96615700	-2.02546700	-0.01612000
C	-1.45390300	-2.57541400	1.36309100
C	1.45363100	-2.57556700	-1.36306600
H	-1.66388700	-1.21995100	-0.33052100
H	-1.01297500	-2.79699300	-0.76012600

H	1.01270500	-2.79723800	0.76014300
H	1.66391900	-1.22029200	0.33064200
H	-2.45784000	-3.02242100	1.26299700
H	-0.78828300	-3.35910100	1.75852900
H	-1.51590300	-1.78066200	2.11421600
H	2.45765600	-3.02240200	-1.26310200
H	0.78806600	-3.35937400	-1.75835400
H	1.51538300	-1.78083600	-2.11423100
Pd	0.00009000	-0.03902700	0.00015800
Cl	0.00038900	2.36655100	0.00023200

P: $PdCl^-$ + CH_3H_2C–CH_2CH_3

E = -1748.66
H = -1663.97
G = -1694.30
N_{imag} = 0

C	-0.22657100	0.86353100	-0.82673700
C	1.42233600	2.38299800	0.76553300
C	-0.71379000	-0.39187000	-1.56517300
C	2.64048200	2.69757100	1.64644100
H	-0.98973200	1.22536200	-0.12158700
H	-0.00891100	1.67475700	-1.53705000
H	0.48516900	2.56924400	1.31119000
H	1.42017600	3.01083900	-0.13774000
H	-1.64424800	-0.19534800	-2.13465500
H	0.03580200	-0.75642500	-2.27972300
H	-0.92405600	-1.21482600	-0.86964600
H	2.65080100	3.75921700	1.96495000
H	2.64982700	2.08314700	2.55622600
H	3.58264200	2.50590700	1.11663700
Pd	1.49110600	0.39182600	0.21975400
Cl	2.91756700	-1.55783200	0.61468100

RC: $PdCl^-$ + $(CH_3)_2HC$–$CH(CH_3)_2$

E = -2471.93
H = -2350.03
G = -2383.35
N_{imag} = 0

C	0.54955700	2.50888700	0.95605500
H	1.27341600	3.67578800	2.65908400
C	-0.93900100	2.92586100	1.08386900
C	1.31429800	2.63856400	2.28630800
C	1.28727800	3.28846600	-0.14811200
H	1.24293600	4.37300100	0.04555400
C	-1.69999100	2.77112100	-0.24883900
C	-1.67281800	2.12438800	2.17858600
H	0.90516300	1.97697800	3.05400300
H	2.36848000	2.36833400	2.14665100
H	-0.94321300	3.99561800	1.36888900
Pd	-0.12485100	-0.19129000	0.24998300
H	2.34272200	2.99181300	-0.18243500

H	0.85996100	3.09416700	-1.13492300
Cl	-0.38985200	-2.46038800	-0.34095900
H	-2.74036800	2.38412400	2.19650900
H	-1.57712900	1.04513400	1.95316900
H	-1.26393100	2.30940900	3.17878600
H	-2.76568900	3.00078200	-0.11315600
H	-1.31033300	3.43031400	-1.03319400
H	-1.60712500	1.72332500	-0.59144600
H	0.63555100	1.40893500	0.66118200

TS: $PdCl^-$ + $(CH_3)_2HC{-}CH(CH_3)_2$

E = -2437.91
H = -2317.45
G = -2350.60
N_{imag} = 1, -403.19 cm^{-1}

C	1.70337100	-1.84049900	0.67759100
H	1.58761800	-2.13971400	-1.44671400
H	4.43264500	-0.68573600	-2.80881500
Cl	3.28087500	-5.27780100	-2.90878800
C	4.23824600	-1.44519700	-0.77120000
H	4.30863500	0.60174200	-1.58711800
Pd	3.24157000	-3.18970400	-1.72671100
H	0.74957900	0.09325800	-0.98827900
H	4.86218600	-2.36668000	-0.93647900
C	2.18475100	-1.50135900	-0.73767800
C	1.83444300	-0.06435200	-1.12030800
C	4.70853300	-0.40036700	-1.78929400
H	2.34319900	0.68522300	-0.49558200
H	2.07075300	0.14689100	-2.16788700
H	0.60051300	-1.80313100	0.70831500
H	2.00804200	-2.84973800	0.97022400
H	2.06841000	-1.13286100	1.43319300
H	4.01361100	-0.10490400	0.96591000
H	4.32769000	-1.80429600	1.38300600
H	5.63091500	-0.78841700	0.74313900
H	5.80900000	-0.32102400	-1.75127900
C	4.55320600	-1.01395800	0.66022200

P: $PdCl^-$ + $(CH_3)_2HC{-}CH(CH_3)_2$

E = -2468.90
H = -2347.68
G = -2381.79
N_{imag} = 0

C	-1.54359200	-0.94537000	0.02540300
C	1.56101700	-1.52225700	-0.20625700
H	-2.18472100	-0.09537100	-0.24143300
C	-1.70940600	-2.05296700	-1.00737800
C	-1.87280100	-1.40951200	1.44393400
C	1.46302700	-2.67823100	0.79255100
C	1.72985800	-2.04295000	-1.63955300
H	2.46683200	-0.92923700	0.05037700

```
H     1.89074800  -1.22295200  -2.35016400
Pd    0.25237300   0.08157400   0.01786900
Cl   -0.65291200   2.34427700   0.33872200
H    -2.89753200  -1.83345200   1.48399500
H    -1.18579900  -2.19350600   1.78630800
H    -1.82318100  -0.57655000   2.15253000
H    -2.75270300  -2.42746400  -1.00349200
H    -1.48430300  -1.69917800  -2.01956500
H    -1.06078800  -2.91236300  -0.79796900
H     2.32407100  -3.37372700   0.70743900
H     1.42429100  -2.31591300   1.82693600
H     0.55829200  -3.27831100   0.62148800
H     2.59101600  -2.73945500  -1.72517100
H     0.84228500  -2.59623000  -1.97267300
```

RC: $PdCl^-$ + $(CH_3)_3C\text{–}C(CH_3)_3$

E = -3193.86
H = -3035.70
G = -3072.79
N_{imag} = 0

```
C    0.63372100   2.66963000   1.08220500
C    1.12141600   1.21188400   0.81457000
C   -0.96818400   2.80958900   1.07646000
C    1.22725700   3.10177800   2.45572900
C    1.28423700   3.57663000  -0.00254700
C   -1.41048100   4.23822600   1.50670000
C   -1.55607300   2.53732600  -0.33575800
C   -1.62732200   1.79660300   2.05276700
H    2.21768100   1.19414500   0.83885700
H    0.76723100   0.49218100   1.57896800
H    0.83547600   0.87372800  -0.23873900
Pd   0.08608000  -0.82327800  -0.01791900
H   -2.50220200   4.32003000   1.42074600
H   -1.14882500   4.45924500   2.54857600
H   -0.97505900   5.02080900   0.87261700
H   -2.71360200   1.96175200   2.07219200
H   -1.45408300   0.76170900   1.72894700
H   -1.26054600   1.91007500   3.08089500
H   -2.65281700   2.58527800  -0.28924700
H   -1.22666900   3.28175600  -1.07141600
H   -1.28509600   1.53541100  -0.69522700
H    1.05883900   4.16531800   2.66655200
H    0.81122600   2.51838100   3.28467300
H    2.31268100   2.93624900   2.45335000
H    1.01586900   4.63415300   0.11689400
H    2.37791100   3.50617000   0.07002300
H    1.00532700   3.26329300  -1.01374000
Cl  -0.86146800  -2.86291900  -0.69897000
```

TS: $PdCl^-$ + $(CH_3)_3C\text{–}C(CH_3)_3$

E = -3134.72

H = -2979.39
G = -3017.90
$\boldsymbol{N_{imag}}$ = 1, -227.18 cm^{-1}

C	1.22962100	2.10690100	0.98111800
C	1.98729700	0.91999800	1.59239600
C	-1.19658200	2.05032200	1.00516400
C	1.41935200	3.29084600	1.93390800
C	1.70186900	2.50097300	-0.41800900
C	-1.43517100	3.45098700	1.57608300
C	-1.91830800	1.99144100	-0.34824300
C	-1.66861200	1.00586400	2.01637800
H	3.04062000	1.22509000	1.79609000
H	1.55352400	0.62029200	2.55458300
H	2.00720200	0.03222500	0.94103800
Pd	0.06522400	0.04000200	-0.18343300
H	-2.53226400	3.59111600	1.64417800
H	-1.03398600	3.59574700	2.58307800
H	-1.05720600	4.25001700	0.92723600
H	-2.71600000	1.22403100	2.32304600
H	-1.62948100	-0.01213700	1.60725400
H	-1.05768500	1.02688500	2.92856000
H	-2.97936000	2.30609000	-0.20997900
H	-1.47388700	2.68759400	-1.07049500
H	-1.91038500	0.98851300	-0.80006200
H	0.99539500	4.22985900	1.56731100
H	1.03164200	3.09817200	2.94139400
H	2.51059700	3.45096200	2.04036600
H	1.07315900	3.29439400	-0.84354300
H	2.73896700	2.90079300	-0.36788200
H	1.68962500	1.65061600	-1.11245400
Cl	0.05663100	-2.02099300	-1.40458100

P: $PdCl^-$ + $(CH_3)_3C-C(CH_3)_3$
E = -3192.24
H = -3036.22
G = -3072.38
$\boldsymbol{N_{imag}}$ = 0

C	-1.68229400	-1.04546400	-0.01900000
C	1.55634400	-1.40357700	-0.19280900
C	-2.74377600	-0.02243600	-0.47465100
C	-1.71777900	-2.25114700	-0.96644100
C	-1.97301900	-1.49265900	1.42420200
C	1.49828800	-2.58469900	0.78543100
C	1.69952700	-1.91242800	-1.63685600
C	2.77152500	-0.51435800	0.15591000
H	-3.75113200	-0.48876100	-0.43567900
H	-2.75207600	0.86900900	0.15927400
H	-2.57046200	0.30741800	-1.50591900
H	-2.97024800	-1.98058200	1.47959300
H	-1.23470500	-2.21626000	1.78790100
H	-1.98297300	-0.64105900	2.11396800

H	-2.72728700	-2.71195200	-0.95159700
H	-1.50757000	-1.95839600	-2.00218300
H	-1.00567400	-3.03275600	-0.68224600
H	2.41272600	-3.21099300	0.70342400
H	1.42589400	-2.24370500	1.82590800
H	0.64477700	-3.24482600	0.59076600
H	2.61425100	-2.53561700	-1.74597600
H	0.84999200	-2.53202900	-1.94358000
H	1.78118700	-1.08218600	-2.34964200
H	3.72104200	-1.06830200	-0.00306700
H	2.81929000	0.38434700	-0.47548400
H	2.75387000	-0.18781300	1.20337900
Pd	0.09092800	0.11235900	0.02306500
Cl	-0.32424500	2.53437300	0.39620700

Table S5. Cartesian coordinates (in Å), energies (*E*, *H* and *G*, in kcal mol^{-1}), and number of imaginary vibrational frequencies (N_{imag}) of all stationary points of the C–H activation reactions in the gas phase, computed at ZORA-BLYP/TZ2P.

R: $Pd(PH_3)_2$
E = -756.21
H = -718.97
G = -743.55
N_{imag} = 0

Pd	0.00000000	0.00000000	0.00000000
P	0.00000000	0.00000000	2.28490600
H	0.60928100	1.05530600	3.01943100
H	0.60928100	-1.05530600	3.01943100
H	-1.21856200	0.00000000	3.01943100
P	0.00000000	0.00000000	-2.28490600
H	-1.21856200	0.00000000	-3.01943100
H	0.60928100	-1.05530600	-3.01943100
H	0.60928100	1.05530600	-3.01943100

R: $PdCl^-$
E = -119.61
H = -116.86
G = -134.51
N_{imag} = 0

Pd	0.00000000	0.00000000	-0.00847700
Cl	0.00000000	0.00000000	2.32586400

R: H_3C–H
E = -535.00
H = -505.28
G = -518.55
N_{imag} = 0

C	0.00000000	0.00000000	0.00000000
H	0.63179000	0.63179000	-0.63179000
H	-0.63179000	0.63179000	0.63179000
H	0.63179000	-0.63179000	0.63179000
H	-0.63179000	-0.63179000	-0.63179000

R: CH_3H_2C–H
E = -896.24
H = -847.97
G = -864.88
N_{imag} = 0

C	0.00000000	0.00000000	0.76969800
H	0.00000000	1.02232700	1.16809400
H	0.88536100	-0.51116400	1.16809400
H	-0.88536100	-0.51116400	1.16809400
C	0.00000000	0.00000000	-0.76969800
H	-0.88536100	0.51116400	-1.16809400
H	0.88536100	0.51116400	-1.16809400
H	0.00000000	-1.02232700	-1.16809400

R: $(CH_3)_2HC-H$

E = -1258.70
H = -1192.28
G = -1211.46
N_{imag} = 0

C	0.00000000	0.00000000	0.02366900
C	1.28532900	0.00000000	0.87228000
C	-1.28532900	0.00000000	0.87228000
H	0.00000000	0.87917500	-0.63671600
H	0.00000000	-0.87917500	-0.63671600
H	2.18285900	0.00000000	0.24121600
H	1.33141900	0.88607700	1.51970000
H	1.33141900	-0.88607700	1.51970000
H	-2.18285900	0.00000000	0.24121600
H	-1.33141900	-0.88607700	1.51970000
H	-1.33141900	0.88607700	1.51970000

R: $(CH_3)_3C-H$

E = -1621.51
H = -1537.28
G = -1558.89
N_{imag} = 0

C	0.00000000	0.00000000	0.35708700
C	1.47039600	0.00000000	-0.11180500
C	-0.73519800	-1.27340000	-0.11180500
C	-0.73519800	1.27340000	-0.11180500
H	0.00000000	0.00000000	1.45870500
H	2.00490500	0.88726100	0.25132600
H	2.00490500	-0.88726100	0.25132600
H	1.52985200	0.00000000	-1.20956700
H	-1.77084300	1.29266800	0.25132600
H	-0.23406200	2.17992900	0.25132600
H	-0.76492600	1.32489100	-1.20956700
H	-1.77084300	-1.29266800	0.25132600
H	-0.76492600	-1.32489100	-1.20956700
H	-0.23406200	-2.17992900	0.25132600

RC: Pd + H_3C-H

E = -541.63
H = -511.16
G = -531.35
N_{imag} = 0

C	0.09092400	0.12323400	0.00000000
H	0.87958500	-0.63213200	0.00000000
H	0.19004900	0.72110500	0.94492000
H	-0.89964300	-0.33646800	0.00000000
H	0.19004900	0.72110500	-0.94492000
Pd	0.48639900	2.48182200	0.00000000

TS: Pd + H_3C-H

E = -530.86
H = -503.69
G = -522.47
N_{imag} = 1, -775.25 cm^{-1}

C	-0.13651000	0.16934700	-0.13697000
H	0.89350900	-0.09957500	-0.37965900
H	-0.16311200	1.15798300	0.34594900
H	-0.59123400	-0.57701300	0.51753900
H	-0.72988000	-0.51699700	-1.47752600
Pd	-1.32476000	0.90412400	-1.72015400

P: Pd + H_3C–H
E = -537.98
H = -509.51
G = -529.55
N_{imag} = 0

C	0.00018700	-0.00014000	0.00000000
H	0.37175800	-0.48986000	-0.90373700
H	0.23756500	1.07444300	0.00000000
H	0.37175800	-0.48986000	0.90373700
H	-1.94534800	-1.53603300	0.00000000
Pd	-2.02058900	-0.00026000	0.00000000

RC: Pd + CH_3H_2C–H
E = -902.91
H = -853.86
G = -876.73
N_{imag} = 0

C	0.21141800	-2.77562100	0.00000000
C	0.87834200	-1.38802600	0.00000000
H	0.50508700	-3.35077200	-0.88699400
H	-0.88045300	-2.69131200	0.00000000
H	0.50508700	-3.35077200	0.88699400
H	1.97160300	-1.44694700	0.00000000
H	0.60143600	-0.82809900	-0.93567800
H	0.60143600	-0.82809900	0.93567800
Pd	-0.21920300	0.76691900	0.00000000

TS: Pd + CH_3H_2C–H
E = -891.60
H = -845.46
G = -867.18
N_{imag} = 1, -785.42 cm^{-1}

C	1.30496600	-0.64963400	-0.22874100
C	2.43449400	0.35104200	0.03892200
H	2.20224800	1.34601700	-0.35392000
H	2.64323200	0.45531000	1.10920000
H	3.35932000	0.00606000	-0.44984500
H	1.08209600	-0.75192000	-1.30306900
H	1.54453500	-1.64908900	0.14628300
H	0.31045200	-0.52922400	1.06239000

Pd -0.69910600 0.05084000 0.01893100

P: Pd + CH_3H_2C–H

E = -899.97
H = -853.20
G = -875.26
N_{imag} = 0

C	1.22590200	-0.62704200	-0.33034600
C	2.33420800	0.34561900	0.05722700
H	2.19636400	1.33661400	-0.38800900
H	2.40518500	0.46684900	1.14267400
H	3.30457100	-0.04221500	-0.29735300
H	1.14675500	-0.75997400	-1.42455300
H	1.33926400	-1.60617800	0.14659100
H	-0.42267800	-0.03917100	1.54412200
Pd	-0.68189200	-0.02657800	0.02364300

RC: Pd + $(CH_3)_2HC$–H

E = -1265.19
H = -1197.93
G = -1222.42
N_{imag} = 0

C	1.29063500	0.00000000	-0.90846100
C	0.00000000	0.00000000	-0.06711900
C	-1.29063500	0.00000000	-0.90846100
H	2.18277600	0.00000000	-0.27337500
H	1.33307000	-0.88739500	-1.55442800
H	1.33307000	0.88739500	-1.55442800
H	0.00000000	-0.92763600	0.57243100
H	0.00000000	0.92763600	0.57243100
H	-2.18277600	0.00000000	-0.27337500
H	-1.33307000	0.88739500	-1.55442800
H	-1.33307000	-0.88739500	-1.55442800
Pd	0.00000000	0.00000000	2.37570100

TS: Pd + $(CH_3)_2HC$–H

E = -1253.16
H = -1189.03
G = -1212.72
N_{imag} = 1, -768.78 cm^{-1}

C	-0.75274800	-0.42504800	1.28462300
C	-0.03033400	0.00038900	0.00000000
C	-0.75274800	-0.42504800	-1.28462300
H	-0.21470000	-0.09982200	2.18050800
H	-1.75693100	0.02937400	1.30859400
H	-0.87977700	-1.51266000	1.34297800
H	1.11873500	-1.19287300	0.00000000
H	-0.21470000	-0.09982200	-2.18050800
H	-0.87977700	-1.51266000	-1.34297800
H	-1.75693100	0.02937400	-1.30859400
H	0.09149500	1.09728900	0.00000000

Pd 2.13098100 -0.00440200 0.00000000

P: Pd + $(CH_3)_2HC-H$

E = -1262.86
H = -1198.24
G = -1222.50
N_{imag} = 0

C	-0.60681900	-0.40678800	1.27977000
C	0.03623100	0.12420100	0.00000000
C	-0.60681900	-0.40678800	-1.27977000
H	-0.12629900	-0.01402300	2.18160800
H	-1.66972600	-0.10771400	1.30816300
H	-0.57165500	-1.50114600	1.31993700
H	2.09590900	-1.56656600	0.00000000
H	-0.12629900	-0.01402300	-2.18160800
H	-0.57165500	-1.50114600	-1.31993700
H	-1.66972600	-0.10771400	-1.30816300
H	0.03821700	1.23415600	0.00000000
Pd	2.07153100	-0.01515600	0.00000000

RC: Pd + $(CH_3)_3C-H$

E = -1626.92
H = -1541.90
G = -1569.01
N_{imag} = 0

C	-0.01103100	-0.03892400	0.00000000
C	1.52768100	0.01362900	0.00000000
C	-0.55668800	-0.70972600	1.27388700
C	-0.55668800	-0.70972600	-1.27388700
H	-0.38531800	1.04506900	0.00000000
H	1.91031800	0.53120900	-0.88765300
H	1.91031800	0.53120900	0.88765300
H	1.94098800	-1.00601100	0.00000000
H	-1.65298500	-0.70443500	-1.29066200
H	-0.19965300	-0.20192700	-2.17770500
H	-0.22383100	-1.75728000	-1.32202400
H	-1.65298500	-0.70443500	1.29066200
H	-0.22383100	-1.75728000	1.32202400
H	-0.19965300	-0.20192700	2.17770500
Pd	-1.02207000	2.80695700	0.00000000

TS: Pd + $(CH_3)_3C-H$

E = -1612.04
H = -1530.01
G = -1555.90
N_{imag} = 1, -774.67 cm^{-1}

C	0.05355400	0.07096900	0.00000000
C	1.23036200	-0.93405100	0.00000000
C	0.11245500	0.93768300	-1.27176300
C	0.11245500	0.93768300	1.27176300
H	-1.52653900	-0.01737100	0.00000000

H	1.24509900	-1.57135000	0.89099300
H	1.24509900	-1.57135000	-0.89099300
H	2.17268800	-0.35253500	0.00000000
H	-0.68355800	1.69055900	1.29305400
H	-0.68355800	1.69055900	-1.29305400
H	1.08049000	1.46667300	1.30633300
H	1.08049000	1.46667300	-1.30633300
H	0.02794000	0.32943600	-2.17847100
H	0.02794000	0.32943600	2.17847100
Pd	-1.47381900	-1.57669500	0.00000000

P: Pd + $(CH_3)_3C$–H

E = -1626.62
H = -1544.28
G = -1570.34
N_{imag} = 0

C	-0.01116900	-0.01218000	0.00000000
C	0.64080300	-0.55295800	-1.27021800
C	0.64080300	-0.55295800	1.27021800
C	-1.54906300	-0.04475700	0.00000000
H	1.33196900	2.52936400	0.00000000
H	0.19455800	-0.13251500	-2.17725600
H	1.71446800	-0.33941900	-1.28350600
H	0.51714000	-1.65085400	-1.30933600
H	-1.99659200	0.99057600	0.00000000
H	-1.95950800	-0.52016500	-0.89805600
H	-1.95950800	-0.52016500	0.89805600
H	0.19455800	-0.13251500	2.17725600
H	0.51714000	-1.65085400	1.30933600
H	1.71446800	-0.33941900	1.28350600
Pd	-0.16346900	2.02137000	0.00000000

RC: $Pd(PH_3)_2$ + H_3C–H

E = -1291.20
H = -1225.42
G = -1254.08
N_{imag} = 0

Pd	0.00114500	-0.00731900	0.00000000
P	2.28609600	0.01246400	0.00000000
H	3.00911600	1.23780000	0.00000000
H	3.02596100	-0.59011300	-1.05539700
H	3.02596100	-0.59011300	1.05539700
P	-2.28403500	-0.00773500	0.00000000
H	-3.01858300	-0.61675000	1.05540100
H	-3.01858300	-0.61675000	-1.05540100
H	-3.01770000	1.21125400	0.00000000
H	-0.00545500	-3.83695400	0.00000000
C	-0.04881800	-4.93025400	0.00000000
H	0.96700600	-5.33744600	0.00000000
H	-0.57831500	-5.27566800	0.89330700
H	-0.57831500	-5.27566800	-0.89330700

TS: $Pd(PH_3)_2$ + H_3C-H
E = -1258.53
H = -1193.99
G = -1223.63
N_{imag} = 1, -717.00 cm^{-1}

C	-0.01486200	-0.00101700	0.00000000
Pd	2.18806500	-0.01430800	0.00000000
H	-0.66092400	-0.88035900	0.00000000
H	-0.21993700	0.58320500	-0.90245300
H	-0.21993700	0.58320500	0.90245300
H	1.17958100	-1.24373400	0.00000000
H	3.51468700	2.91740000	-1.05028200
H	5.53288500	-0.62723500	0.00000000
H	4.47298800	-2.10362400	-1.05246500
H	3.51468700	2.91740000	1.05028200
H	1.73962800	3.32694000	0.00000000
H	4.47298800	-2.10362400	1.05246500
P	2.75463300	2.32497000	0.00000000
P	4.21633700	-1.18070000	0.00000000

P: $Pd(PH_3)_2$ + H_3C-H
E = -1263.56
H = -1198.01
G = -1227.28
N_{imag} = 0

C	-0.01886200	-0.00731400	0.00000000
Pd	2.10698300	-0.02139600	0.00000000
H	-0.46087900	-1.00335200	0.00000000
H	-0.34144400	0.53578500	-0.89686700
H	-0.34144400	0.53578500	0.89686700
H	1.83005600	-1.59001300	0.00000000
H	2.81325600	3.08649700	-1.05521800
H	5.50871600	0.24573000	0.00000000
H	4.83257200	-1.45328600	-1.05807900
H	2.81325600	3.08649700	1.05521800
H	0.97500500	3.10281600	0.00000000
H	4.83257200	-1.45328600	1.05807900
P	2.18916100	2.36253200	0.00000000
P	4.37565400	-0.62286900	0.00000000

RC: $Pd(PH_3)_2$ + CH_3H_2C-H
E = -1652.44
H = -1567.48
G = -1600.22
N_{imag} = 0

C	1.72674500	4.72150000	1.74987800
C	0.93020300	4.47440300	0.45674300
H	1.87551100	5.79386600	1.92827000
H	1.20646700	4.30908900	2.62344800
H	2.71775100	4.25280200	1.70305600

H	1.45032700	4.88736500	-0.41666100
H	-0.06113400	4.94251600	0.50372100
H	0.78267300	3.40216500	0.27829900
Pd	0.09650400	-2.27713800	-0.35607700
P	2.29682400	-2.88783600	-0.42690600
H	2.92712200	-3.49593200	0.69456400
H	3.31959300	-1.92663600	-0.66149800
H	2.76567100	-3.83144600	-1.38322000
P	-2.10521500	-1.67408900	-0.27878600
H	-2.88379000	-1.89481300	0.89164900
H	-3.05105900	-2.22922400	-1.18563900
H	-2.50733700	-0.32169600	-0.46402800

TS: $Pd(PH_3)_2$ + $CH_3H_2C{-}H$

E = -1618.17
H = -1534.63
G = -1568.60
N_{imag} = 1, -717.91 cm^{-1}

Pd	-0.05729500	0.01832400	-0.03851700
H	2.03727600	2.65969500	0.23071500
H	1.86261300	-2.78575800	-0.30960200
H	0.02901800	-3.18487600	0.63063400
H	3.02496700	0.96620600	0.98886000
H	2.88659100	1.26070600	-1.08601700
H	0.10884300	-2.92760100	-1.45576600
P	2.00418300	1.24640900	0.03336200
P	0.54515300	-2.23234900	-0.29261200
C	-1.64697400	1.57864600	0.16122200
C	-1.85919200	1.98624700	1.62620200
H	-1.64403800	-0.13887200	-0.01906900
H	-2.48909600	2.88826600	1.69002600
H	-0.91494500	2.20813400	2.13703800
H	-2.36051300	1.19661900	2.19810900
H	-1.10625200	2.34469600	-0.40598100
H	-2.60955200	1.44826400	-0.34032200

P: $Pd(PH_3)_2$ + $CH_3H_2C{-}H$

E = -1622.95
H = -1539.17
G = -1570.92
N_{imag} = 0

C	2.15746100	0.00023700	0.00000000
Pd	0.01694000	0.01667600	0.00000000
C	2.95609300	1.31122400	0.00000000
H	2.41090700	-0.59948000	-0.88652900
H	2.41090700	-0.59948000	0.88652900
H	0.23163500	1.59628000	0.00000000
H	-0.64877600	-3.09538800	-1.05544500
H	-3.40074000	-0.31065800	0.00000000
H	-2.73848300	1.39354900	-1.05772100
H	-0.64877600	-3.09538800	1.05544500

H	1.19092600	-3.09037100	0.00000000
H	-2.73848300	1.39354900	1.05772100
P	-0.03310100	-2.36529700	0.00000000
P	-2.27331200	0.56722500	0.00000000
H	4.04085600	1.10884100	0.00000000
H	2.73488600	1.92117100	0.88323800
H	2.73488600	1.92117100	-0.88323800

RC: Pd(PH$_3$)$_2$ + (CH$_3$)$_2$HC–H

E = -2014.87

H = -1911.74

G = -1945.77

N_{imag} = 0

C	-4.34222000	-1.25162700	1.31911700
C	-4.01482400	-0.40502600	0.07555000
C	-4.48692700	-1.05353300	-1.23854500
H	-3.99264500	-0.76575900	2.23856100
H	-5.42448300	-1.41220000	1.41586500
H	-3.86510000	-2.23924900	1.26152100
H	-2.93035400	-0.23227000	0.02768600
H	-4.23888400	-0.42917000	-2.10593100
H	-4.01455400	-2.03385100	-1.38741400
H	-5.57454200	-1.20708400	-1.23667600
H	-4.47922700	0.58651200	0.17877600
Pd	1.22641100	0.53442500	-0.04860600
H	1.79769100	3.73086500	-0.24706700
H	3.06770600	-2.14608800	-0.05288600
H	0.09150100	3.41700900	0.95582900
H	-0.06602100	3.31328300	-1.14647300
H	1.21348200	-2.59460500	-0.95635600
H	1.36544200	-2.49313400	1.14630800
P	0.75721800	2.76843500	-0.12167800
P	1.72040400	-1.69533600	0.02293000

TS: Pd(PH$_3$)$_2$ + (CH$_3$)$_2$HC–H

E = -1979.22

H = -1878.97

G = -1911.44

N_{imag} = 1, -670.46 cm^{-1}

C	-2.97662900	-0.50778700	1.28061600
C	-2.29262500	-0.00931900	0.00000000
C	-2.97662900	-0.50778700	-1.28061600
H	-2.47451500	-0.13767200	2.18203500
H	-4.02317800	-0.15983600	1.31543300
H	-2.99008300	-1.60386400	1.33691400
H	-1.04244800	-1.23064800	0.00000000
H	-2.47451500	-0.13767200	-2.18203500
H	-2.99008300	-1.60386400	-1.33691400
H	-4.02317800	-0.15983600	-1.31543300
H	-2.32347200	1.08621100	0.00000000
Pd	-0.02333600	-0.00658100	0.00000000

H	1.38014100	2.87105500	-1.05052400
H	3.32935200	-0.68361800	0.00000000
H	1.38014100	2.87105500	1.05052400
H	-0.37741000	3.34944700	0.00000000
H	2.23241500	-2.13206100	-1.05195200
H	2.23241500	-2.13206100	1.05195200
P	0.59856100	2.30711200	0.00000000
P	1.99745800	-1.20247400	0.00000000

P: $Pd(PH_3)_2$ + $(CH_3)_2HC-H$

E = -1984.47
H = -1882.69
G = -1916.08
N_{imag} = 0

C	-2.82423100	-0.21163000	1.27536800
C	-2.15408900	0.32051000	0.00000000
C	-2.82423100	-0.21163000	-1.27536800
H	-2.34586700	0.16861800	2.18644600
H	-3.88534500	0.09653900	1.30859500
H	-2.79511500	-1.30661000	1.31957500
H	-0.53919900	-1.53095600	0.00000000
H	-2.34586700	0.16861800	-2.18644600
H	-2.79511500	-1.30661000	-1.31957500
H	-3.88534500	0.09653900	-1.30859500
H	-2.24506100	1.41620100	0.00000000
Pd	-0.01782100	-0.01458100	0.00000000
H	1.27936000	2.87322500	-1.05500400
H	3.41939500	-0.36609800	0.00000000
H	1.27936000	2.87322500	1.05500400
H	-0.51189200	3.28382700	0.00000000
H	2.42130600	-1.89770700	-1.05764200
H	2.42130600	-1.89770700	1.05764200
P	0.51542400	2.29847600	0.00000000
P	2.13485100	-0.99380200	0.00000000

RC: $Pd(PH_3)_2$ + $(CH_3)_3C-H$

E = -2377.71
H = -2257.93
G = -2290.36
N_{imag} = 0

C	-2.30470900	-5.94389600	1.27314500
C	-2.98657100	-5.40097700	0.00000000
C	-2.30470900	-5.94389600	-1.27314500
H	-2.76500200	-5.53018700	2.17955200
H	-2.38898500	-7.03869400	1.32526500
H	-1.23710000	-5.69007800	1.29274800
H	-2.87072600	-4.30569800	0.00000000
H	-2.76500200	-5.53018700	-2.17955200
H	-1.23710000	-5.69007800	-1.29274800
H	-2.38898500	-7.03869400	-1.32526500
Pd	-1.98605800	1.25393000	0.00000000

H	-4.99654500	1.90161000	-1.05543300
H	1.02139300	0.00544500	0.00000000
H	-4.99654500	1.90161000	1.05543300
H	-5.02205400	0.07383900	0.00000000
H	1.03843900	1.83338400	-1.05547500
H	1.03843900	1.83338400	1.05547500
P	-4.27047400	1.28219400	0.00000000
P	0.29822700	1.23104000	0.00000000
C	-4.49772600	-5.71269200	0.00000000
H	-4.99116000	-5.29521900	-0.88711200
H	-4.99116000	-5.29521900	0.88711200
H	-4.67316200	-6.79788300	0.00000000

TS: $Pd(PH_3)_2$ + $(CH_3)_3C–H$

E = -2337.57
H = -2218.94
G = -2254.05
N_{imag} = 1, -623.94 cm^{-1}

C	-0.68145800	-0.64707700	1.26894200
C	-0.08148800	-0.01281000	0.00000000
C	-0.68145800	-0.64707700	-1.26894200
H	-0.22175300	-0.24412300	2.17923400
H	-1.76383000	-0.43113200	1.32022800
H	-0.55565100	-1.73602400	1.28564500
H	1.22930600	-1.14002100	0.00000000
H	-0.22175300	-0.24412300	-2.17923400
H	-0.55565100	-1.73602400	-1.28564500
H	-1.76383000	-0.43113200	-1.32022800
Pd	2.26546000	0.05647300	0.00000000
H	3.97622800	2.72682700	-1.05031000
H	5.57546700	-0.82400300	0.00000000
H	3.97622800	2.72682700	1.05031000
H	2.32061200	3.48476900	0.00000000
H	4.38761400	-2.19829800	-1.05161400
H	4.38761400	-2.19829800	1.05161400
P	3.11381000	2.29771100	0.00000000
P	4.21180800	-1.25502100	0.00000000
C	-0.40828300	1.49759900	0.00000000
H	-0.02395300	2.00963200	-0.88875800
H	-0.02395300	2.00963200	0.88875800
H	-1.50909000	1.62283400	0.00000000

P: $Pd(PH_3)_2$ + $(CH_3)_3C–H$

E = -2343.91
H = -2224.40
G = -2258.47
N_{imag} = 0

C	-0.07624200	0.88371300	1.26610000
C	0.00365900	0.00835700	0.00000000
C	-0.07624200	0.88371300	-1.26610000
H	-0.08230400	0.28255600	2.18419600

H	0.80663800	1.54975300	1.31913600
H	-0.97260400	1.51174000	1.27318200
H	-2.46756200	0.01489400	0.00000000
H	-0.08230400	0.28255600	-2.18419600
H	-0.97260400	1.51174000	-1.27318200
H	0.80663800	1.54975300	-1.31913600
Pd	-1.69861600	-1.38780200	0.00000000
H	0.39774700	-3.78173600	-1.05664100
H	-4.32525400	-3.61883300	0.00000000
H	0.39774700	-3.78173600	1.05664100
H	-1.19400900	-4.68600500	0.00000000
H	-4.75109700	-1.83660500	-1.05777300
H	-4.75109700	-1.83660500	1.05777300
P	-0.49355600	-3.44701700	0.00000000
P	-3.89887000	-2.25291900	0.00000000
C	1.35119500	-0.73849200	0.00000000
H	1.48481400	-1.36445200	-0.89167900
H	1.48481400	-1.36445200	0.89167900
H	2.18152700	-0.00748800	0.00000000

RC: $PdCl^-$ + $H_3C{-}H$

E = -666.71
H = -634.77
G = -658.28
N_{imag} = 0

C	-1.81305100	-1.87786100	0.00000000
Pd	-0.21610400	-0.13180600	0.00000000
Cl	1.36511600	1.61397400	0.00000000
H	-2.08487400	-0.78245200	0.00000000
H	-2.22469800	-2.34081200	-0.90199200
H	-0.69121700	-2.02783800	0.00000000
H	-2.22469800	-2.34081200	0.90199200

TS: $PdCl^-$ + $H_3C{-}H$

E = -659.62
H = -630.18
G = -653.58
N_{imag} = 1, -658.32 cm^{-1}

C	-1.09073600	-2.12639400	0.00000000
Pd	-0.26199500	-0.15442100	0.00000000
Cl	1.26648800	1.68649000	0.00000000
H	-1.62780000	-2.47270200	0.89238600
H	-0.08032900	-2.56603800	0.00000000
H	-1.62780000	-2.47270200	-0.89238600
H	-1.72652800	-0.68559500	0.00000000

P: $PdCl^-$ + $H_3C{-}H$

E = -662.83
H = -632.81
G = -656.67
N_{imag} = 0

```
C  -0.89358500  -2.12916100   0.00000000
Pd -0.30643800  -0.15026900   0.00000000
Cl  1.31786900   1.65542900   0.00000000
H  -1.46633300  -2.41732100   0.89275000
H   0.07041400  -2.66508800   0.00000000
H  -1.46633300  -2.41732100  -0.89275000
H  -1.84541700  -0.14723700   0.00000000
```

RC: $PdCl^-$ + CH_3H_2C–H

E = -1028.11
H = -977.67
G = -1003.73
N_{imag} = 0

```
C  -0.97268000  -1.25140100   0.00000000
C  -0.06311500  -0.01012100   0.00000000
Pd  2.32714300  -0.00131100   0.00000000
H  -1.62303200  -1.27567300   0.88809900
H  -0.37338000  -2.16888300   0.00000000
H  -1.62303200  -1.27567300  -0.88809900
H  -0.63949300   0.92291000   0.00000000
H   0.58359700   0.00648600   0.92637700
H   0.58359700   0.00648600  -0.92637700
Cl  4.68016600  -0.00993900   0.00000000
```

TS: $PdCl^-$ + CH_3H_2C–H

E = -1020.06
H = -972.31
G = -997.63
N_{imag} = 1, -649.43 cm^{-1}

```
C  -0.10931400  -0.20280800  -2.06634900
Pd -0.12648300  -0.15780700   0.08180500
C   1.24926200  -0.15746000  -2.79173400
H   1.93608500   0.54379600  -2.30358700
H   1.74142700  -1.13960700  -2.79560800
H   1.13481200   0.16052600  -3.84479600
H  -0.58185500   0.79322600  -2.06368100
H  -0.79767600  -0.87415000  -2.59854100
H   0.08610300  -1.32030000  -0.93442600
Cl -0.27079300   0.64709700   2.32794400
```

P: $PdCl^-$ + CH_3H_2C–H

E = -1023.47
H = -975.13
G = -1001.23
N_{imag} = 0

```
C  -0.10348800   0.00964100  -0.21762100
Pd  1.52628400   0.19253700  -1.48089000
C  -0.38154800  -1.38530600   0.37310300
H  -0.37271400  -2.16343800  -0.40122200
H   0.37502700  -1.66370900   1.11793900
H  -1.37059300  -1.42739900   0.87099800
```

H	-0.90509100	0.29211600	-0.92287600
H	-0.09133900	0.76093000	0.58455000
H	2.11255000	0.09243500	-0.05325500
Cl	2.40358800	0.43956300	-3.73497100

RC: $PdCl^-$ + $(CH_3)_2HC-H$

E = -1390.87

H = -1322.33

G = -1350.62

N_{imag} = 0

C	-0.89270000	0.00012400	-1.28541100
C	-0.04769300	-0.00005300	0.00000000
C	-0.89270000	0.00012400	1.28541100
H	-0.24924200	-0.00013000	-2.17210500
H	-1.54333300	-0.88821500	-1.33615900
H	-1.54276500	0.88887100	-1.33627900
H	0.61050100	-0.91953700	0.00000000
H	0.61081700	0.91925400	0.00000000
H	-0.24924200	-0.00013000	2.17210500
H	-1.54276500	0.88887100	1.33627900
H	-1.54333300	-0.88821500	1.33615900
Pd	2.36272200	-0.00005800	0.00000000
Cl	4.71385500	0.00010300	0.00000000

TS: $PdCl^-$ + $(CH_3)_2HC-H$

E = -1381.95

H = -1316.34

G = -1343.51

N_{imag} = 1, -620.87 cm^{-1}

C	-0.75903800	-0.41857400	1.27457500
C	-0.00422800	-0.00022800	0.00000000
C	-0.75903800	-0.41857400	-1.27457500
H	-0.23096300	-0.08145800	2.17374000
H	-1.77815400	0.01410200	1.29166800
H	-0.86592300	-1.51078800	1.34626300
H	1.11855400	-1.17545200	0.00000000
H	-0.23096300	-0.08145800	-2.17374000
H	-0.86592300	-1.51078800	-1.34626300
H	-1.77815400	0.01410200	-1.29166800
H	0.08492300	1.09897900	0.00000000
Pd	2.15964200	-0.01623700	0.00000000
Cl	4.41219300	0.77850000	0.00000000

P: $PdCl^-$ + $(CH_3)_2HC-H$

E = -1385.73

H = -1319.48

G = -1347.66

N_{imag} = 0

C	-0.68785300	-0.33460600	1.27015500
C	0.03411800	0.14287000	0.00000000
C	-0.68785300	-0.33460600	-1.27015500

H	-0.18679000	0.02399300	2.17713200
H	-1.73492700	0.03262900	1.29408300
H	-0.72191700	-1.43056900	1.32440700
H	1.57427900	-1.54249700	0.00000000
H	-0.18679000	0.02399300	-2.17713200
H	-0.72191700	-1.43056900	-1.32440700
H	-1.73492700	0.03262900	-1.29408300
H	0.03678200	1.24867700	0.00000000
Pd	2.10008100	-0.08461400	0.00000000
Cl	4.22695800	1.09244800	0.00000000

RC: $PdCl^-$ + $(CH_3)_3C-H$

E = -1748.80

H = -1663.31

G = -1691.97

N_{imag} = 0

C	0.36386900	-1.03911000	0.00000000
C	1.90373700	-0.99994200	0.00000000
C	-0.17948000	-1.71646100	1.27200000
C	-0.17948000	-1.71646100	-1.27200000
H	0.00215900	0.02754000	0.00000000
H	2.28308600	-0.47641800	-0.88532600
H	2.28308600	-0.47641800	0.88532600
H	2.31496800	-2.02393400	0.00000000
H	-1.27542600	-1.70143800	-1.28729800
H	0.17374200	-1.20123800	-2.17282100
H	0.15382700	-2.76742400	-1.32050600
H	-1.27542600	-1.70143800	1.28729800
H	0.15382700	-2.76742400	1.32050600
H	0.17374200	-1.20123800	2.17282100
Pd	-0.64328100	1.75300700	0.00000000
Cl	-1.42987400	3.97810100	0.00000000

TS: $PdCl^-$ + $(CH_3)_3C-H$

E = -1741.29

H = -1657.74

G = -1687.00

N_{imag} = 1, -639.34 cm^{-1}

C	0.00147300	0.01642700	0.00000000
C	0.13987000	-1.52634300	0.00000000
C	0.70051900	0.56696600	-1.26318900
C	0.70051900	0.56696600	1.26318900
H	-1.11901800	1.05298100	0.00000000
H	-0.31530900	-1.98216700	0.88547700
H	-0.31530900	-1.98216700	-0.88547700
H	1.22232700	-1.79320900	0.00000000
H	0.68343900	1.66458200	1.29180000
H	0.68343900	1.66458200	-1.29180000
H	1.75931300	0.24220000	1.29108900
H	1.75931300	0.24220000	-1.29108900
H	0.20819500	0.20455600	-2.17293700

H	0.20819500	0.20455600	2.17293700
Pd	-2.24464700	-0.02253800	0.00000000
Cl	-4.52554100	-0.69920100	0.00000000

P: $PdCl^-$ + $(CH_3)_3C-H$

E = -1748.65

H = -1664.68

G = -1694.64

N_{imag} = 0

C	-0.11758500	-0.12900800	0.00000000
C	0.00384700	-1.66620700	0.00000000
C	0.55993000	0.43994800	-1.26098300
C	0.55993000	0.43994800	1.26098300
H	-1.71269200	1.58638600	0.00000000
H	-0.46437100	-2.11178400	0.88693900
H	-0.46437100	-2.11178400	-0.88693900
H	1.07218500	-1.97156800	0.00000000
H	0.52106000	1.53516500	1.27733600
H	0.52106000	1.53516500	-1.27733600
H	1.62829000	0.13616200	1.30174800
H	1.62829000	0.13616200	-1.30174800
H	0.07632700	0.07791300	-2.17639500
H	0.07632700	0.07791300	2.17639500
Pd	-2.20084000	0.11526700	0.00000000
Cl	-4.40453000	-0.91396600	0.00000000

References

[1] a) G. te Velde, F. M. Bickelhaupt, E. J. Baerends, C. Fonseca Guerra, S. J. A. van Gisbergen, J. G. Snijders, T. Ziegler, *J. Comput. Chem*. **2001**, *22*, 931–967; b) C. Fonseca Guerra, J. G. Snijders, G. te Velde, E. J. Baerends, *Theor. Chem. Acc*. **1998**, *99*, 391–403; c) ADF2018.105, SCM Theoretical Chemistry; Vrije Universiteit, Amsterdam, The Netherlands, http://www.scm.com.

[2] a) A. D. Becke, *Phys. Rev. A* **1988**, *38*, 3098–3100; b) B. G. Johnson, P. M. W. Gill, J. A. Pople, *J. Chem. Phys.* **1993**, *98*, 5612–5626; c) C. Lee, W. Yang, R. G. Parr, *Phys. Rev. B* **1988**, *37*, 785–789; d) T. V. Russo, R. L. Martin, P. J. Hay, *J. Chem. Phys.* **1994**, *101*, 7729–7737.

[3] S. Grimme, S. Ehrlich, L. Goerigk, *J. Comput. Chem.* **2011**, *32*, 1456–1465.

[4] a) A. Diefenbach, F. M. Bickelhaupt, *J. Phys. Chem. A*, **2004**, *108*, 8460–8466; b) G. T. de Jong, M. Solà, L. Visscher, F. M. Bickelhaupt, *J. Chem. Phys.* **2004**, *121*, 9982–9992; c) W.-J. van Zeist, A. H. Koers, L. P. Wolters, F. M. Bickelhaupt, *J. Chem. Theory Comput.* **2008**, *4*, 920–928

[5] E. van Lenthe, E. J. Baerends, J. G. Snijders, Relativistic total energy using regular approximations. *J. Chem. Phys.* **1994**, 101, 9783–9792.

[6] E. van Lenthe, E. J. Baerends, *J. Comput. Chem.* **2003**, *24*, 1142–1156.

[7] a) M. Franchini, P. H. T. Philipsen, E. van Lenthe, L. Visscher, *J. Chem. Theory Comput.* **2014**, *10*, 1994–2004; b) M. Franchini, P. H. T. Philipsen, L. Visscher, *J. Comput. Chem.* **2013**, *34*, 1819–1827.

[8] a) A. Bérces, R. M. Dickson, L. Fan, H. Jacobsen, D. Swerhone, T. Ziegler, *Comput. Phys. Commun.* **1997**, *100*, 247–262; b) H. Jacobsen, A. Bérces, D. P. Swerhone, T. Ziegler, *Comput. Phys. Commun.* **1997**, *100*, 263–276; c) S. K. Wolff, *Int. J. Quantum Chem.* **2005**, *104*, 645–659.

[9] a) K. Fukui, *Acc. Chem. Res.* **1981**, *14*, 363–368; b) L. Deng, T. Ziegler, L. A. Fan, *J. Chem. Phys.* **1993**, *99*, 3823–3835; c) L. Deng, T. Ziegler, *Int. J. Quantum Chem.* **1994**, *52*, 731–765.

[10] a) X. Sun, T. M. Soini, J. Poater, T. A. Hamlin, F. M. Bickelhaupt, *J. Comput. Chem.* **2019**, *40*, 2227–2233; b) PyFrag 2007–2022: X. Sun, T. Soini, L. P. Wolters, W.-J. van Zeist, C. Fonseca Guerra, T. A. Hamlin, F. M. Bickelhaupt, Vrije Universiteit Amsterdam, The Netherlands.

[11] C. Y. Legault, CYLview, 1.0b; Université de Sherbrooke, Canada, Sherbrooke, QC, 2009, http://www. cylview.org.

[12] P. Vermeeren, S. C. C. van der Lubbe, C. Fonseca Guerra, F. M. Bickelhaupt, T. A. Hamlin, *Nat. Protoc.* **2020**, *15*, 649–667.

[13] a) D. H. Ess, K. N. Houk, *J. Am. Chem. Soc.* **2007**, *129*, 10646–10647; b) D. H. Ess, K. N. Houk, *J. Am. Chem. Soc.* **2008**, *130*, 10187–10198.

[14] a) F. M. Bickelhaupt, E. J. Baerends, *Reviews in Computational Chemistry*; K. B. Lipkowitz, D. B. Boyd, Wiley-VCH: New York, **2000**; Vol. *15*, pp 1–86; b) R. van Meer, O. V. Gritsenko, E. J. Baerends, *J. Chem. Theory Comput.* **2014**, *10*, 4432–4441.

[15] a) P. W. Atkins, J. de Pauli, *Physical Chemistry*, Edn. 9, Oxford University Press, Oxford, **2010**; F. Jensen, *Introduction to Computational Chemistry*, Edn. 2, Wiley, West Sussex, **2007**.
